# Et_3_N/DMSO-supported one-pot synthesis of highly fluorescent β-carboline-linked benzothiophenones via sulfur insertion and estimation of the photophysical properties

**DOI:** 10.3762/bjoc.16.146

**Published:** 2020-07-20

**Authors:** Dharmender Singh, Vipin Kumar, Virender Singh

**Affiliations:** 1 Department of Chemistry, Dr. B R Ambedkar National Institute of Technology (NIT), Jalandhar, 144011, Punjab, India; 2 Department of Chemistry, Central University of Punjab, Bathinda, 151001, Punjab, India

**Keywords:** benzothiophene, β-carboline, metal-free, photophysical properties, sulfur insertion

## Abstract

A robust transition-metal-free strategy is presented to access novel β-carboline-tethered benzothiophenone derivatives from 1(3)-formyl-β-carbolines using elemental sulfur activated by Et_3_N/DMSO. This expeditious catalyst-free reaction proceeds through the formation of β-carboline-based 2-nitrochalcones followed by an incorporation of sulfur to generate multifunctional β-carboline-linked benzothiophenones in good to excellent yields. The synthetic strategy could also be extended towards the synthesis of β-carboline-linked benzothiophenes. Moreover, the afforded products emerged as promising fluorophores and displayed excellent light-emitting properties with quantum yields (Φ_F_) up to 47%.

## Introduction

**Figure 1:**
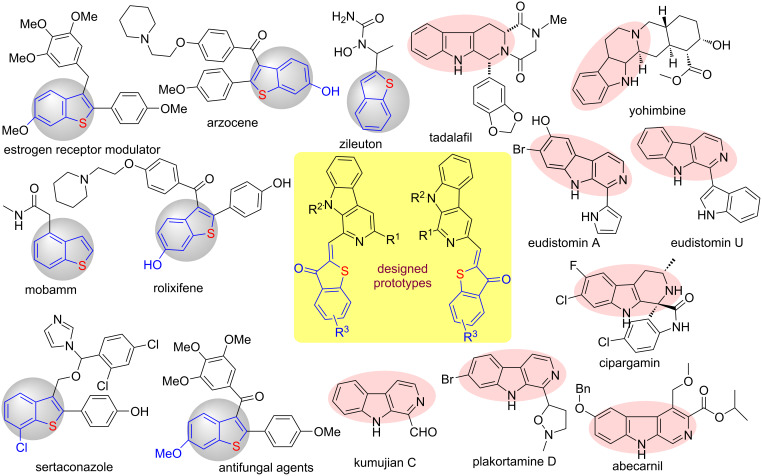
Representative examples of some commercial drugs and biologically active alkaloids.

The pyrido[3,4-*b*]indole moiety, commonly referred as β-carboline, is the core unit of about one quarter of all natural products [1–4] and pharmacologically active compounds endowed with anticancer [5–9], anti-inflammatory, antioxidant, antimalarial, antifungal, and antileishmanial activities (Figure 1) [10–13]. Notably, this privileged scaffold is incorporated in several marketed drugs such as abecarnil, tadalafil, cipargamin, yohimbine, etc. which are used in the treatment of various ailments [14–15]. Apart from their pharmaceutical properties, β-carboline derivatives also found various applications in fields such as organocatalysts, as ligands, and fluorescent probes [16–18]. Importantly, β-carbolines are also used as fluorescence standards. Recently, a novel β-carboline-based fluorescent chemosensor was developed by Batra and co-workers for the quantitative analysis of fluoride ions (F^−^) at ppb level [19].

Sulfur-containing organic compounds are broadly associated with numerous bioactive natural products and pharmaceutical drugs [20–22]. Thioaurones (2-benzylidene benzo[*b*]thiophen-3(2*H*)-one) are sulfur-containing heterocyclic compounds, an important subclass of flavonoids which were first introduced by O’Sullivan in 1977 [23]. Specifically, thioaurones and their analogs show a variety of biological activities such as anticancer [24], inhibition of tyrosine phosphatase 1B, antioxidant properties, etc. [25–27]. Due to their numerous applications, they have found diverse uses such as thioindigo-like dyes, photoresponsive devices, and photoswitchable biomolecules [28–31]. Moreover, these compounds were also used as synthetic intermediates for various sulfur-containing bioactive molecules (Figure 1) [32–34].

In organic synthesis, aromatic compounds having nitro groups play a vital role as building blocks for the synthesis of nitrogen-containing functional groups and aza-heterocyclic frameworks. However, organic transformations in which aromatic nitro groups act as leaving groups are less reported and require the use of transition-metal catalysts such as Cu, Rh, Pd, etc. [35–37]. Though, several elegant methods have been developed for the synthesis of benzothiophenes, however, these methods rely on the use of organosulfur-based substrates [38–41]. Moreover, these methods are associated with some limitations such as using costly metal catalysts, air-sensitive starting materials, malodorous sulfides or thiols, low yields, and multistep syntheses. To overcome these drawbacks, elemental sulfur has emerged as a surrogate approach, where it can be inserted in situ. In this context, several research groups are actively involved in the development of novel and efficient approaches for the synthesis of sulfur-containing heterocycles [42–46].

In our research endeavors, we have been involved in the exploration of the synthetic potential of 1-formyl-9*H*-β-carboline (an alkaloid, kumujian C) [47] for preparing chemical libraries of β-carboline-substituted [48–54] and N-fused heterocycles [55–56] which were attributed to the presence of an electrophilic as well as a nucleophilic functionality in this natural product [1]. Encouraged by the applications of β-carboline and benzothiophene motifs in medicinal and materials chemistry, it was envisaged to construct a β-carboline-based novel molecular hybrid containing the benzothiophene moiety (Figure 1) [57–58]. The present study was inspired by the recent findings of Nguyen and co-workers [59–61]. As a part of our ongoing project [62–63], we devised a simple and efficient one-pot practical approach for the construction of β-carboline-tethered benzothiophenone derivatives via incorporation of sulfur. To the best of our knowledge, this is the first report of one-pot synthesis of novel β-carboline-tethered benzothiophenones and evaluation of their light-emitting properties. In this regard, detailed studies are presented and discussed herein.

## Results and Discussion

**Scheme 1:**
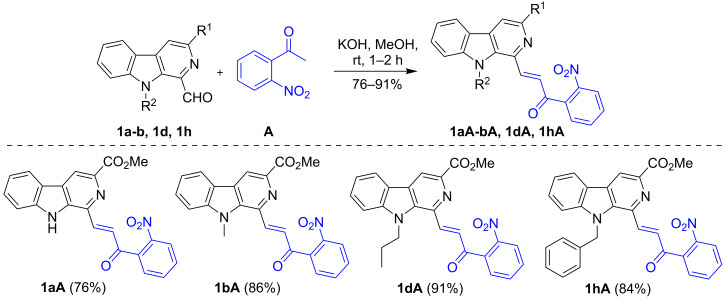
Synthesis of β-carboline-linked 2-nitrochalcones.

The present study commenced with the synthesis of the β-carboline-based 2-nitrochalcone **1bA** which was prepared via a Claisen–Schmidt condensation of 1-formyl-β-carboline (**1b**) with 2-nitroacetophenone (**A**) in the presence of KOH (1.05 equiv) in dry MeOH at room temperature (Scheme 1). The analytically pure product was obtained in 86% yield by simple filtration of the precipitate followed by washing with dry MeOH.

With the objective to synthesize β-carboline-linked benzothiophenone frameworks, we set up screening of conditions for the reaction between the β-carboline-based 2-nitrochalcone **1bA** and elemental sulfur by testing different activators (Table 1). Recent findings revealed that a combination of an aliphatic amine with DMSO activated elemental sulfur for an electrophilic addition to generate thioaurones and benzothiophenes [59]. At 70 °C, the use of DIPEA in combination with DMF as the solvent was found to be an excellent sulfur activator, leading to the formation of the desired product **2bA** in 50% yield after a short silica gel column chromatographic separation (Table 1, entry 1). The structure of **2bA** was confirmed on the basis of spectroscopic data. The ^1^H NMR spectrum displayed a singlet for one methine proton at δ 8.69 ppm and the presence of additional nine aromatic protons for the β-carboline and benzothiophenone frameworks indicated the formation of the desired product. When elemental sulfur and DIPEA were used in DMSO, a significant increase in the yield (73%) was observed (Table 1, entry 2). At this stage, we realized that DMSO was a better choice for this transformation as the reaction required a shorter time and afforded the product **2bA** in a better yield. Then, other amines such as NMP, Et_3_N, DBU, and DABCO were also investigated. The use of NMP as an activator in DMSO yielded the desired product in only 65% yield (Table 1, entry 3). Interestingly, Et_3_N in combination with DMSO at 70 °C afforded the anticipated product **2bA** in 78% yield within a short span of 20 min (Table 1, entry 4). The reaction at 90 °C gave product **2bA** in 76% yield, however, the same reaction performed at 30 °C was found to be sluggish and complete conversion could not be achieved even after 12 h (Table 1, entries 5 and 6). We also observed that the reaction in the absence of Et_3_N failed to generate the anticipated product **2bA** (Table 1, entry 7), which supported the importance of an amine/base with DMSO as an activator. Potassium ethylxanthate [64–65] and sodium sulfide as a sulfur sources in the presence of Et_3_N in DMSO (Table 1, entries 8 and 9) also did not furnish the desired product **2bA**. A decomposition of the product was observed in the case of Na_2_S. Interestingly, the combination of DABCO and DMSO also afforded the desired product via a clean reaction within 20 min, although only 60% yield of the product was obtained (Table 1, entry 10). This promising result using DABCO encouraged us to explore other amines like DBU, ʟ-proline, pyridine, DMAP, and pyrrolidine as an activator but encouraging results were not obtained (Table 1, entries 11–15). Similarly, the use of KI as an activator also failed to promote the reaction (Table 1, entry 16) [62]. Eventually, we came to the conclusion that a combination of Et_3_N and DMSO excellently activated elemental sulfur at 70 °C and therefore chose these conditions for the construction of other β-carboline-linked benzothiophenone derivatives.

**Table 1:** Optimization of the reaction conditions.^a^

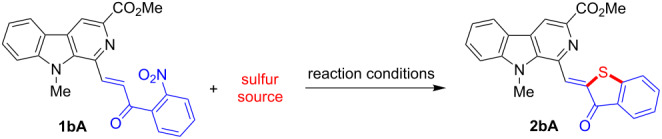						

entry	base^b^	sulfur source	solvent^c^	temperature (°C)	time	yield^d^ (%) of **2bA**

1	DIPEA	S_8_	DMF	70	1 h	50
2	DIPEA	S_8_	DMSO	70	45 min	73
3	NMP	S_8_	DMSO	80	1 h	65
**4**	**Et_3_N**	**S_8_**	**DMSO**	**70**	**20 min**	**78**
5	Et_3_N	S_8_	DMSO	90	18 min	76
6	Et_3_N	S_8_	DMSO	30	12 h	45
7^e^	–	S_8_	DMSO	90	12 h	NR
8^e^	Et_3_N	C_2_H_5_OCS_2_K	DMSO	60	10 h	NR
9^f^	Et_3_N	Na_2_S	DMSO	60	20 h	–
10	DABCO	S_8_	DMSO	60	20 min	60
11	DBU	S_8_	DMSO	70	40 min	30 + impurity
12	L-proline	S_8_	DMSO	70	3.5 h	30 + **1bA**
13	pyridine	S_8_	DMSO	70	12 h	NR
14	DMAP	S_8_	DMSO	70	3 h	35 + **1bA**
15	pyrrolidine	S_8_	DMSO	60	3 h	40
16^e^	KI	S_8_	DMSO	70	2.5 h	NR

^a^All reactions were performed with 0.12 mmol of **1bA**, 0.60 mmol (5.0 equiv) of sulfur powder, and 0.60 mmol (5.0 equiv) of amine/base in 0.5 mL of solvent; ^b^5.0 equiv of amine/base were used except KI (3.0 equiv), L-proline (4.0 equiv), and DMAP (1.5 equiv); ^c^dry solvents were used; ^d^isolated yields of the purified product; ^e^NR = no reaction was observed; ^f^decomposition of starting material was observed.

**Scheme 2:**
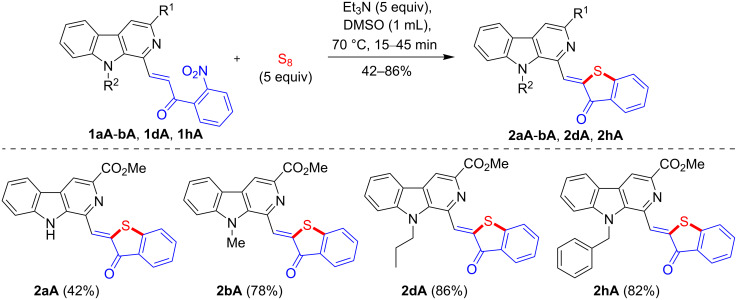
Synthesis of β-carboline-linked benzothiophenone frameworks.

With the standardized conditions identified, the scope of this domino approach was investigated with diversely substituted β-carboline-based 2-nitrochalcones **1aA-bA**, 1**dA**, and **1hA** prepared from aldehydes **1a**-**b**, **1d** and **1h** in 76–91% yields (Scheme 1). The methodology was found to be general in nature and produced the fluorescent β-carboline-linked benzothiophenone derivatives **2aA**-**bA**, **2dA** and **2hA** within 15–45 min in DMSO at 70 °C as depicted in Scheme 2. The analytically pure products were obtained in 42–86% yields after a short silica gel column chromatographic purification. It was observed that *N*-alkyl-β-carboline-based 2-nitrochalcones **1bA**, **1dA,** and **1hA** reacted faster and delivered the products **2bA**, **2dA**, and **2hA** in better yields (78-86%). Conversely, the substrate **1aA** bearing a free NH was found to be slow reacting and produced **2aA** in a lower yield (42%).

**Scheme 3:**
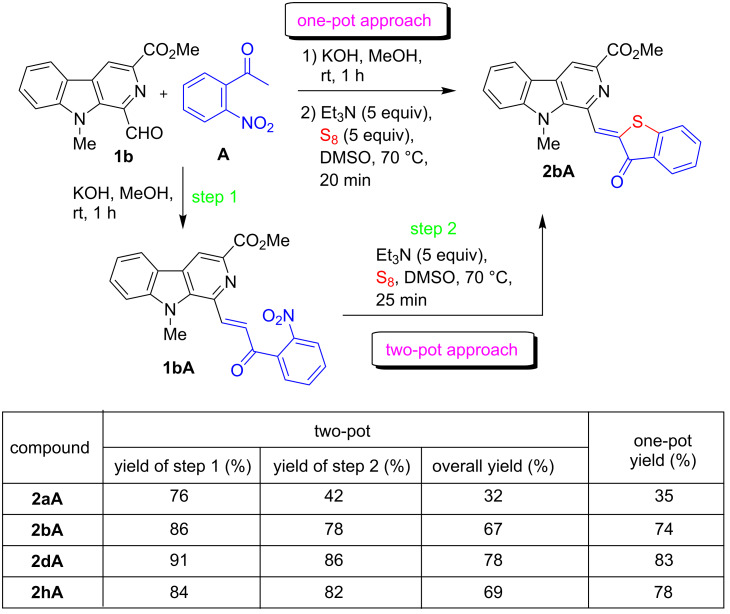
Comparison of outcome of one-pot vs two-pot approach.

From the perspective of green chemistry, one-pot reactions are preferred as less waste is generated due to the avoidance of work-up, isolation, and purification of intermediates [66]. Accordingly, the feasibility of a one-pot synthesis of the targeted products was attempted. Therefore, after the formation of the 2-nitrochalcone **1bA**, excess of MeOH was decanted, and the crude product was redissolved in 1 mL of DMSO followed by the sequential addition of Et_3_N (5 equiv) and elemental sulfur (5 equiv). To our pleasure, the reaction at 70 °C smoothly afforded the corresponding β-carboline-linked benzothiophenone derivative **2bA** in less than 30 min. More importantly, a clean reaction was observed during the one-pot strategy which surely avoided the isolation of the intermediate (2-nitrochalcone derivative **1bA**), and a significant increment in the overall yield of **2bA** (from 67% to 74%) was also noted. Similarly, a remarkable improvement in the yields of **2aA** (from 32% to 35%), **2dA** (from 78% to 83%), and **2hA** (from 69% to 78%) was also observed during the one-pot approach. The comparison of the product yields obtained through both approaches are summarized in Scheme 3.

**Scheme 4:**
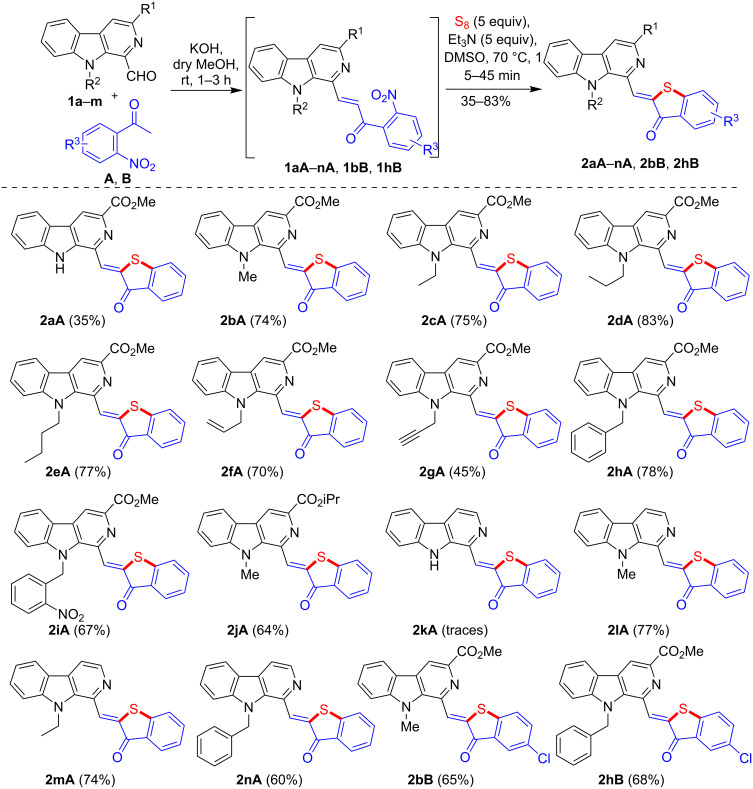
One-pot synthesis of β-carboline C-1-tethered benzothiophenone derivatives.

Having successfully established a one-pot strategy, we next turned our attention to the generality and scope of the method. Interestingly, diversely substituted 1-formyl-β-carbolines **1a**–**m** (except **1k**) reacted efficiently with nitroacetophenones **A**,**B** in one pot furnishing the anticipated products **2aA**–**nA**, **2bB**, and **2hB** as depicted in Scheme 4. The synthesized products were purified through silica gel column chromatography and further washed with anhydrous methanol to yield the analytically pure products in 35–83% yields (two-step yield), except for **2kA**, which was obtained in trace amounts only. We observed that *N*-alkyl derivatives **1bA**–**jA**, **1lA**–**nA**, **1bB**, and **1hB** reacted faster and led to higher product yields. The substrate **B** bearing a chloro substituent required longer reaction times and afforded the targeted products **2bB** and **2hB** in slightly lower yields.

**Scheme 5:**
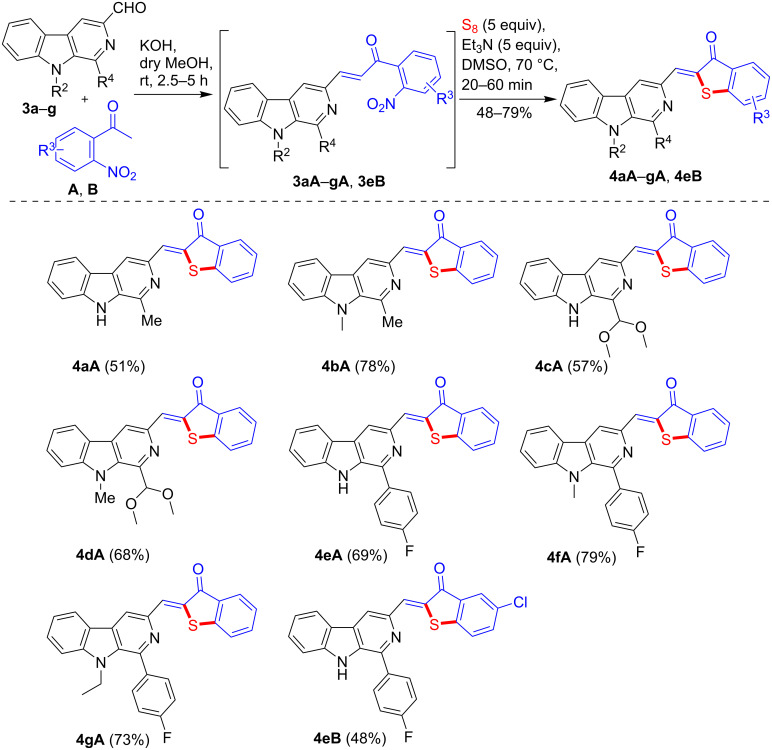
One-pot synthesis of β-carboline C-3-linked benzothiophenone derivatives.

Encouraged by the results obtained from the one-pot synthesis of β-carboline C-1 substituted benzothiophenone derivatives, we were interested if the scope of this one-pot strategy could be extended for the synthesis of β-carboline C-3-tethered benzothiophenones (Scheme 5). Thus, the Claisen–Schmidt condensation of 3-formyl-9*H*-β-carbolines **3a**–**g** [51] with substituted 2-nitroacetophenones (**A** and **B**) in the presence of KOH delivered the corresponding 2-nitrochalcones (**3aA**–**gA** and **3eB**). The in situ-generated β-carboline-based 2-nitrochalcones were further treated with elemental sulfur in the presence of Et_3_N in DMSO at 70 °C straightforwardly affording the cyclized products **4aA**–**gA** and **4eB** in 48–79% yield as presented in Scheme 5.

**Figure 2:**
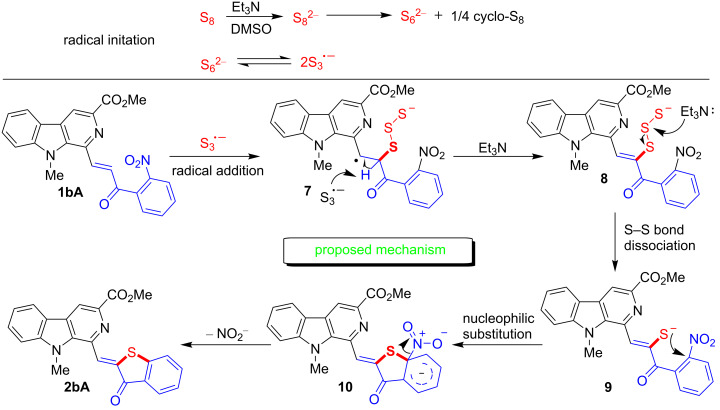
Proposed reaction mechanism.

It was observed that the reaction time and yields were affected by the nature of the substituent at the C1 (R^4^) and N-9 (R^2^) position of β-carboline ring. Substrates bearing a dimethoxymethyl group (**3c** and **3d**) reacted smoothly and within shorter reaction time. Similarly, *N*-alkyl (R^2^) 3-formyl-β-carbolines (**3b**, **3d**, **3f**, and **3g**) also reacted faster and delivered higher yields as compared to free NH derivatives (**3a**, **3c**, and **3e**). In case of the dihalogenated product **4eB**, a slow reaction accompanied with a low yield was detected due to presence of electron-withdrawing substituents in starting compound **B**. The slightly lower yields obtained for **2bB**, **2hB**, and **4eB** were possibly due to the low reactivity of substrate **B** during the condensation process (step 1), as in the cyclization process, the presence of the electron-withdrawing substituents in **B** seemed to favor the anticipated S_N_Ar mechanism by stabilizing the negatively charged intermediate **10** (Figure 2). Overall, it was noted that the 1-formyl-β-carbolines **1a**–**m** reacted faster and afforded the corresponding products in higher yields as compared to 3-formyl-β-carbolines **3a**–**g** which may be attributed to the higher electrophilicity of the formyl group at C1 position of the β-carboline ring.

**Scheme 6:**
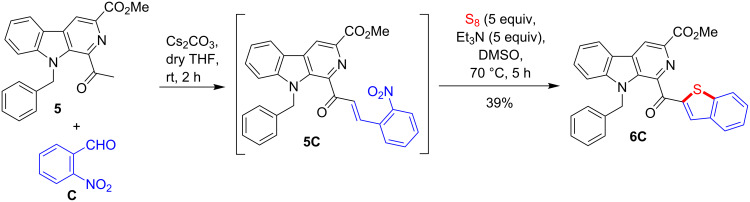
One-pot synthesis of β-carboline-linked benzothiophene derivative **6C**.

Inspired by the results of the above study, it was envisaged to check the generality of this strategy for the synthesis of β-carboline linked benzothiophene derivatives. Accordingly, we employed 1-acetyl-β-carboline **5** [67–68] for Claisen–Schimdt condensation with 2-nitrobenzaldehyde (**C**) in the presence of Cs_2_CO_3_ and anhydrous THF at room temperature. Product **5C** was obtained as a major product along with some unidentified impurities. The evaporation of excess solvent (THF) followed by the treatment of the resultant crude, 2-nitrochalcone **5C** with Et_3_N and elemental sulfur in DMSO at 70 °C furnished the expected product **6C**, albeit in a low yield (39%) as shown in Scheme 6. It is important to mention that the Claisen–Schmidt condensation of **5** with 2-nitrobenzaldehyde (**C**) could not be achieved with KOH in MeOH or Cs_2_CO_3_ in DMSO.

**Scheme 7:**
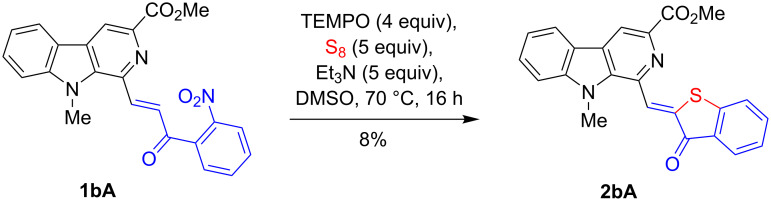
Control experiment in the presence of a radical scavenger.

To probe the reaction mechanism, a control experiment was conducted with model substrate **1bA** in the presence of a radical scavenger (TEMPO) to check the possibility of a radical mechanism vs an electrophilic addition of sulfur [69] (Scheme 7). It was observed that the reaction could not be completed even after 24 h in the presence of TEMPO whereas only 20 min were required for completion under standard conditions. Thus, it is assumed that the reaction proceeds through a radical pathway.

Based on our observations during the present study and previous reports [69], a plausible mechanism for the formation of the benzothiophenone ring is depicted in Figure 2. It is anticipated that an initial formation of trisulfur radical anion (S_3_**^·−^**) occurs via the reaction of elemental sulfur with triethylamine in DMSO. The addition of the trisulfur radical anion to the double bond in 2-nitrochalcone (**1bA**) may yield the intermediate **7**. The further abstraction of hydrogen in intermediate **7** may result in formation of intermediate **8**. The cleavage of the S–S bond in **8** under basic conditions may generate sulfur anion **9**. The nucleophilic substitution reaction (S_N_Ar) by transit the sulfur anion in **9** followed by dismissal of the nitrite ion may result in the formation of the β-carboline-tethered benzothiophenone derivative **2bA**. It is anticipated that the role of DMSO is to stabilize the ionic intermediates, specifically **10** and to accelerate the transformation.

### Photophysical studies

Fluorescence, offering a nondestructive exceptional technique to monitor a system of interest at the molecular level [70–72], has found wide-ranging applications in several research areas such as medicine, pharmaceutics, biology, environment, and food science [73–74]. Therefore, the light-emitting properties of the novel β-carboline C1 as well as C3-substituted benzothiophenone derivatives **2aA**–**nA**, **2bB**, **2hB**, **4aA**–**gA**, **4eB**, and **6C** were evaluated to stimulate their further exploration for possible applications in the field of materials science as chemosensors, ligands, and fluorescent probes. In order to investigate the fluorescence properties, compound **2bA** was chosen as the model substrate for optimization of various parameters like time, concentration, and solvent. In order to obtain the maximum emission, various solvents were screened. The synthesized compounds showed best solubility and displayed a maximum intensity in CHCl_3_ (Supporting Information File 1) as compared to other solvents (DMSO, DMF, and MeOH). No considerable change in the fluorescence intensity of was observed even after 5 h of sample preparation. Next, after careful analysis of concentrations, a 4 µM concentration in CHCl_3_ was found to be optimal for the photophysical studies of the synthesized derivatives.

**Figure 3:**
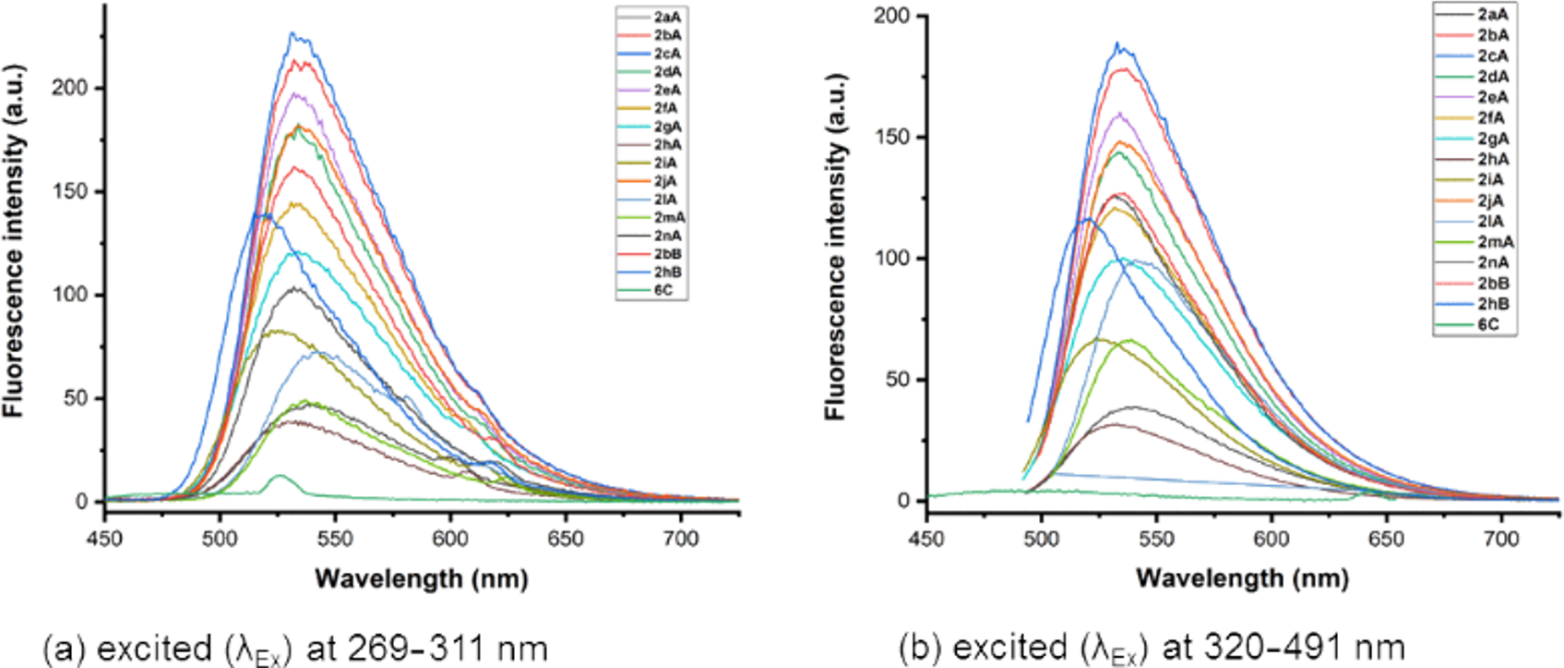
Fluorescence spectra of **2aA**–**nA**, **2bB**, **2hB**, and **6C**.

**Figure 4:**
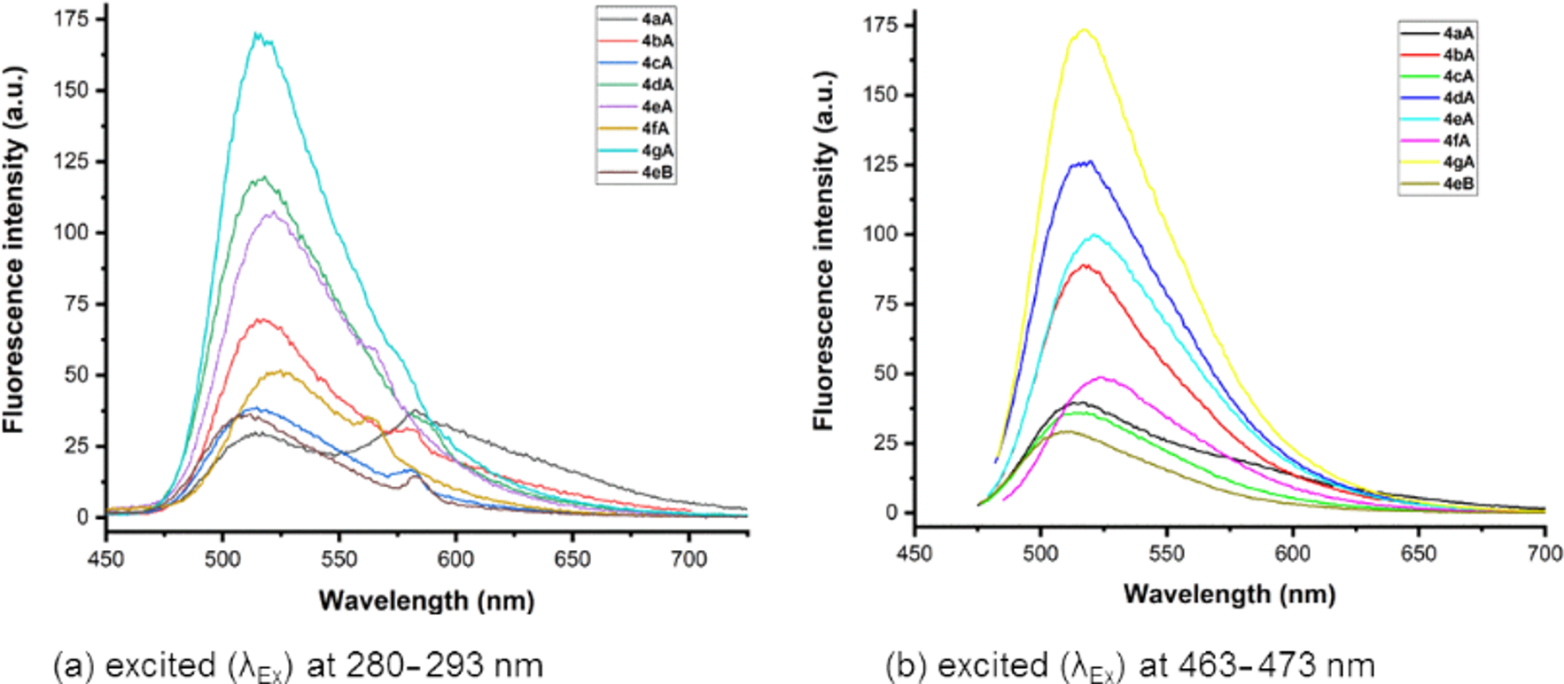
Fluorescence spectra of **4aA**–**gA**, and **4eB**.

The fluorescence quantum efficiency (Φ_F_) was measured relative to quinine sulfate (Φ_R_ = 0.546 in 0.1 M H_2_SO_4_ under 350 nm excitation) as a reference compound. For the measurement of UV–vis absorption and fluorescence emission of the samples, stock solutions of 1.0 mM concentration were prepared using analytical grade CHCl_3_ as the solvent, and diluted to the final concentration of 4.0 μM. Next, we carefully measured the photophysical properties at room temperature including absorption, excitation, emission, Stokes shift, fluorescence quantum efficiency, molar extinction coefficient, and brightness. The photophysical data of the β-carboline C1 or C3-tethered benzothiophenone derivatives are summarized in Table 2, and their graphical data are depicted in Figure 3 and Figure 4. The quantum yields were calculated based on Equation 1.

[1] $$\Phi_{\text{S}}=\Phi_{\text{R}}\times \frac{I_{\text{S}}}{I_{\text{R}}}\times \frac{A_{\text{R}}}{A_{\text{S}}}\times \frac{{\text{η}}_{\text{S}}^{2}}{{\text{η}}_{\text{R}}^{2}}$$

where R means reference and S sample, respectively.

Generally weak fluorescence emissions were observed for the thiophene-based chromophores due to a remarkable spin–orbit coupling which is originating from the heavy atom effect of sulfur [73–74]. It is worth mentioning that the majority of the β-carboline-substituted benzothiophenone derivatives showed good fluorescence. The emission maxima of the fluorophores showed a wide region for fluorescent emissions (λ_em_, 490–582 nm in CHCl_3_) along with large Stokes shifts (up to 293 nm), excellent quantum yields (up to 47%), and high brightness (up to 11196). The brightness of the fluorophores was calculated by multiplication of the quantum yield (Φ) with its molar extinction coefficient (ε).

In case of the β-carboline C1-substituted benzothiophenones **2aA**–**nA**, **2bB**, and **2hB**, the substituents R^1^ and R^2^ significantly affected the fluorescence of the compounds. It was noted that the fluorescence increased with lengthening of the alkyl chain at N-9 (R^2^) and followed the order *n*-Bu > *n*-Pr > Et > Me > H. The β-carboline derivative with free N–H (N-9), **2aA**, showed a low fluorescence quantum yield (Φ_F_ = 18%) in this series (Table 2 and Figure 3). The presence of a benzyl group at R^2^ (**2hA**), improved the photophysical properties including a higher quantum yield (Φ_F_ = 47%). With variation of the substituents at the R^1^ position, a regular pattern of fluorescence was observed, i.e., CO_2_iPr > CO_2_Me > H which may be attributed to the electron-withdrawing nature of the substituents (ester group) at C3 position of the β-carboline ring.

**Table 2:** Photophysical data of β-carboline-tethered benzothiophenone derivatives.

compound	UV–vis^a^	fluorescence			Stokes shift (nm)	molar extinction coefficient (ε) (M^−1^·cm^−1^)	brightness
	λ_Ex_ (nm)	λ_Em_ (nm)	intensity (a.u.)	quantum yield (Φ_F_)^b^			

**2aA**	300.50 481.00	539.95 540.88	47.61 38.78	0.186 0.295	239.45 59.88	13750 6750	2557 1991
**2bA**	305.20 487.78	531.94 536.86	213.81 178.31	0.229 0.342	226.74 49.08	45750 25000	10477 8550
**2cA**	305.40 488.18	533.89 532.83	233.89 193.36	0.265 0.347	234.49 44.65	42250 26250	11196 9109
**2dA**	306.13 487.50	534.02 534.02	182.87 143.78	0.274 0.383	227.72 47.42	31000 17250	8494 6607
**2eA**	304.93 487.49	531.94 534.02	197.94 160.50	0.279 0.400	227.01 46.53	33250 18250	9277 7300
**2fA**	303.41 483.69	530.89 531.94	144.88 121.01	0.256 0.374	227.48 48.25	27750 15500	7104 5797
**2gA**	303.61 480.49	534.02 535.07	121.25 100.11	0.221 0.328	230.41 54.58	28500 15500	6298 5084
**2hA**	303.21 482.90	532.98 532.83	39.57 31.74	0.302 0.473	229.77 49.93	6750 3250	2038 1537
**2iA**	306.21 480.08	525.07 524.91	83.13 67.85	0.243 0.337	218.86 44.83	17000 9500	4131 3201
**2jA**	305.89 487.51	534.02 534.98	181.82 148.43	0.304 0.378	228.13 47.47	29250 18750	8892 7087
**2lA**	289.98 492.53	542.05 541.08	72.89 99.43	0.189 0.253	252.07 48.55	19500 18500	3685 4680
**2mA**	311.59 491.81	537.01 538.95	49.49 66.73	0.223 0.366	225.42 47.14	10250 8000	2286 2928
**2nA**	309.56 487.74	531.94 531.79	104.13 126.29	0.213 0.349	222.38 44.05	22750 16250	4846 5671
**2bB**	309.67 486.62	531.94 535.97	162.21 127.26	0.301 0.378	222.27 49.35	25500 15500	7675 5859
**2hB**	308.54 482.71	515.78 521.54	140.57 116.95	0.330 0.258	207.24 38.83	18750 8000	6187 2064
**4aA**	289.82 463.50	522.94 512.83	37.76 39.76	0.052 0.095	293.12 49.33	60750 24250	3159 2304
**4bA**	289.62 380.09 470.77	514.64 518.04 517.02	69.79 40.58 89.14	0.167 0.179 0.219	225.02 137.95 46.25	22500 10750 17750	3757 1924 3887
**4cA**	289.17 465.41	514.91 518.05	38.82 36.06	0.079 0.179	225.74 52.64	25000 9250	1975 1656
**4dA**	293.41 470.59	518.05 520.01	119.83 126.18	0.160 0.291	224.64 49.42	35750 19500	5720 5674
**4eA**	282.56 376.41 466.89	521.94 519.85 521.04	107.64 63.43 99.98	0.111 0.141 0.196	239.38 143.44 54.15	46750 23500 23250	5189 3313 4557
**4fA**	280.74 383.74 473.79	524.92 520.89 522.98	51.79 29.12 48.80	0.080 0.141 0.158	244.18 137.15 49.19	32500 11750 13750	2600 1657 2172
**4gA**	288.53 380.11 471.56	514.02 517.01 517.90	170.41 86.20 173.56	0.252 0.284 0.257	225.49 136.90 46.34	29500 13000 21500	7437 3692 5525
**4eB**	291.19 464.27	511.94 508.04	36.39 29.47	0.150 0.225	220.75 43.77	12250 4250	1837 956
**6C**	263.14 320.64	525.96 490.41	13.60 4.48	0.034 0.016	262.82 169.77	15000 18750	510 300

^a^Measured at 4 µM concentration in CHCl_3_; ^b^quantum yields (Φ_F_) were determined with reference to quinine sulfate.

Interestingly, a similar trend was observed in case of β-carboline C3-substituted benzothiophenone derivatives (**4aA**–**gA** and **4eB**). Compared to **4aA** and **4aC** bearing aliphatic substituents at C1 (R^4^), compound **4eA** with aromatic substituent exhibited better fluorescence due to extended conjugation. The effect of R^2^ substituent in these derivatives (**4bA**, **4dA**, **4fA** and **4gA**) was also investigated and it was found that *N*-alkylation improved the photophysical properties along with higher quantum yields (Figure 4). With regard to the impact of R^3^ substituent, thiopheneone derivatives with chloro substitution (**2bB** and **4eB**) displayed a higher quantum yield than unsubstituted derivatives (**2bA** and **4eA**) as evident from Table 2. Overall, it can be concluded that β-carboline C1 substituted benzothiophenone derivatives exhibited better photophysical properties including high quantum yield, brightness and significant bathochromic shift in the emission wavelengths. In short, β-carboline-substituted benzothiophenone derivatives emerged as excellent fluorophores and displayed remarkable photophysical properties with quantum yield (Φ_F_) up to 47%. It is believed that these compounds may find applications in materials science and biomedical investigations.

## Conclusion

In summary, an efficient synthesis of highly fluorescent β-carboline-linked benzothiophenone derivatives was successfully accomplished through a one-pot metal-free approach for the first time. The transformation could be executed from β-carboline-based 2-nitrochalcones via a one-pot, two-step procedure starting from 1(3)-formyl-β-carbolines (a framework represented by alkaloid kumujian C). The combination of Et_3_N and DMSO played a vital role in the activation of sulfur resulting in the formation of two C–S bonds in a single operation. This strategy offers several advantages, such as one-pot procedure, operational simplicity, easy purification, use of inexpensive reagents, and wide functional group compatibility. Importantly, the presence of two important pharmacophores along with the exocyclic double bond with Michael acceptor properties in the title compounds offers the opportunity to explore their biological potential. Moreover, these β-carboline-linked benzothiophenones displayed excellent fluorescence properties with quantum yields (Φ_F_) of up to 47%. Detailed studies to synthesize novel fluorophores with improved optical properties which can easily find application in materials science are underway in our laboratory

## Experimental

**General experimental procedure for the synthesis of β-carboline-based 2-nitrochalcone derivatives (1aA, 1bA, 1dA and 1hA) as exemplified for compound 1bA**. To a stirred solution of KOH (0.033 g, 0.587 mmol) in dry MeOH (4 mL), 2-nitroacetophenone (**A**, 0.080 mL, 0.587 mmol) was added at room temperature, and the reaction mixture was stirred for 15 min. Thereafter, **1b** (0.15 g, 0.560 mmol) was added portionwise and the reaction mixture was allowed to stir for an additional 1 h at room temperature. After completion of the reaction (as monitored by TLC), the precipitate was filtered through a sintered funnel, washed twice with anhydrous MeOH, and dried in vacuum to obtain the analytically pure product **1bA**, 0.20 g (86%) as yellow solid.

**General experimental procedure for the synthesis of β-carboline C-1-substituted benzothiophenone derivatives (2aA, 2bA, 2dA, and 2hA) as exemplified for compound 2bA.** A 10 mL round-bottomed flask was charged with 2-nitrochalcone **1bA** (0.20 g, 0.482 mmol), Et_3_N (0.336 mL, 2.41 mmol), sulfur powder (0.077 g, 2.41 mmol), DMSO (1 mL), and the reaction mixture was stirred at 70 °C for 20 min. After completion of the reaction, as analyzed by TLC, the crude product was directly purified by silica gel column chromatography (CHCl_3_/MeOH 95:5, v/v) without aqueous treatment to afford 0.15 g of **2bA** (78%) as orange solid.

**One-pot experimental procedure for the synthesis of β-carboline C-1(3)-substituted benzothiophenone derivatives (2aA**–**nA**, **2bB**, **2hB**, **4aA**–**gA, and 4eB) as exemplified for compound 2bA.** To a stirred solution of KOH (0.033 g, 0.587 mmol) in dry MeOH (4 mL) in a 10 mL round-bottomed flask; 2-nitroacetophenone (0.080 mL, 0.587 mmol) was added at room temperature and the reaction mixture was stirred for 15 min. Thereafter, methyl 1-formyl-9-methyl-9*H*-pyrido[3,4-*b*]indole-3-carboxylate (**1b**, 0.15 g, 0.560 mmol) was added portionwise and the reaction mixture was allowed to stir for an additional 1 h at room temperature. After completion of the reaction (as detected by TLC), the reaction content was allowed to settle for 5 min, MeOH was decanted, and evaporated under reduced pressure. Thereafter, DMSO (1.5 mL) was added to the crude product **1bA** (nitrochalcone) followed by the sequential addition of sulfur powder (0.089 g, 2.80 mmol) and Et_3_N (0.390 mL, 2.80 mmol) at room temperature. The reaction mixture was stirred at 70 °C for 20 min. After completion of the reaction (as analyzed by TLC), the product **2bA** was directly purified through column chromatography on silica gel (CHCl_3_/MeOH 95:5, v/v) to afford the analytically pure product **2bA** as orange solid in 74% yield (two step yield).

**One-pot experimental procedure for the synthesis of methyl 1-(benzo[*b*]thiophene-2-carbonyl)-9*H*-pyrido[3,4-*b*]indole-3-carboxylate (6C).** To a stirred suspension of Cs_2_CO_3_ (0.182 g, 0.560 mmol) in dry THF (4 mL) in a 10 mL round-bottomed flask, methyl 1-acetyl-9-benzyl-9*H*-pyrido[3,4-*b*]indole-3-carboxylate (**5**, 0.10 g, 0.373 mmol) was added and the mixture was stirred for 10 min. Thereafter, 2-nitrobenzaldehyde (**C**, 0.062 g, 0.410 mmol) was added and the reaction mixture was stirred for additional 2 h at room temperature. After completion of the reaction (TLC), THF was evaporated under reduced pressure. Next, the crude nitrochalcone **5C** was re-dissolved in DMSO (1 mL) followed by the addition of sulfur powder (0.060 g, 1.86 mmol) and Et_3_N (0.260 mL, 1.86 mmol) at room temperature. The reaction mixture was stirred at 70 °C for 1 h. After completion of the reaction, the product was directly purified by silica gel column chromatography (hexane/EtOAc 60:40, v/v) to afford 0.056 g (39%) of **6C** as light brown solid (two step yield).

## Supporting Information

File 1 General information, experimental procedures, spectroscopic data, photophysical data, and copies of spectra.
