# Supplementary material for: Et3N/DMSO-supported one-pot synthesis of highly fluorescent β-carboline-linked benzothiophenones via sulfur insertion and estimation of the photophysical properties
Source: Beilstein J Org Chem. 2020 Jul 20;16:1740–53. doi: 10.3762/bjoc.16.146 (PMC7385337; doi:10.3762/bjoc.16.146)
Supplement: File 1 — General information, experimental procedures, spectroscopic data, photophysical data, and copies of spectra. [file Beilstein_J_Org_Chem-16-1740-s001.pdf]

BEILSTEIN JOURNAL OF ORGANIC CHEMISTRY

Supporting Information

for

Et_3N/DMSO-supported one-pot synthesis of highly fluorescent β-carboline-linked benzothiophenones via sulfur insertion and estimation of the photophysical properties

Dharmender Singh, Vipin Kumar and Virender Singh

Beilstein J. Org. Chem. **2020,** *16,* 1740–1753. doi:10.3762/bjoc.16.146

General information, experimental procedures, spectroscopic data, photophysical data, and copies of spectra

License and Terms: This is a supporting information file under the terms of the Creative Commons Attribution License (http://creativecommons.org/licenses/by/4.0). Please note that the reuse, redistribution and reproduction in particular requires that the authors and source are credited.
The license is subject to the *Beilstein Journal of Organic Chemistry* terms and conditions: (https://www.beilstein-journals.org/bjoc)

Table of contents

1. Synthesis of 1(3)-formyl-β-carboline derivatives S2

2. Experimental procedures and spectral data S3–S13

3. References S13

4. Photophysical properties and graphical data S14–S19

5. ^{1}H NMR and ^{13}C NMR spectra S20–S53

1. Synthesis of 1(3)-formyl-β-carboline derivatives

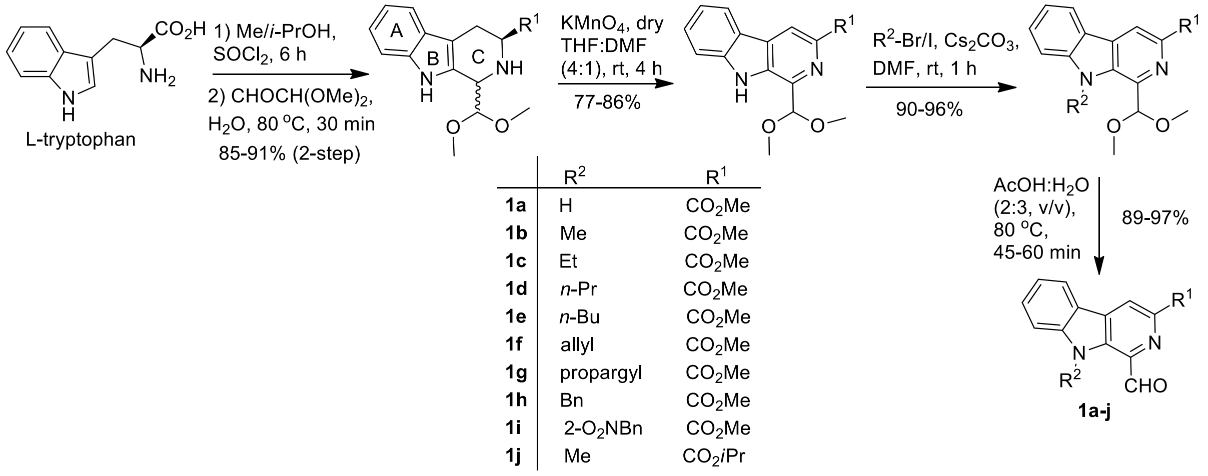

	R^2	R^1
**1a**	H	CO_2Me
**1b**	Me	CO_2Me
**1c**	Et	CO_2Me
**1d**	*n*-Pr	CO_2Me
**1e**	*n*-Bu	CO_2Me
**1f**	allyl	CO_2Me
**1g**	propargyl	CO_2Me
**1h**	Bn	CO_2Me
**1i**	2-O_2NBn	CO_2Me
**1j**	Me	CO_2iPr

Scheme S1: Synthesis of 1-formyl-9*H*-pyrido[3,4-*b*]indole derivatives [1]

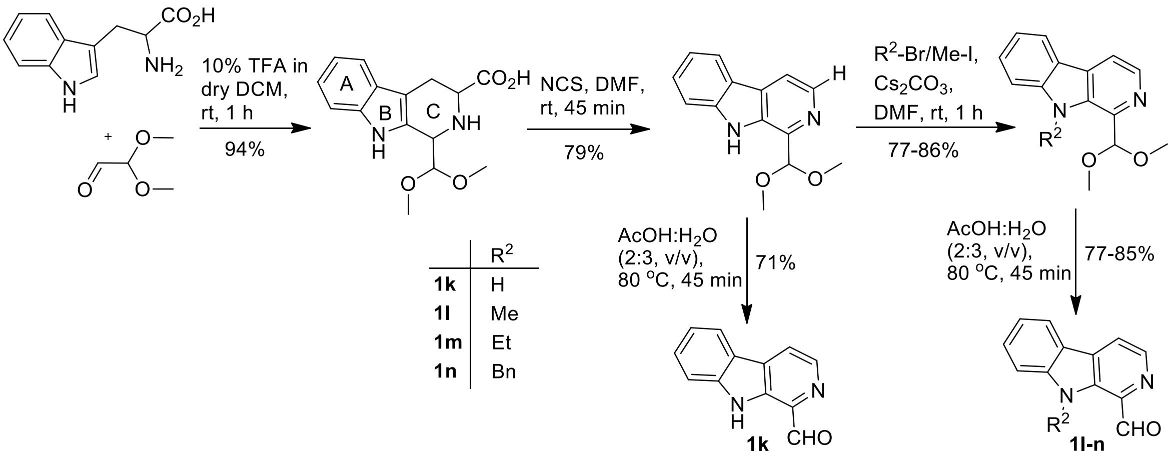

	R^2
**1k**	H
**1l**	Me
**1m**	Et
**1n**	Bn

Scheme S2: Synthesis of N-alkylated Kumujian C (**1k**-**n**) [1]

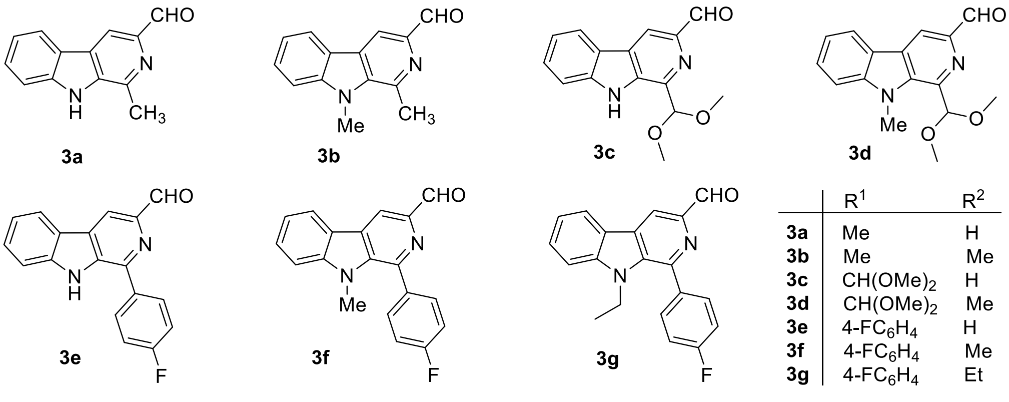

	R^1	R^2
**3a**	Me	H
**3b**	Me	Me
**3c**	$CH(OMe)_2$	H
**3d**	$CH(OMe)_2$	Me
**3e**	4-FC_6H_4	H
**3f**	4-FC_6H_4	Me
**3g**	4-FC_6H_4	Et

Scheme S3: Synthesis of 3-formyl-9*H*-pyrido[3,4-*b*]indoles [2]

2. Experimental procedures and spectral data

General methods: Chemicals and reagents were purchased from Sigma Aldrich, Acros, Spectrochem Ltd., and Avera Synthesis, and used without further purification. Commercially available anhydrous solvents (MeOH, iPrOH, diethyl ether, DMSO, and DMF) were used as received without further distillation. Thin layer chromatography (TLC) was performed on precoated aluminium plates (E. Merck; silica gel 60 PF254, 0.25 mm). Column chromatography was performed on silica gel (SRL; 60–120 mesh). Melting points were determined in open capillary tubes on a Precision Digital melting-point apparatus (LABCO) that contained silicon oil and are uncorrected. IR spectra were recorded on an Agilent FTIR spectrophotometer. ^{1}H and ^{13}C NMR spectra were recorded on an Avance III Bruker spectrometer at operating frequencies of 400 MHz, 500 MHz, 600 MHz (^{1}H), or 100 MHz, 125 MHz, or 150 MHz (^{13}C), as shown on the individual spectrum, using tetramethylsilane (TMS) as an internal standard. HRMS spectra were recorded on 6200 series TOF/6500 series QTOF B.05.01 (B5125). Elemental analysis was performed on a Carlo–Erba 108 or an Elementar Vario EL III microanalyzer. Room temperature varied between 25–40 °C. The multiplicity in the ^{1}H NMR spectra is as follows: s for singlet, d for doublet, t for triplet, q for quartet, dd for doublet of doublet, and m for multiplet.

Experimental procedure for the synthesis of β-carboline-based 2-nitrochalcone derivatives (1aA, 1bA, 1dA, and 1hA) as exemplified for compound 1bA. To a stirred solution of KOH (0.033 g, 0.587 mmol) in dry MeOH (4 mL), 2-nitroacetophenone (**A**, 0.080 mL, 0.587 mmol) was added at room temperature and the reaction mixture was stirred for 15 min. Thereafter, **1b** (0.15 g, 0.560 mmol) was added portionwise and the reaction mixture was allowed to stir for additional 1 h at room temperature. After completion of the reaction (as monitored by TLC), the yellow precipitate was filtered through a sintered funnel, washed twice with MeOH, and dried under vacuum to obtain 0.20 g (86%) of **1bA** as yellow solid.

(*E*)-Methyl 1-(3-(2-nitrophenyl)-3-oxoprop-1-en-1-yl)-9*H*-pyrido[3,4-*b*]indole-3-carboxylate (1aA). Yield: 76% (0.24 g from 0.20 g) as a yellow solid; m.p. 203-204 °C; R_f = 0.20 (hexane/EtOAc, 70:30, v/v); IR (neat): ν_{max} = 3319 (NH), 1713 (CO_2CH_3), 1585 (CO); ^{1}H NMR and ^{13}C NMR spectra could not be recorded due to solubility problem*;* MS (ES): *m/z* (%) = 402.1 (100) $[M+1]^+$; $C_{22}H_{15}N_3O_5$ (401.1012):calcd. for C 65.83, H 3.77, N 10.47; found C 65.89, H 3.78, N 10.49.

(*E*)-Methyl 9-methyl-1-(3-(2-nitrophenyl)-3-oxoprop-1-en-1-yl)-9*H*-pyrido[3,4-*b*]indole-3-carboxylate (1bA). Yield: 86% (0.20 g from 0.15 g) as a yellow solid; m.p. 207-208 °C; R_f = 0.30 (hexane/EtOAc, 70:30, v/v); IR (neat): ν_{max} = 1717 (CO_2CH_3), 1588 (CO); ^1H NMR (500 MHz, $CDCl_3$) δ = 4.03 (s, 3 H, NCH_3), 4.10 (s, 3 H, CO_2CH_3), 7.38 (t, *J* = 7.5 Hz, 1 H, ArH), 7.50 (d, *J* = 8.4 Hz, 1 H, ArH), 7.63 (d, *J* = 7.5 Hz, 1 H, ArH), 7.67 (d, *J* = 8.2 Hz, 2 H, ArH), 7.72 (d, *J* = 15.2 Hz, 1 H, ArH), 7.80 (t, *J* = 7.5 Hz, 1 H, ArH), 8.18 (t, *J* = 8.3 Hz, 2 H, ArH), 8.37 (d, *J* = 15.1 Hz, 1 H, ArH), 8.83 (s, 1 H, ArH) ppm; ^{13}C NMR (125 MHz, $CDCl_3$) δ = 33.6, 52.9, 110.1, 118.4, 121.2, 121.5, 122.0, 124.6, 129.0, 129.7, 130.5, 131.0, 131.5, 134.4, 136.3, 137.0, 137.6, 137.7, 139.3, 143.1, 146.8, 166.3, 191.9 ppm; MS (ES): *m/z* (%) = 416.1 (100) $[M+1]^+$; $C_{23}H_{17}N_3O_5$ (415.1168):calcd. for C 66.50, H 4.12, N 10.12; found C 66.61, H 4.15, N 10.18.

(*E*)-Methyl 1-(3-(2-nitrophenyl)-3-oxoprop-1-en-1-yl)-9-propyl-9*H*-pyrido[3,4-*b*]indole-3-carboxylate (1dA). Yield: 91% (0.205 g from 0.150 g) as a yellow solid; m.p. 159-161 °C; R_f = 0.35 (hexane/EtOAc, 70:30, v/v); IR (neat): ν_{max} = 1715 (CO_2CH_3), 1589 (CO); ^1H NMR (400 MHz, $CDCl_3$) δ = 0.92 (t, *J* = 7.4 Hz, 3 H, $NCH_2CH_2CH_3$), 1.80–1.89 (m, 2 H, $NCH_2CH_2CH_3$), 4.03 (s, 3 H, CO_2CH_3), 4.38 (t, *J* = 7.5 Hz, 2 H, $NCH_2CH_2CH_3$), 7.37 (t, *J* = 7.4 Hz, 1 H, ArH), 7.49 (d, *J* = 8.3 Hz, 1 H, ArH), 7.62–7.70 (m, 3 H, ArH), 7.77–7.81 (m, 2 H, ArH), 8.17–8.20 (m, 3 H, ArH), 8.85 (s, 1 H, ArH) ppm; ^{13}C NMR (100 MHz, $CDCl_3$) δ = 11.3, 23.3, 47.4, 52.7, 110.3, 118.4, 121.2, 121.4, 122.0, 124.6, 129.0, 129.6, 130.9, 131.0, 131.6, 134.3, 135.7, 136.8, 137.0, 137.6, 139.4, 142.6, 146.8, 166.3, 192.0 ppm; MS (ES): *m/z* (%) = 444.1 (100) $[M+1]^+$; $C_{25}H_{21}N_3O_5$ (443.1481):calcd. for C 67.71, H 4.77, N 9.48; found C 67.89, H 4.79, N 9.53.

(*E*)-Methyl 9-benzyl-1-(3-(2-nitrophenyl)-3-oxoprop-1-en-1-yl)-9*H*-pyrido[3,4-*b*]indole-3-carboxylate (1hA). Yield: 84% (0.18 g from 0.15 g) as a yellow solid; m.p. 179-181 °C; R_f = 0.37 (hexane/EtOAc, 70:30, v/v); IR (neat): ν_{max} = 1711 (CO_2CH_3), 1593 (CO); ^1H NMR (500 MHz, $CDCl_3$) δ = 4.05 (s, 3 H, CO_2CH_3), 5.58 (s, 2 H, NCH_2), 6.83 (d, *J* = 7.1 Hz, 2 H, ArH), 7.15–7.20 (m, 3 H, ArH), 7.34–7.42 (m, 3 H, ArH), 7.54 (t, *J* = 7.6 Hz, 1 H, ArH), 7.58–7.62 (m, 2 H, ArH), 7.71 (q, *J* = 15.3 Hz, 2 H, ArH), 8.03 (d, *J* = 8.1 Hz, 1 H, ArH), 8.22 (d, *J* = 7.7 Hz, 1 H, ArH), 8.89 (s, 1 H, ArH) ppm; ^{13}C NMR (125 MHz, $CDCl_3$) δ = 49.3, 52.9, 110.3, 118.4, 121.4, 121.9, 122.1, 124.5, 125.3, 128.0, 128.6, 129.3, 130.0, 130.5, 131.7, 132.0, 134.0, 135.7, 136.1, 136.4, 137.2, 138.2, 140.4, 142.9, 146.5, 166.3, 192.5 ppm; MS (ES): *m/z* (%) = 492.1 (100) $[M+1]^+$; $C_{29}H_{21}N_3O_5$ (491.1481):calcd. for C 70.87, H 4.31, N 8.55; found C 70.99, H 4.31, N 8.57.

Experimental procedure for the synthesis of β-carboline-substituted benzothiophenone derivatives (2aA, 2bA, 2dA, and 2hA) as exemplified for compound 2bA. In a 10 mL round-bottomed flask, **1bA** (0.20 g, 0.482 mmol), sulfur powder (0.077 g, 2.41 mmol), and Et_3N (0.336 mL, 2.41 mmol) in DMSO (1 mL) were added, and the mixture stirred at 70 °C for 18 min. After completion of the reaction (TLC), the crude mixture was directly purified by column chromatography on silica gel ($CHCl_3$/MeOH 95:5, v/v) to afford 0.15 g (78%) of **2bA** as orange solid.

One-pot experimental procedure for the synthesis of 2aA–nA, 2bB, 2hB, 4aA–gA, and **4eB as exemplified for compound 2bA.** In a 10 mL round-bottomed flask, to the stirred solution of KOH (0.033 g, 0.587 mmol) in dry MeOH (4 mL), 2-nitroacetophenone (0.080 mL, 0.587 mmol) was added at room temperature, and the reaction mixture was stirred for 15 min. Thereafter, **1b** (0.15 g, 0.560 mmol) was added portionwise and the reaction mixture was allowed to stirred for additional 1 h at room temperature. After completion of the reaction (TLC), excess MeOH was decanted and then evaporated under reduced pressure. Afterwards, the crude chalcone **1bA** was dissolved in DMSO (1.5 mL), followed by the sequential addition of sulfur powder (0.089 g, 2.80 mmol) and Et_3N (0.390 mL, 2.80 mmol) at room temperature. The reaction mixture was stirred at 70 °C for 18 min. After completion of the reaction (TLC), the mixture was directly purified by column chromatography on silica gel ($CHCl_3$/MeOH 95:5, v/v) to afford the analytically pure product **2bA** as orange solid in 74% yield (two step yield).

(*Z*)-Methyl 1-((3-oxobenzo[*b*]thiophen-2(3*H*)-ylidene)methyl)-9*H*-pyrido[3,4-*b*]indole-3-carboxylate (2aA). Yield: 35% (0.08 g from 0.15 g) as an orange solid; m.p. 264-266 °C; R_f = 0.50 (hexane/EtOAc, 70:30, v/v); IR (neat): ν_{max} = 3248 (NH), 1710 (CO_2CH_3), 1659 (C=O); ^1H NMR (400 MHz, $CDCl_3$) δ = ^1H NMR (400 MHz, DMSO-d_6) δ = 4.03 (s, 3 H, CO_2CH_3), 7.36–7.41 (m, 2 H, ArH), 7.67 (d, J = 8.0 Hz, 1 H, ArH), 7.69–7.75 (m, 2 H, ArH), 7.78 (d, J = 7.9 Hz, 1 H, ArH), 7.91 (d, J = 7.6 Hz, 1 H, ArH), 8.46 (d, J = 7.9 Hz, 1 H, ArH), 8.64 (s, 1 H, ArH), 8.95 (s, 1 H, ArH), 12.89 (s, 1 H, NH) ppm; ^{13}C NMR (100 MHz, $CDCl_3$) δ = 47.6, 121.6, 123.0, 124.7, 125.1, 126.4, 126.9, 129.8, 130.2, 131.0, 135.6, 136.3, 136.6, 137.0, 138.4, 141.5, 150.2, 166.0, 189.2 ppm; HRMS (ESI) m/z: calcd. for $C_{22}H_{14}N_2O_3S$ [M + Na^+]: 409.0623, found: 409.0673.

(*Z*)-Methyl 9-methyl-1-((3-oxobenzo[*b*]thiophen-2(3*H*)-ylidene)methyl)-9*H*-pyrido[3,4-*b*]indole-3-carboxylate (2bA). Yield: 74% (0.166 g from 0.150 g) as an orange solid; m.p. 274-276 °C; R_f = 0.55 (hexane/EtOAc, 70:30, v/v); IR (neat): ν_{max} = 1704 (CO_2CH_3), 1661 (C=O); ^{1}H NMR (400 MHz, $CDCl_3$) δ = 4.13 (s, 3 H, NCH_3), 4.31 (s, 3 H, CO_2CH_3), 7.27 (d, *J* = 6.6 Hz, 1 H, ArH), 7.38 (t, *J* = 7.2 Hz, 1 H, ArH), 7.56 (t, *J* = 6.9 Hz, 3 H, ArH), 7.67 (d, *J* = 7.6 Hz, 1 H, ArH), 7.92 (d, *J* = 7.9 Hz, 1 H, ArH), 8.18 (d, *J* = 7.7 Hz, 1 H, ArH), 8.69 (s, 1 H, ArH), 8.78 (s, 1 H, ArH) ppm; ^{13}C NMR (125 MHz, $CDCl_3$) δ = 33.6, 52.7, 110.2, 117.5, 121.4, 121.9, 124.4, 124.6, 125.5, 126.9, 129.5, 130.7, 131.0, 135.6, 136.7, 136.8, 137.1, 138.0, 142.8, 150.8, 166.3, 189.7 ppm; HRMS (ESI) m/z: calcd. for $C_{23}H_{16}N_2O_3S$ [M + Na^+]: 423.0779, found: 423.0725.

(*Z*)-Methyl 9-ethyl-1-((3-oxobenzo[*b*]thiophen-2(3*H*)-ylidene)methyl)-9*H*-pyrido[3,4-*b*]indole-3-carboxylate (2cA). Yield: 75% (0.11 g from 0.10 g) as an orange solid; m.p. 210-212 °C; R_f = 0.56 (hexane/EtOAc, 70:30, v/v); IR (neat): ν_{max} = 1709 (CO_2CH_3), 1672 (C=O); ^{1}H NMR (400 MHz, $CDCl_3$) δ = 1.68 (t, *J* = 7.2 Hz, 3 H, NCH_2CH_3), 4.14 (s, 3 H, CO_2CH_3), 4.76 (q, *J* = 7.2 Hz, 2 H, NCH_2CH_3), 7.26–7.30 (m, 1 H, ArH), 7.40 (t, *J* = 7.5 Hz, 1 H, ArH), 7.57 (dd, J_1 = 5.3 Hz, J_2 = 4.2 Hz, 2 H, ArH), 7.59 (d, *J* = 0.9 Hz, 1 H, ArH), 7.66–7.70 (m, 1 H, ArH), 7.93 (dt, J_1 = 7.7 Hz, J_2 = 1.0 Hz, 1 H, ArH), 8.21 (d, *J* = 7.8 Hz, 1 H, ArH), 8.59 (s, 1 H, ArH), 8.82 (s, 1 H, ArH) ppm; ^{13}C NMR (100 MHz, $CDCl_3$) δ = 14.8, 40.9, 52.8, 110.0, 117.6, 121.4, 121.5, 122.0, 124.4, 124.6, 125.6, 126.8, 129.5, 130.7, 131.2, 135.6, 136.2, 137.1, 137.2, 142.0, 150.8, 166.3, 189.7 ppm; HRMS (ESI) m/z: calcd. for $C_{24}H_{18}N_2O_3S$ [M + Na^+]: 437.0936, found: 437.0972.

(*Z*)-Methyl 1-((3-oxobenzo[*b*]thiophen-2(3*H*)-ylidene)methyl)-9-propyl-9*H*-pyrido[3,4-*b*]indole-3-carboxylate (2dA). Yield: 83% (0.18 g from 0.15 g) as an orange solid; m.p. 184-186 °C; R_f = 0.57 (hexane/EtOAc, 70:30, v/v); IR (neat): ν_{max} = 1710 (CO_2CH_3), 1662 (C=O); ^{1}H NMR (400 MHz, $CDCl_3$) δ = 1.19 (t, *J* = 7.3 Hz, 3 H, $NCH_2CH_2CH_3$), 2.04–2.10 (m, 2 H, $NCH_2CH_2CH_3$), 4.13 (s, 3 H, CO_2CH_3), 4.59 (t, *J* = 7.2 Hz, 2 H, $NCH_2CH_2CH_3$), 7.25–7.29 (m, 1 H, ArH), 7.36 (t, *J* = 7.2 Hz, 1 H, ArH), 7.55 (t, *J* = 5.6 Hz, 3 H, ArH), 7.63 (d, *J* = 7.2 Hz, 1 H, ArH), 7.90 (d, *J* = 7.4 Hz, 1 H, ArH), 8.16 (d, *J* = 7.1 Hz, 1 H, ArH), 8.51 (s, 1 H, ArH), 8.76 (s, 1 H, ArH) ppm; ^{13}C NMR (100 MHz, $CDCl_3$) δ = 11.4, 23.2, 47.4, 52.7, 110.2, 117.4, 121.3, 121.8, 124.3, 124.6, 125.4, 126.7, 129.4, 130.6, 131.1, 135.5, 136.7, 136.8, 137.1, 138.0, 142.8,

150.7, 166.2, 190.0 ppm; HRMS (ESI) m/z: calcd. for $C_{25}H_{20}N_2O_3S$ [M + H^+]: 429.1273, found: 429.1310.

(*Z*)-Methyl 9-butyl-1-((3-oxobenzo[*b*]thiophen-2(3*H*)-ylidene)methyl)-9*H*-pyrido[3,4-*b*]indole-3-carboxylate (2eA). Yield: 77% (0.165 g from 0.15 g) as an orange solid; m.p. 192-194 °C; R_f = 0.60 (hexane/EtOAc, 70:30, v/v); IR (neat): ν_{max} = 1711 (CO_2CH_3), 1670 (C=O); ^{1}H NMR (400 MHz, $CDCl_3$) δ = 1.07 (t, *J* = 7.4 Hz, 3 H, $NCH_2CH_2CH_2CH_3$), 1.59–1.68 (m, 2 H, $NCH_2CH_2CH_2CH_3$), 2.00–2.07 (m, 2 H, $NCH_2CH_2CH_2CH_3$), 4.15 (s, 3 H, CO_2CH_3), 4.69 (t, *J* = 7.7 Hz, 2 H, $NCH_2CH_2CH_2CH_3$), 7.26–7.30 (m, 1 H, ArH), 7.40 (t, *J* = 7.5 Hz, 1 H, ArH), 7.56–7.60 (m, 3 H, ArH), 7.66–7.77 (m, 1 H, ArH), 7.92–7.95 (m, 1 H, ArH), 8.21 (d, *J* = 7.8 Hz, 1 H, ArH), 8.60 (s, 1 H, ArH), 8.83 (s, 1 H, ArH) ppm; ^{13}C NMR (100 MHz, $CDCl_3$) δ = 14.0, 20.5, 31.9, 52.8, 57.6, 110.0, 110.3, 117.5, 121.4, 121.9, 124.5, 124.7, 125.6, 126.8, 129.5, 130.7, 135.6, 136.3, 137.1, 142.5, 150.8, 166.3, 184.0 ppm; HRMS (ESI) m/z: calcd. for $C_{26}H_{22}N_2O_3S$ [M + Na^+]: 465.1249, found: 465.1247.

(*Z*)-Methyl 9-allyl-1-((3-oxobenzo[*b*]thiophen-2(3*H*)-ylidene)methyl)-9*H*-pyrido[3,4-*b*]indole-3-carboxylate (2fA). Yield: 70% (0.152 g from 0.15 g) as a bright yellow solid; m.p. 183-185 °C; R_f = 0.57 (hexane/EtOAc, 70:30, v/v); IR (neat): ν_{max} = 1701 (CO_2CH_3), 1665 (C=O); ^{1}H NMR (500 MHz, $CDCl_3$) δ = 4.11 (s, 3 H, CO_2CH_3), 5.12 (d, *J* = 17.2 Hz, 1 H, =C*H*H), 5.26 (d, *J* = 1.7 Hz, 2 H, NCH_2), 5.33 (d, *J* = 10.6 Hz, 1 H, =CH*H*), 6.19–6.26 (m, 1 H, CH_2CH), 7.23–7.25 (m, 1 H, ArH), 7.38 (t, *J* = 7.4 Hz, 1 H, ArH), 7.48 (d, *J* = 8.3 Hz, 1 H, ArH), 7.54 (d, *J* = 3.2 Hz, 2 H, ArH), 7.64 (t, *J* = 7.7 Hz, 1 H, ArH), 7.87 (d, *J* = 7.6 Hz, 1 H, ArH), 8.17 (d, *J* = 7.8 Hz, 1 H, ArH), 8.46 (s, 1 H, ArH), 8.76 (s, 1 H, ArH) ppm; ^{13}C NMR (125 MHz, $CDCl_3$) δ = 48.2, 52.8, 110.2, 117.4, 118.2, 121.4, 121.6, 121.9, 124.4, 125.0, 125.5, 126.7, 129.6, 130.6, 131.1, 131.4, 135.5, 136.3, 136.7, 137.3, 137.5, 142.4, 150.7, 166.2, 189.6 ppm; HRMS (ESI) m/z: calcd. for $C_{25}H_{18}N_2O_3S$ [M + Na^+]: 449.0936, found: 449.0982.

(*Z*)-Methyl 1-((3-oxobenzo[*b*]thiophen-2(3*H*)-ylidene)methyl)-9-(prop-2-yn-1-yl)-9*H*-pyrido[3,4-*b*]indole-3-carboxylate (2gA). Yield: 45% (0.13 g from 0.20 g) as a dark yellow solid; m.p. 234-236 °C; R_f = 0.60 (hexane/EtOAc, 70:30, v/v); IR (neat): ν_{max} = 1704 (CO_2CH_3), 1673 (C=O); ^{1}H NMR (400 MHz, $CDCl_3$) δ = 2.50 (t, *J* = 2.4 Hz, 1 H, C≡CH), 4.14 (s, 3 H, CO_2CH_3), 5.39 (d, *J* = 2.4 Hz, 2 H, CH_2), 7.25–7.29 (m, 1 H, ArH), 7.42 (t, *J* = 7.5 Hz, 1 H, ArH), 7.57 (dd, J_1 = 4.6 Hz, J_2 = 1.0 Hz, 2 H, ArH), 7.60 (d, *J* = 8.3 Hz, 1 H, ArH), 7.68–7.72 (m, 1 H, ArH), 7.92 (d, *J* = 7.7 Hz, 1 H, ArH), 8.19 (d, *J* = 7.8 Hz, 1 H, ArH), 8.76 (s, 1 H, ArH), 8.78 (s, 1

H, ArH) ppm; ^{13}C NMR (100 MHz, $CDCl_3$) δ = 36.0, 52.9, 75.1, 76.8, 110.1, 117.4, 121.7, 122.0, 122.1, 124.4, 124.7, 125.5, 126.8, 129.8, 130.7, 131.6, 135.6, 136.8, 137.0, 137.3, 137.9, 141.9, 150.6, 166.1, 189.6 ppm; HRMS (ESI) m/z: calcd. for $C_{25}H_{16}N_2O_3S$ [M + Na^+]: 447.0779, found: 447.0788.

(*Z*)-Methyl 9-benzyl-1-((3-oxobenzo[b]thiophen-2(3*H*)-ylidene)methyl)-9*H*-pyrido[3,4-*b*]indole-3-carboxylate (2hA). Yield: 78% (0.162 g from 0.150 g) as an orange solid; m.p. 244-246 °C; R_f = 0.60 (hexane/EtOAc, 70:30, v/v); IR (neat): ν_{max} = 1712 (CO_2CH_3), 1675 (C=O); ^1H NMR (500 MHz, $CDCl_3$) δ = 4.13 (s, 3 H, CO_2CH_3), 5.92 (s, 2 H, NCH_2), 7.20 (d, *J* = 7.5 Hz, 2 H, ArH), 7.22–7.25 (m, 2 H, ArH), 7.31 (d, *J* = 7.7 Hz, 2 H, ArH), 7.43 (d, *J* = 7.5 Hz, 1 H, ArH), 7.52 (t, *J* = 5.6 Hz, 2 H, ArH), 7.58 (d, *J* = 8.3 Hz, 1 H, ArH), 7.68 (t, *J* = 7.6 Hz, 1 H, ArH), 7.85 (d, *J* = 7.6 Hz, 1 H, ArH), 8.26 (d, *J* = 7.8 Hz, 1 H, ArH), 8.39 (s, 1 H, ArH), 8.84 (s, 1 H, ArH) ppm; ^{13}C NMR (125 MHz, $CDCl_3$) δ = 49.4, 52.7, 110.3, 117.4, 121.4, 121.8, 122.0, 124.3, 124.9, 125.5, 126.1, 126.7, 128.3, 129.3, 129.8, 130.6, 131.4, 135.4, 135.6, 136.7, 136.8, 137.4, 137.5, 143.1, 150.6, 166.2, 189.4 ppm; HRMS (ESI) m/z: calcd. for $C_{29}H_{20}N_2O_3S$ [M + Na^+]: 499.1092, found: 499.1119.

(*Z*)-Methyl 9-(2-nitrobenzyl)-1-((3-oxobenzo[*b*]thiophen-2(3*H*)-ylidene)methyl)-9*H*-pyrido[3,4-*b*]indole-3-carboxylate (2iA). Yield: 67% (0.135 g from 0.15 g) as a dark brown solid; m.p. 238-240 °C; R_f = 0.55 (hexane/EtOAc, 70:30, v/v); IR (neat): ν_{max} = 1712 (CO_2CH_3), 1675 (C=O); ^1H NMR (500 MHz, $CDCl_3$) δ = 4.11 (s, 3 H, CO_2CH_3), 6.33 (s, 2 H, NCH_2), 6.64 (d, *J* = 7.7 Hz, 1 H, ArH), 7.17–7.20 (m, 1 H, ArH), 7.36 (t, *J* = 7.7 Hz, 1 H, ArH), 7.43–7.47 (m, 2 H, ArH), 7.49 (t, *J* = 5.8 Hz, 3 H, ArH), 7.65 (t, *J* = 7.5 Hz, 1 H, ArH), 7.77 (d, *J* = 7.6 Hz, 1 H, ArH), 7.85 (s, 1 H, ArH), 8.25 (d, *J* = 7.8 Hz, 1 H, ArH), 8.47 (dd, J_1 = 8.2 Hz, J_2 = 0.9 Hz, 1 H, ArH), 8.79 (s, 1 H, ArH) ppm; ^{13}C NMR (125 MHz, $CDCl_3$) δ = 48.1, 52.9, 110.0, 117.3, 121.6, 122.1, 122.2, 123.2, 124.3, 125.5, 126.5, 126.8, 127.3, 129.3, 130.0, 130.3, 131.4, 132.3, 134.8, 135.4, 136.6, 137.1, 137.2, 138.0, 142.5, 147.1, 150.1, 166.1, 188.8 ppm; HRMS (ESI) m/z: calcd. for $C_{29}H_{19}N_3O_5S$ [M + Na^+]: 544.0943, found: 544.0899.

(*Z*)-Isopropyl 9-methyl-1-((3-oxobenzo[b]thiophen-2(3*H*)-ylidene)methyl)-9*H*-pyrido[3,4-*b*]indole-3-carboxylate (2jA). Yield: 64% (0.14 g from 0.15 g) as an orange solid; m.p. 180-182 °C; R_f = 0.56 (hexane/EtOAc, 70:30, v/v); IR (neat): ν_{max} = 1703 (CO_2*i*-Pr), 1662 (C=O); ^1H NMR (500 MHz, $CDCl_3$) δ = 1.58 (d, *J* = 6.9 Hz, 6 H, $CO_2CH(CH_3)_2$), 4.14 (s, 1 H, $CO_2CH(CH_3)_2$), 4.32 (s, 3 H, NCH_3), 7.26–7.29 (m, 1 H, ArH), 7.39 (t, *J* = 7.5 Hz, 1 H, ArH), 7.51–7.58 (m, 3 H,

ArH), 7.68 (t, J = 7.7 Hz, 1 H, ArH), 7.92 (d, J = 7.6 Hz, 1 H, ArH), 8.20 (t, J = 7.6 Hz, 1 H, ArH), 8.70 (d, J = 3.9 Hz, 1 H, ArH), 8.79 (d, J = 4.7 Hz, 1 H, ArH) ppm; ^{13}C NMR (125 MHz, $CDCl_3$) δ = 22.5, 52.9, 69.5, 110.2, 117.2, 117.5, 121.4, 121.9, 124.3, 124.5, 125.5, 126.9, 129.5, 130.7, 131.1, 135.6, 136.4, 136.7, 136.8, 137.1, 142.7, 150.8, 166.2, 189.6 ppm; HRMS (ESI) m/z: calcd. for $C_{25}H_{20}N_2O_3S$ [M + Na^+]: 451.1092, found: 451.1136.

(*Z*)-2-((9-Methyl-9*H*-pyrido[3,4-*b*]indol-1-yl)methylene)benzo[*b*]thiophen-3(2*H*)-one (2lA). Yield: 77% (0.126 g from 0.100 g) as an orange solid; m.p. 248-250 °C; R_f = 0.75 (hexane/EtOAc, 70:30, v/v); ^{1}H NMR (400 MHz, $CDCl_3$) δ = 4.29 (s, 3 H, NCH_3), 7.27–7.35 (m, 2 H, ArH), 7.53 (dd, J_1 = 8.2 Hz, J_2 = 1.8 Hz, 2 H, ArH), 7.56 (dd, J_1 = 6.7 Hz, J_2 = 1.2 Hz, 1 H, ArH), 7.63–7.67 (m, 1 H, ArH), 7.93–7.95 (m, 1 H, ArH), 7.97 (d, J = 4.9 Hz, 1 H, ArH), 8.14 (d, J = 7.7 Hz, 1 H, ArH), 8.68 (d, J = 4.9 Hz, 1 H, ArH), 8.77 (s, 1 H, ArH) ppm; ^{13}C NMR (100 MHz, $CDCl_3$) δ = 33.6, 109.8, 115.1, 120.5, 121.0, 121.7, 124.2, 125.4, 125.9, 126.8, 129.1, 130.8, 130.9, 134.8, 135.4, 137.2, 138.6, 142.4, 150.7, 189.5 ppm; HRMS (ESI) m/z: calcd. for $C_{21}H_{14}N_2OS$ [M + H^+]: 343.0905, found: 343.0939.

(*Z*)-2-((9-Ethyl-9*H*-pyrido[3,4-*b*]indol-1-yl)methylene)benzo[*b*]thiophen-3(2*H*)-one (2mA). Yield: 74% (0.176 g from 0.15 g) as an orange solid; m.p. 256-258 °C; R_f = 0.80 (hexane/EtOAc, 70:30, v/v); IR (neat): ν_{max} = 1588 (C=O); ^{1}H NMR (400 MHz, $CDCl_3$) δ = 1.66 (t, J = 7.2 Hz, 3 H, NCH_2CH_3), 4.73 (q, J = 7.2 Hz, 2 H, NCH_2CH_3), 7.26–7.29 (m, 1 H, ArH), 7.33 (t, J = 7.5 Hz, 1 H, ArH), 7.54 (dd, J_1 = 12.2 Hz, J_2 = 8.0 Hz, 3 H, ArH), 7.64 (t, J = 7.7 Hz, 1 H, ArH), 7.93 (d, J = 7.6 Hz, 1 H, ArH), 7.97 (d, J = 4.9 Hz, 1 H, ArH), 8.15 (d, J = 7.8 Hz, 1 H, ArH), 8.64 (s, 1 H, ArH), 8.67 (d, J = 4.9 Hz, 1 H, ArH) ppm; ^{13}C NMR (100 MHz, $CDCl_3$) δ = 14.6, 40.7, 109.6, 115.2, 120.5, 121.2, 121.7, 124.2, 125.4, 125.9, 126.8, 129.1, 130.9, 131.0, 135.1, 135.4, 136.2, 136.4, 138.4, 141.7, 150.7, 189.6 ppm; HRMS (ESI) m/z: calcd. for $C_{22}H_{16}N_2OS$ [M + H^+]: 357.1062, found: 357.1071.

(*Z*)-2-((9-Benzyl-9*H*-pyrido[3,4-*b*]indol-1-yl)methylene)benzo[*b*]thiophen-3(2*H*)-one (2nA). Yield: 60% (0.132 g from 0.15 g) as an orange solid; m.p. 228-231 °C; R_f = 0.85 (hexane/EtOAc, 70:30, v/v); IR (neat): ν_{max} = 1672 (C=O); ^{1}H NMR (500 MHz, $CDCl_3$) δ = 5.85 (s, 2 H, CH_2Ph), 7.20–7.24 (m, 3 H, ArH), 7.27 (d, J = 8.7 Hz, 1 H, ArH), 7.33 (d, J = 7.5 Hz, 2 H, ArH), 7.37 (d, J = 7.4 Hz, 1 H, ArH), 7.46 (d, J = 7.7 Hz, 1 H, ArH), 7.51 (t, J = 8.0 Hz, 2 H, ArH), 7.62 (t, J = 7.6 Hz, 1 H, ArH), 7.85 (d, J = 7.6 Hz, 1 H, ArH), 7.98 (d, J = 5.5 Hz, 1 H, ArH), 8.18 (d, J = 7.8 Hz, 1 H, ArH), 8.40 (s, 1 H, ArH), 8.66 (d, J = 5.0 Hz, 1 H, ArH) ppm; ^{13}C NMR (125

MHz, $CDCl_3$) δ = 49.3, 109.9, 115.1, 120.9, 121.1, 121.8, 124.1, 125.3, 126.1, 126.3, 126.6, 128.1, 129.2, 129.4, 130.8, 131.1, 134.8, 135.2, 136.1, 136.5, 136.9, 138.8, 142.7, 150.5, 189.3 ppm; HRMS (ESI) m/z: calcd. for $C_{27}H_{18}N_2OS$ [M + Na^+]: 441.1038, found: 441.1066.

(*Z*)-Methyl 1-((5-chloro-3-oxobenzo[*b*]thiophen-2(3*H*)-ylidene)methyl)-9-methyl-9*H*-pyrido[3,4-*b*]indole-3-carboxylate (2bB). Yield: 65% (0.157 g from 0.15 g) as an orange solid; m.p. >250 °C; R_f = 0.75 (hexane/EtOAc, 80:20, v/v); ^{1}H NMR (400 MHz, $CDCl_3$) δ = 4.13 (s, 3 H, CO_2CH_3), 4.32 (s, 3 H, NCH_3), 7.23 (d, *J* = 8.2 Hz, 1 H, ArH), 7.39 (t, *J* = 7.4 Hz, 1 H, ArH), 7.56 (d, *J* = 8.1 Hz, 2 H, ArH), 7.67–7.71 (m, 1 H, ArH), 7.83 (d, *J* = 8.1 Hz, 1 H, ArH), 8.18 (d, *J* = 7.7 Hz, 1 H, ArH), 8.69 (s, 1 H, ArH), 8.80 (s, 1 H, ArH) ppm; ^{13}C NMR (100 MHz, $CDCl_3$) δ = 33.6, 52.9, 110.2, 117.7, 121.5, 121.9, 124.3, 125.2, 126.2, 127.6, 129.6, 131.2, 137.1, 142.1, 142.8, 152.5, 159.2, 162.7, 166.2, 188.2 ppm; HRMS (ESI) m/z: calcd. for $C_{23}H_{15}ClN_2O_3S$ [M + H^+]: 435.0570, found: 435.0570.

(*Z*)-methyl 9-benzyl-1-((5-chloro-3-oxobenzo[*b*]thiophen-2(3*H*)-ylidene)methyl)-9*H*-pyrido[3,4-*b*]indole-3-carboxylate (2hB). Yield: 68% (0.100 g from 0.100 g) as an orange solid; m.p. 232-234 °C; R_f = 0.85 (hexane/EtOAc, 80:20, v/v); IR (neat): ν_{max} = 1662 (C=O); ^{1}H NMR and ^{13}C NMR spectra could not be recorded due to solubility problem; HRMS (ESI) m/z: calcd. for $C_{29}H_{19}ClN_2O_3S$ [M + H^+]: 511.0883, found: 511.0869.

(*Z*)-2-((1-Methyl-9*H*-pyrido[3,4-*b*]indol-3-yl)methylene)benzo[*b*]thiophen-3(2*H*)-one (4aA). Yield: 51% (0.125 g from 0.150 g) as a brown solid; m.p. 223-225 °C; R_f = 0.50 (hexane/EtOAc, 60:40, v/v); IR (neat): ν_{max} = 3283 (NH), 1658 (C=O); ^{1}H NMR (400 MHz, DMSO-d_6) δ = 2.90 (s, 3 H, $ArCH_3$), 7.31 (dd, J_1 = 15.7 Hz, J_2 = 7.8 Hz, 2 H, ArH), 7.58 (t, *J* = 7.5 Hz, 1 H, ArH), 7.63–7.67 (m, 2 H, ArH), 7.74 (d, *J* = 7.8 Hz, 1 H, ArH), 7.82 (d, *J* = 7.6 Hz, 1 H, ArH), 8.05 (s, 1 H, ArH), 8.21 (d, *J* = 7.8 Hz, 1 H, ArH), 8.50 (s, 1 H, ArH), 12.03 (s, 1 H, NH) ppm; ^{13}C NMR (100 MHz, DMSO-d_6) δ = 20.4, 112.4, 119.3, 120.2, 121.1, 121.8, 124.3, 125.3, 125.9, 127.5, 128.4, 129.7, 130.3, 134.3, 135.3, 140.7, 142.6, 188.2 ppm; HRMS (ESI) m/z: calcd. for $C_{21}H_{14}N_2OS$ [M - H^+]: 341.0749, found: 341.0766.

(*Z*)-2-((1,9-Dimethyl-9*H*-pyrido[3,4-*b*]indol-3-yl)methylene)benzo[*b*]thiophen-3(2*H*)-one (4bA). Yield: 78% (0.124 g from 0.10 g) as a brown solid; m.p. 218-221 °C; R_f = 0.60 (hexane/EtOAc, 60:40, v/v); ^{1}H NMR (400 MHz, $CDCl_3$) δ = 3.25 (s, 3 H, $ArCH_3$), 4.22 (s, 3 H, NCH_3), 7.26 (d, *J* = 3.8 Hz, 1 H, ArH), 7.37 (t, *J* = 7.5 Hz, 1 H, ArH), 7.50 (d, *J* = 8.4 Hz, 1 H,

ArH), 7.57 (d, J = 3.5 Hz, 2 H, ArH), 7.65 (t, J = 7.5 Hz, 1 H, ArH), 7.95 (d, J = 7.6 Hz, 1 H, ArH), 8.10 (s, 1 H, ArH), 8.15 (s, 1 H, ArH), 8.17 (d, J = 7.8 Hz, 1 H, ArH) ppm; ^{13}C NMR (100 MHz, $CDCl_3$) δ = 23.9, 32.5, 109.9, 118.5, 120.6, 121.7, 124.0, 125.0, 126.7, 128.6, 129.3, 131.3, 131.8, 134.9, 135.4, 141.6, 142.2, 142.4, 150.4, 189.6 ppm; HRMS (ESI) m/z: calcd. for $C_{22}H_{16}N_2OS$ [M + H$^+$]: 357.1061, found: 357.1066.

(*Z*)-2-((1-(Dimethoxymethyl)-9*H*-pyrido[3,4-*b*]indol-3-yl)methylene)benzo[*b*]thiophen-3(2*H*)-one (4cA). Yield: 57% (0.085 g from 0.100 g) as a brown solid; m.p. 188-190 °C; R_f = 0.70 (hexane/EtOAc, 70:30, v/v); IR (neat): ν_{max} = 3404 (NH), 1662 (C=O); ^{1}H NMR (500 MHz, $CDCl_3$) δ = 3.62 (s, 6 H, CH(OC*H*$_3$)$_2$), 5.83 (s, 1 H, CH), 7.22–7.25 (m, 1 H, ArH), 7.31 (td, J_1 = 6.4 Hz, J_2 = 3.1 Hz, 1 H, ArH), 7.52–7.54 (m, 2 H, ArH), 7.56 (d, J = 6.2 Hz, 2 H, ArH), 7.91 (d, J = 7.7 Hz, 1 H, ArH), 8.10 (s, 1 H, ArH), 8.14 (d, J = 7.9 Hz, 1 H, ArH), 8.21 (s, 1 H, ArH), 9.41 (s, 1 H, ArH) ppm; ^{13}C NMR (125 MHz, $CDCl_3$) δ = 55.0, 106.8, 112.1, 120.0, 120.7, 121.2, 121.8, 124.0, 125.1, 126.7, 129.1, 130.7, 131.1, 131.6, 131.9, 132.9, 135.0, 140.6, 141.8, 150.2, 189.7 ppm; HRMS (ESI) m/z: calcd. for $C_{23}H_{18}N_2O_3S$ [M + Na$^+$]: 425.0936, found: 425.0922.

(*Z*)-2-((1-(Dimethoxymethyl)-9-methyl-9*H*-pyrido[3,4-*b*]indol-3-yl)methylene)benzo[*b*] thiophen-3(2*H*)-one (4dA). Yield: 68% (0.10 g from 0.10 g) as a brown solid; m.p. 182-184 °C; R_f = 0.75 (hexane/EtOAc, 70:30, v/v); IR (neat): ν_{max} = 1665 (C=O); ^{1}H NMR (500 MHz, $CDCl_3$) δ = 3.64 (s, 6 H, CH(OC*H*$_3$)$_2$), 4.23 (s, 3 H, NCH_3), 5.84 (s, 1 H, CH), 7.22–7.26 (m, 1 H, ArH), 7.34 (t, J = 7.4 Hz, 1 H, ArH), 7.51 (d, J = 8.3 Hz, 1 H, ArH), 7.55 (d, J = 3.2 Hz, 2 H, ArH), 7.63 (t, J = 7.6 Hz, 1 H, ArH), 7.93 (d, J = 7.6 Hz, 1 H, ArH), 8.11 (s, 1 H, ArH), 8.15 (d, J = 7.7 Hz, 1 H, ArH), 8.26 (s, 1 H, ArH) ppm; ^{13}C NMR (125 MHz, $CDCl_3$) δ = 33.7, 56.1, 110.3, 110.4, 120.1, 120.6, 121.2, 121.3, 124.0, 125.1, 126.7, 128.9, 131.2, 131.4, 131.6, 134.0, 134.9, 140.1, 141.3, 143.0, 150.2, 189.5 ppm; HRMS (ESI) m/z: calcd. for $C_{24}H_{20}N_2O_3S$ [M + Na$^+$]: 439.1092, found: 439.1129.

(*Z*)-2-((1-(4-Fluorophenyl)-9*H*-pyrido[3,4-*b*]indol-3-yl)methylene)benzo[*b*]thiophen-3(2*H*)-one (4eA). Yield: 69% (0.10 g from 0.10 g) as a brown solid; R_f = 0.60 (hexane/EtOAc, 80:20, v/v); ^{1}H NMR (400 MHz, $CDCl_3$) δ = 7.18 (dd, J_1 = 7.5 Hz, J_2 = 3.8 Hz, 1 H, ArH), 7.30 (d, J = 8.3 Hz, 2 H, ArH), 7.48 (d, J = 3.3 Hz, 3 H, ArH), 7.58 (d, J = 7.5 Hz, 2 H, ArH), 7.80 (d, J = 7.6 Hz, 1 H, ArH), 8.04 (s, 1 H, ArH), 8.08 (d, J = 8.2 Hz, 1 H, ArH), 8.17 (d, J = 10.3 Hz, 1 H, ArH), 8.20–8.26 (m, 2 H, ArH), 11.23 (s, 1 H, NH) ppm; ^{13}C NMR (100 MHz, $CDCl_3$) δ = 112.5, 115.7, 115.9, 119.2, 120.6, 121.5, 124.0, 124.9, 126.4, 128.6, 130.5, 130.7, 130.8, 131.9, 133.1,

134.3, 134.8, 141.4, 149.9, 189.4 ppm; HRMS (ESI) m/z: calcd. for $C_{26}H_{15}FN_2OS$ [M + H$^+$]: 423.0967, found: 423.0956.

(*Z*)-2-((1-(4-Fluorophenyl)-9-methyl-9*H*-pyrido[3,4-*b*]indol-3-yl)methylene)benzo[*b*]thiophen-3(2*H*)-one (4fA). Yield: 79% (0.113 g from 0.10 g) as an brown solid; R_f = 0.65 (hexane/EtOAc, 80:30, v/v); ^{1}H NMR (400 MHz, $CDCl_3$) δ = 3.58 (s, 3 H, NCH_3), 7.21–7.24 (m, 1 H, ArH), 7.33 (d, *J* = 8.7 Hz, 2 H, ArH), 7.39 (s, 1 H, ArH), 7.50 (dd, J_1 = 7.1 Hz, J_2 = 3.0 Hz, 3 H, ArH), 7.64–7.67 (m, 1 H, ArH), 7.84 (dd, J_1 = 8.7 Hz, J_2 = 5.4 Hz, 2 H, ArH), 7.90 (d, *J* = 7.6 Hz, 1 H, ArH), 8.14 (s, 1 H, ArH), 8.21 (d, *J* = 7.8 Hz, 1 H, ArH), 8.25 (s, 1 H, ArH) ppm; ^{13}C NMR (100 MHz, $CDCl_3$) δ = 33.7, 110.4, 115.4 (d, *J* = 21.0 Hz), 118.8, 121.0, 121.5, 121.8, 124.1, 125.1,126.7, 129.2, 131.0 (d, *J* = 6.0 Hz), 131.4, 131.8 (d, \ *J* = 8 Hz), 131.9, 132.0, 135.0, 142.2, 143.2, 143.5, 150.2, 163.5 (d, *J* = 251.2 Hz), 189.7 ppm; HRMS (ESI) m/z: calcd. for $C_{27}H_{17}FN_2OS$ [M + H$^+$]: 437.1124, found: 437.1109.

(*Z*)-2-((9-Ethyl-1-(4-fluorophenyl)-9*H*-pyrido[3,4-*b*]indol-3-yl)methylene)benzo[*b*]thiophen-3(2*H*)-one (4gA). Yield: 73% (0.103 g from 0.10 g) as a brown solid; R_f = 0.70 (hexane/EtOAc, 80:20, v/v); ^{1}H NMR (400 MHz, $CDCl_3$) δ = 1.02 (t, *J* = 7.1 Hz, 3 H, NCH_2CH_3), 4.13 (q, *J* = 7.1 Hz, 2 H, NCH_2CH_3), 7.21–7.25 (m, 1 H, ArH), 7.29–7.34 (m, 2 H, ArH), 7.37 (d, *J* = 7.1 Hz, 1 H, ArH), 7.47–7.53 (m, 3 H, ArH), 7.62–7.66 (m, 1 H, ArH), 7.79–7.83 (m, 2 H, ArH), 7.92 (d, *J* = 7.2 Hz, 1 H, ArH), 8.16 (s, 1 H, ArH), 8.23 (d, *J* = 7.7 Hz, 1 H, ArH), 8.28 (s, 1 H, ArH) ppm; ^{13}C NMR (100 MHz, $CDCl_3$) δ = 14.1, 39.7, 110.7, 115.5 (d, *J* = 21.0 Hz), 118.8, 120.9, 121.9, 124.1, 125.1, 126.7, 129.0, 131.1, 131.3 (d, *J* = 5.0 Hz), 131.8 (d, *J* = 44.0 Hz), 133.7, 134.8, 135.6, 142.3 (d, *J* = 31.0 Hz), 143.2, 150.3, 163.5 (d, *J* = 246.0 Hz), 189.7 ppm; HRMS (ESI) m/z: calcd. for $C_{28}H_{19}FN_2OS$ [M + H$^+$]: 451.1280, found: 451.1280.

(*Z*)-5-Chloro-2-((1-(4-fluorophenyl)-9*H*-pyrido[3,4-*b*]indol-3-yl)methylene)benzo[*b*]thiophen-3(2*H*)-one (4eB). Yield: 48% (0.076 g from 0.10 g) as an orange solid; m.p. 233-235 ^{o}C; R_f = 0.70 (hexane/EtOAc, 80:20, v/v); IR (neat): ν_{max} = 3383 (NH), 1669 (C=O); ^{1}H NMR and ^{13}C NMR spectra could not be recorded due to solubility problem; HRMS (ESI) m/z: calcd. for $C_{26}H_{14}ClFN_2OS$ [M + H$^+$]: 457.0578, found: 457.0565.

One-pot experimental procedure for the synthesis of 6C. In a 10 mL round-bottomed flask, to the stirred suspension of Cs_2CO_3 (0.182 g, 0.560 mmol) in dry THF (4 mL), **5** (0.100 g,

0.373 mmol) was added and the mixture stirred for 10 min. Thereafter, 2-nitrobenzaldehyde (**C**, 0.062 g, 0.410 mmol) was added and the reaction mixture was stirred for additional 2 h at room temperature. After completion of the reaction (TLC), THF was evaporated under reduced pressure. In a reaction vial, the crude chalcone **5C** was dissolved in DMSO (1 mL) followed by the sequential addition of sulfur powder (0.060 g, 1.86 mmol) and Et_3N (0.260 mL, 1.86 mmol) at room temperature. The reaction mixture was stirred at 70 °C for 1 h. After completion of the reaction, the mixture was directly purified by column chromatography on silica gel (hexane/EtOAc 60:40, v/v) to afford 0.056 g (39%) of **6C** as light-brown solid (two step yield).

Methyl 1-(benzo[*b*]thiophene-2-carbonyl)-9-benzyl-9*H*-pyrido[3,4-*b*]indole-3-carboxylate (6C). Yield: 39% (0.052 g from 0.10 g) as a yellow solid; m.p. 228-231 ^{o}C; R_f = 0.85 (hexane/EtOAc, 80:20, v/v); IR (neat): ν_{max} = 1658 (C=O); ^{1}H NMR (400 MHz, $CDCl_3$) δ = 4.04 (s, 3 H, CO_2CH_3), 5.75 (s, 2 H, NCH_2), 6.72–6.76 (m, 3 H, ArH), 6.81 (t, *J* = 7.2 Hz, 2 H, ArH), 7.34 (t, *J* = 7.8 Hz, 1 H, ArH), 7.43–7.48 (m, 2 H, ArH), 7.62 (d, *J* = 8.4 Hz, 1 H), 7.68–7.74 (m, 2 H, ArH), 7.75 (s, 1 H, ArH), 7.86 (dd, J_1 = 8.2 Hz, J_2 = 0.7 Hz, 1 H, ArH), 8.32 (d, *J* = 7.8 Hz, 1 H, ArH), 9.09 (s, 1 H, ArH) ppm; ^{13}C NMR (100 MHz, $CDCl_3$) δ = 48.6, 53.0, 110.8, 119.5, 121.3, 121.8, 121.9, 122.8, 124.7, 126.4, 126.7, 127.5, 127.6, 128.4, 130.0, 132.6, 135.2, 135.4, 135.7, 136.1, 139.1, 139.4, 142.4, 143.6, 143.7, 166.2, 186.5 ppm; HRMS (ESI) m/z: calcd. for $C_{29}H_{20}N_2O_3S$ [M + H^+]: 477.1273, found: 477.1290.

3. References:

[1] Singh, D.; Kumar, V.; Devi, N.; Malakar, C. C.; Shankar, R.; Singh, V. *Adv. Synth. Catal.*, **2017**, *359*, 1213–1226.

[2] Devi, N.; Singh, D.; Kaur, G.; Mor, S.; Putta, V. P. R. K.; Polina, S.; Malakar, C. C.; Singh, V. *New J. Chem.*, **2017**, *41*, 1082–1093.

4. Photophysical properties and graphical data

	UV-Vis	Fluorescence		Φ_F
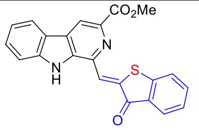 **2aA**	λ_{Ex} (nm)	λ_{Em} (nm)	Intensity	
	300.50	539.95	47.61	0.186
	481.00	540.88	38.78	0.295

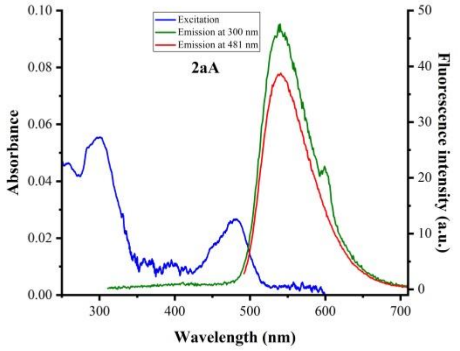

	UV-Vis	Fluorescence		Φ_F
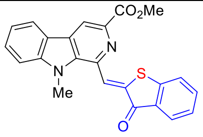 **2bA**	λ_{Ex} (nm)	λ_{Em} (nm)	Intensity	
	305.20	531.94	213.81	0.229
	487.78	536.86	178.31	0.342

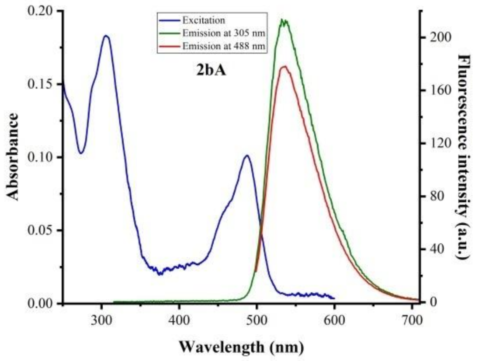

	UV-Vis	Fluorescence		Φ_F
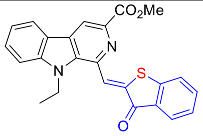 **2cA**	λ_{Ex} (nm)	λ_{Em} (nm)	Intensity	
	305.40	533.89	233.89	0.265
	488.18	532.83	193.36	0.347

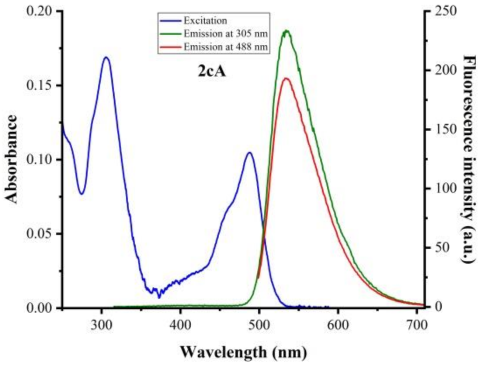

	UV-Vis	Fluorescence		Φ_F
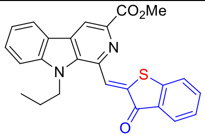 **2dA**	λ_{Ex} (nm)	λ_{Em} (nm)	Intensity	
	306.13	534.02	182.87	0.274
	487.50	534.02	143.78	0.383

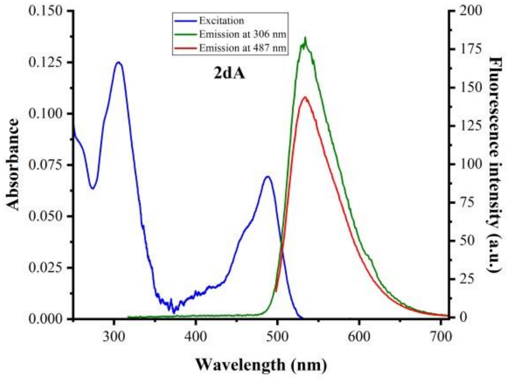

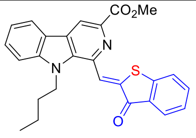 **2eA**	UV-Vis	Fluorescence		Φ_F
	λ_{Ex} (nm)	λ_{Em} (nm)	Intensity	
	304.93	531.94	197.94	0.279
	487.49	534.02	160.50	0.400

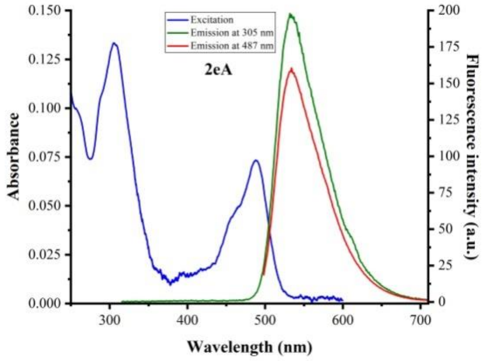

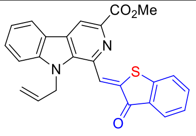 **2fA**	UV-Vis	Fluorescence		Φ_F
	λ_{Ex} (nm)	λ_{Em} (nm)	Intensity	
	303.41	530.89	144.88	0.256
	483.69	531.94	121.01	0.374

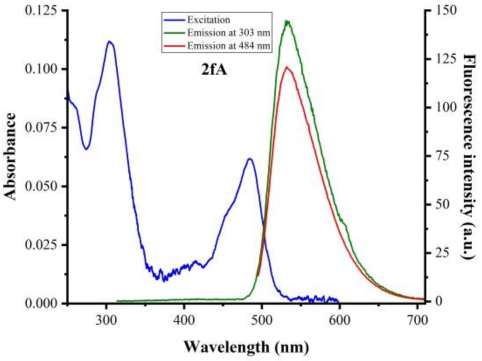

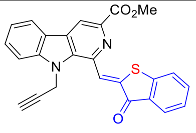 **2gA**	UV-Vis	Fluorescence		Φ_F
	λ_{Ex} (nm)	λ_{Em} (nm)	Intensity	
	303.61	534.02	121.25	0.221
	480.49	535.07	100.11	0.328

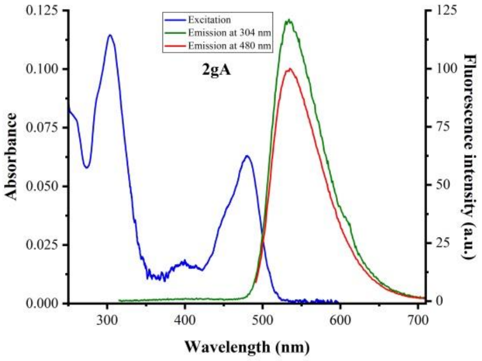

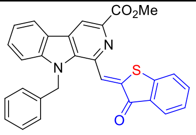 **2hA**	UV-Vis	Fluorescence		Φ_F
	λ_{Ex} (nm)	λ_{Em} (nm)	Intensity	
	303.21	532.98	39.57	0.302
	482.90	532.83	31.74	0.473

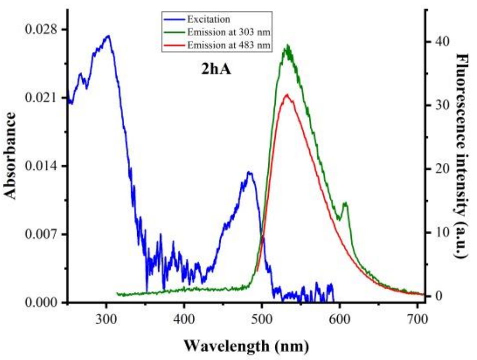

	UV-Vis	Fluorescence		Φ_F
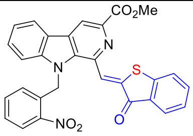 **2iA**	λ_{Ex} (nm)	λ_{Em} (nm)	Intensity	
	306.21	525.07	83.13	0.243
	480.08	524.91	67.85	0.337

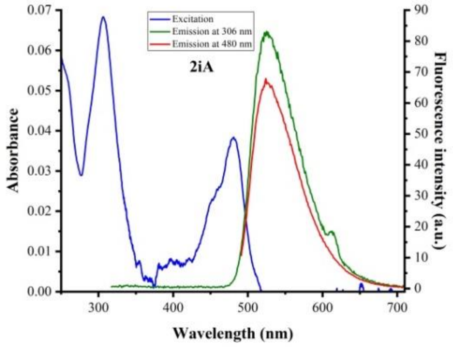

	UV-Vis	Fluorescence		Φ_F
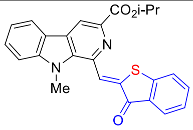 **2jA**	λ_{Ex} (nm)	λ_{Em} (nm)	Intensity	
	305.89	534.02	181.82	0.304
	487.51	534.98	148.43	0.378

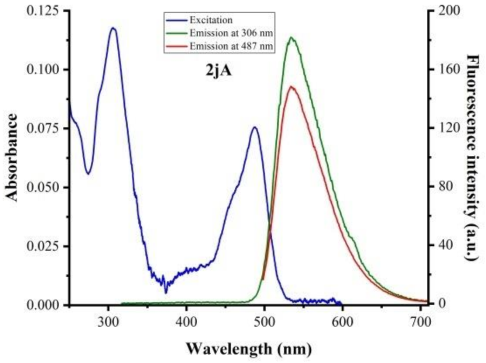

	UV-Vis	Fluorescence		Φ_F
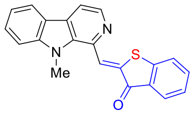 **2lA**	λ_{Ex} (nm)	λ_{Em} (nm)	Intensity	
	289.98	542.05	72.89	0.189
	492.53	541.08	99.43	0.253

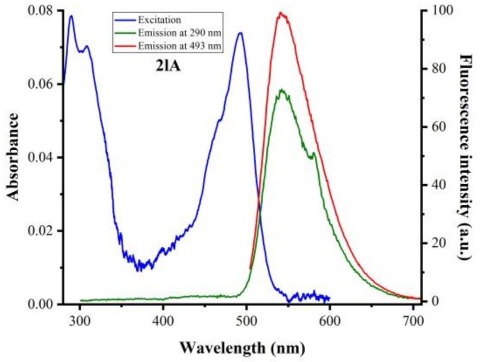

	UV-Vis	Fluorescence		Φ_F
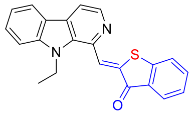 **2mA**	λ_{Ex} (nm)	λ_{Em} (nm)	Intensity	
	311.59	537.01	49.49	0.223
	491.81	538.95	66.73	0.366

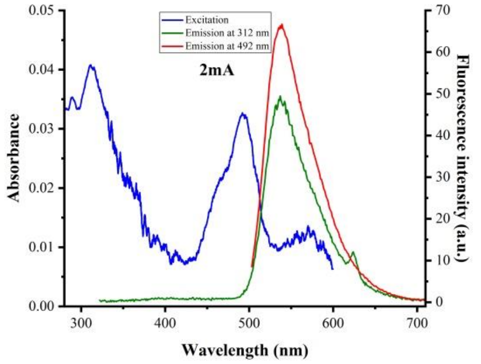

	UV-Vis	Fluorescence		Φ_F
	λ_{Ex} (nm)	λ_{Em} (nm)	Intensity	
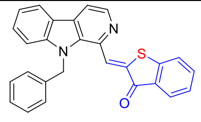 **2nA**	309.56	531.94	104.13	0.213
	487.74	531.79	126.29	0.349

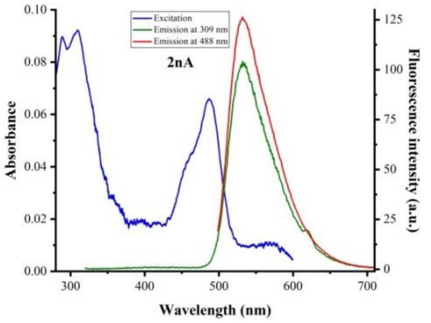

	UV-Vis	Fluorescence		Φ_F
	λ_{Ex} (nm)	λ_{Em} (nm)	Intensity	
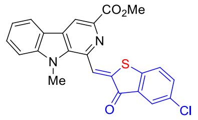 **2bB**	309.67	531.94	162.21	0.301
	486.62	535.97	127.26	0.378

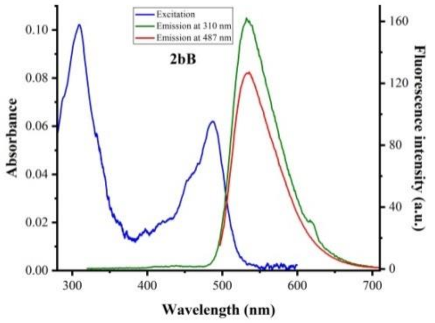

	UV-Vis	Fluorescence		Φ_F
	λ_{Ex} (nm)	λ_{Em} (nm)	Intensity	
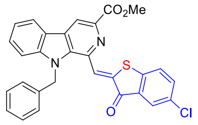 **2hB**	308.54	515.78	140.57	0.330
	482.71	521.54	116.95	0.258

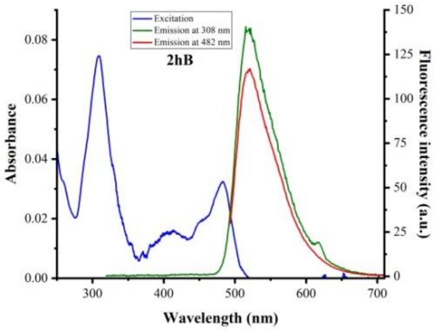

	UV-Vis	Fluorescence		Φ_F
	λ_{Ex} (nm)	λ_{Em} (nm)	Intensity	
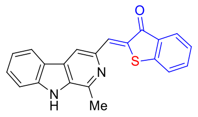 **4aA**	289.82	522.94	37.76	0.052
	463.50	512.83	39.76	0.095

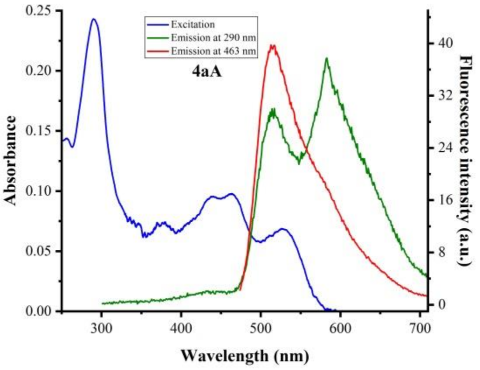

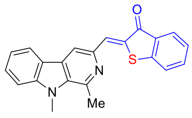 4bA	UV-Vis	Fluorescence		Φ_F
	λ_{Ex} (nm)	λ_{Em} (nm)	Intensity	
	289.62	514.64	69.79	0.167
	380.09	518.04	40.58	0.179
	470.77	517.02	89.14	0.219

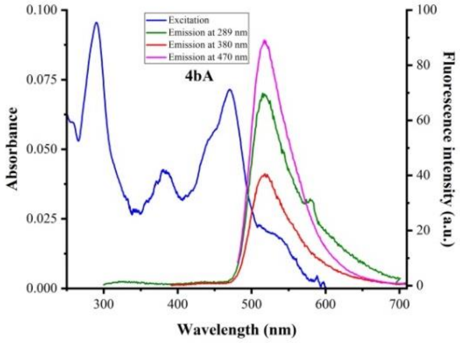

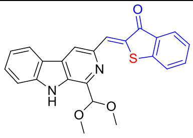 4cA	UV-Vis	Fluorescence		Φ_F
	λ_{Ex} (nm)	λ_{Em} (nm)	Intensity	
	289.17	514.91	38.82	0.079
	465.41	518.05	36.06	0.179

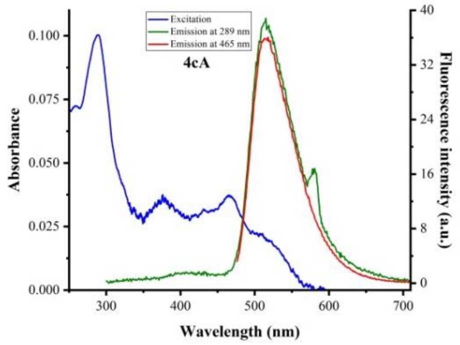

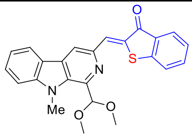 4dA	UV-Vis	Fluorescence		Φ_F
	λ_{Ex} (nm)	λ_{Em} (nm)	Intensity	
	293.41	518.05	119.83	0.160
	470.59	520.01	126.18	0.291

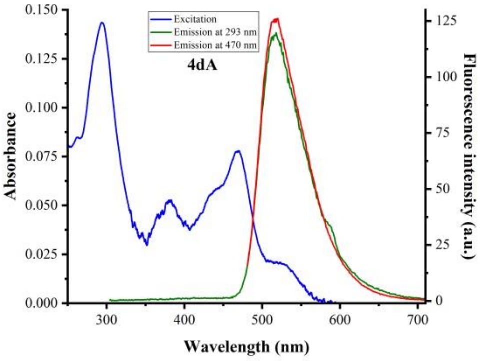

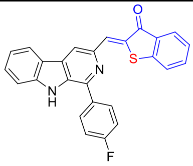 4eA	UV-Vis	Fluorescence		Φ_F
	λ_{Ex} (nm)	λ_{Em} (nm)	Intensity	
	282.56	521.94	107.64	0.111
	376.41	519.85	63.43	0.141
	466.89	521.04	99.98	0.196

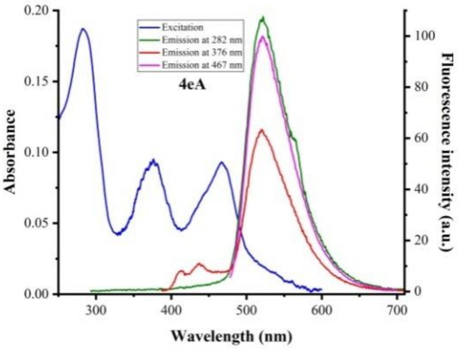

	UV-Vis	Fluorescence		Φ_F
	λ_{Ex} (nm)	λ_{Em} (nm)	Intensity	
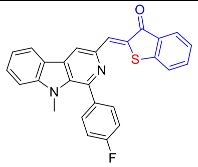 **4fA**	280.74	524.92	51.79	0.080
	383.74	520.89	29.12	0.141
	473.79	522.98	48.80	0.158

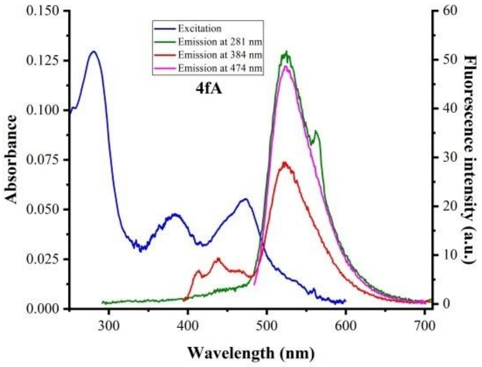

	UV-Vis	Fluorescence		Φ_F
	λ_{Ex} (nm)	λ_{Em} (nm)	Intensity	
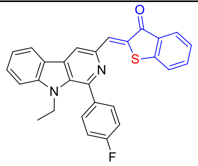 **4gA**	288.53	514.02	170.41	0.252
	380.11	517.01	86.20	0.284
	471.56	517.90	173.56	0.257

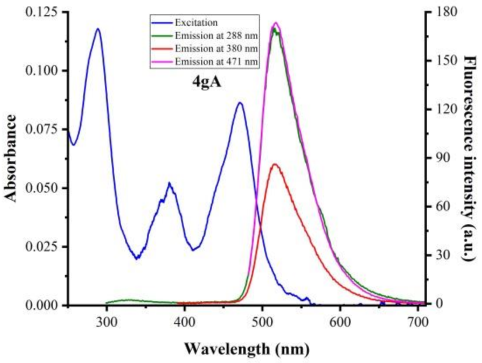

	UV-Vis	Fluorescence		Φ_F
	λ_{Ex} (nm)	λ_{Em} (nm)	Intensity	
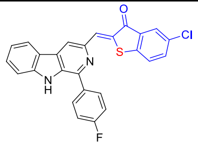 **4eB**	291.19	511.94	36.39	0.150
	464.27	508.04	29.47	0.225

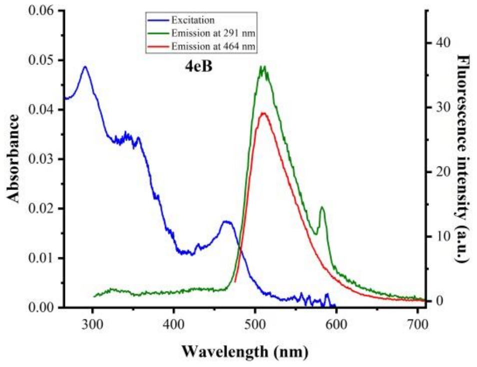

	UV-Vis	Fluorescence		Φ_F
	λ_{Ex} (nm)	λ_{Em} (nm)	Intensity	
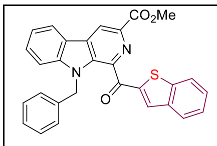 **6C**	263.14	525.96	13.60	0.034
	320.64	490.41	4.48	0.016

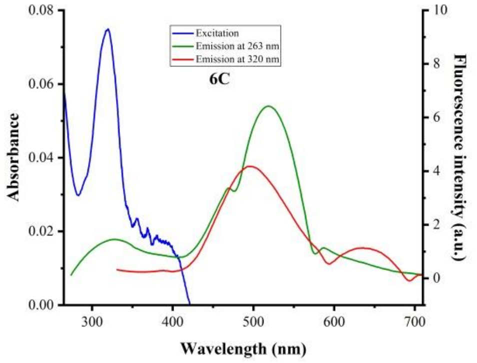

5. ^{1}H NMR and ^{13}C NMR spectra

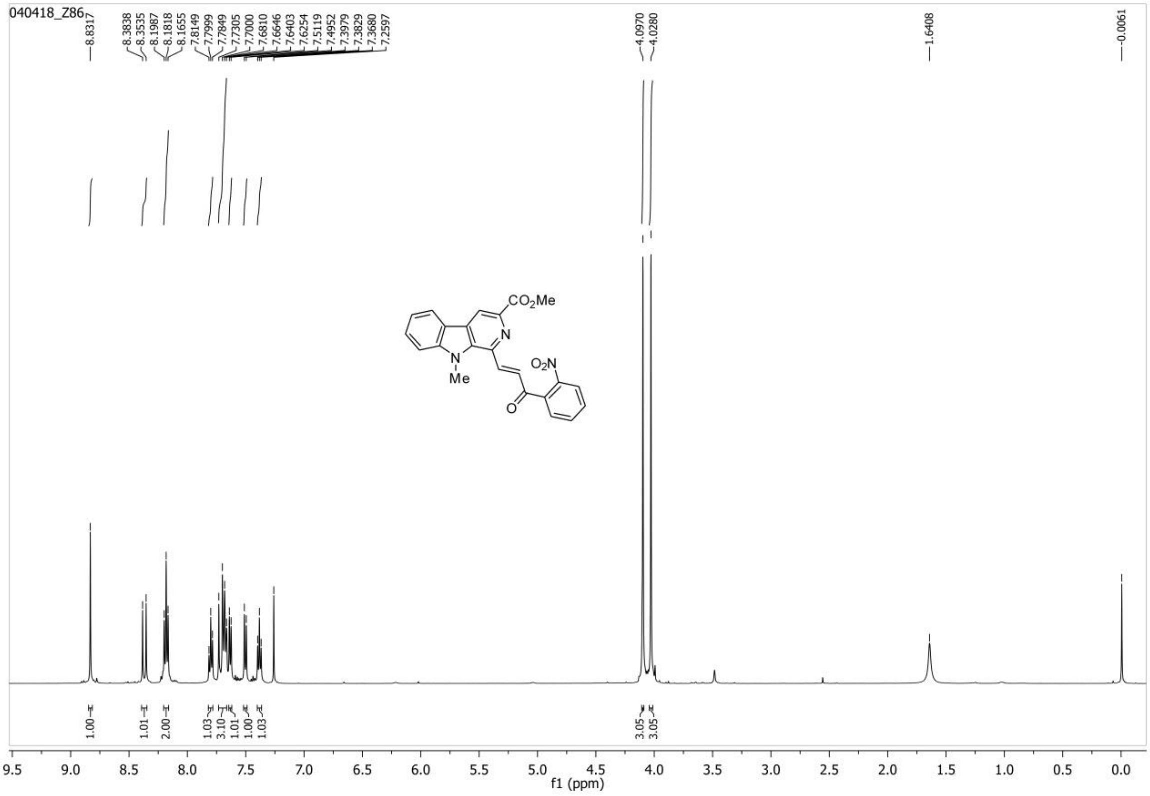

Figure S1. ^{1}H NMR spectrumof **1bA**.

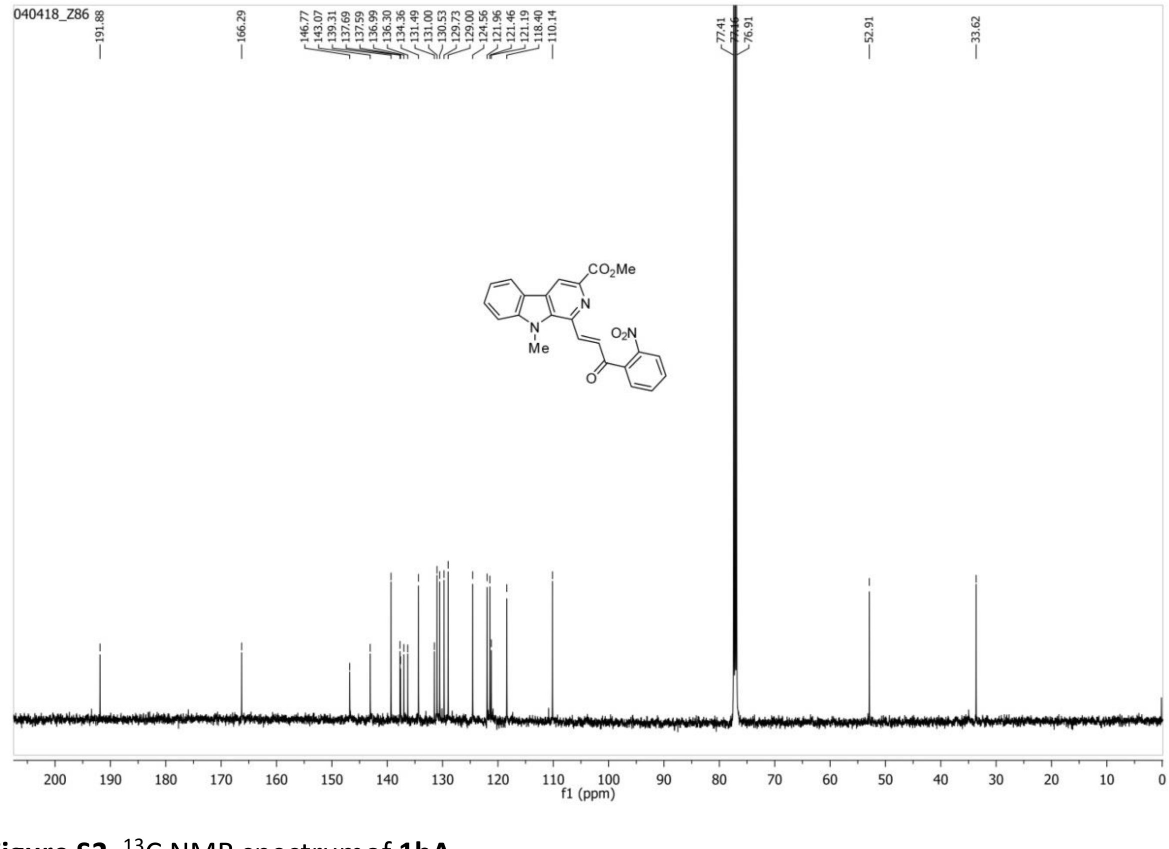

Figure S2. ^{13}C NMR spectrumof **1bA**.

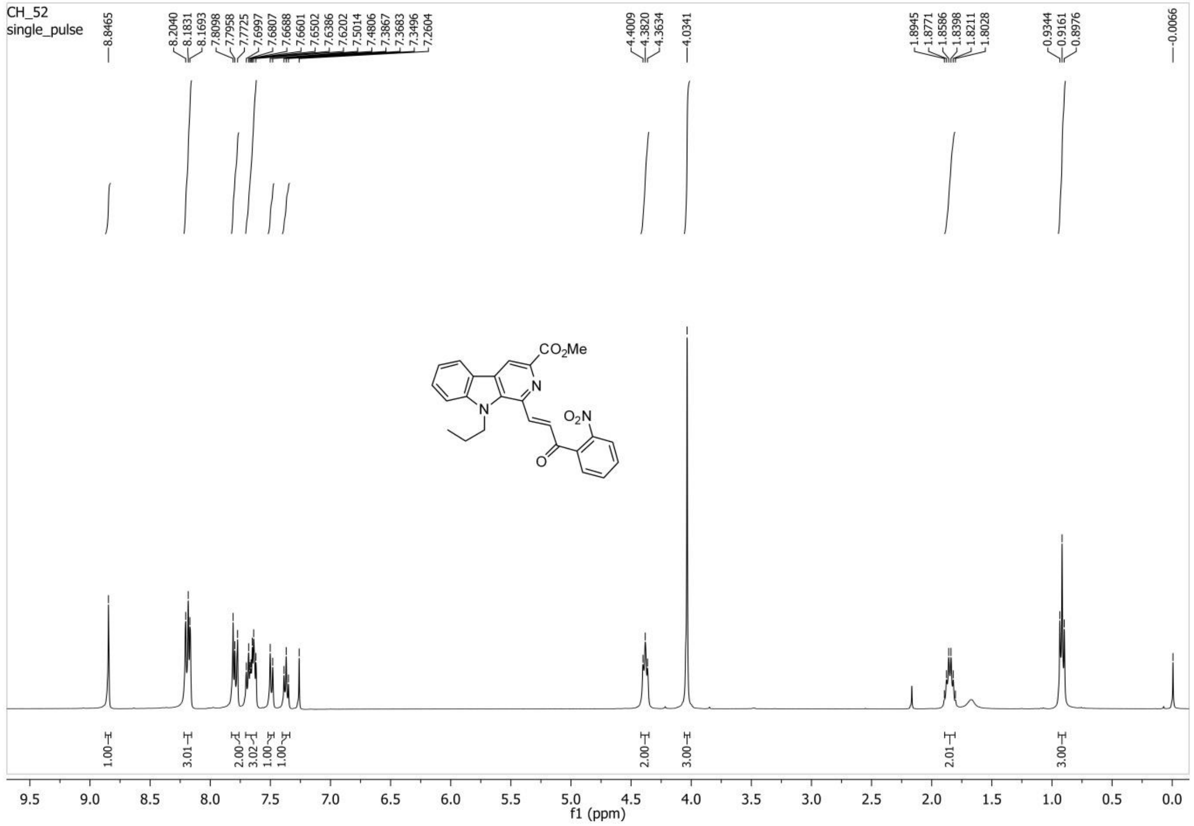

Figure S3. ^{1}H NMR spectrumof **1dA**.

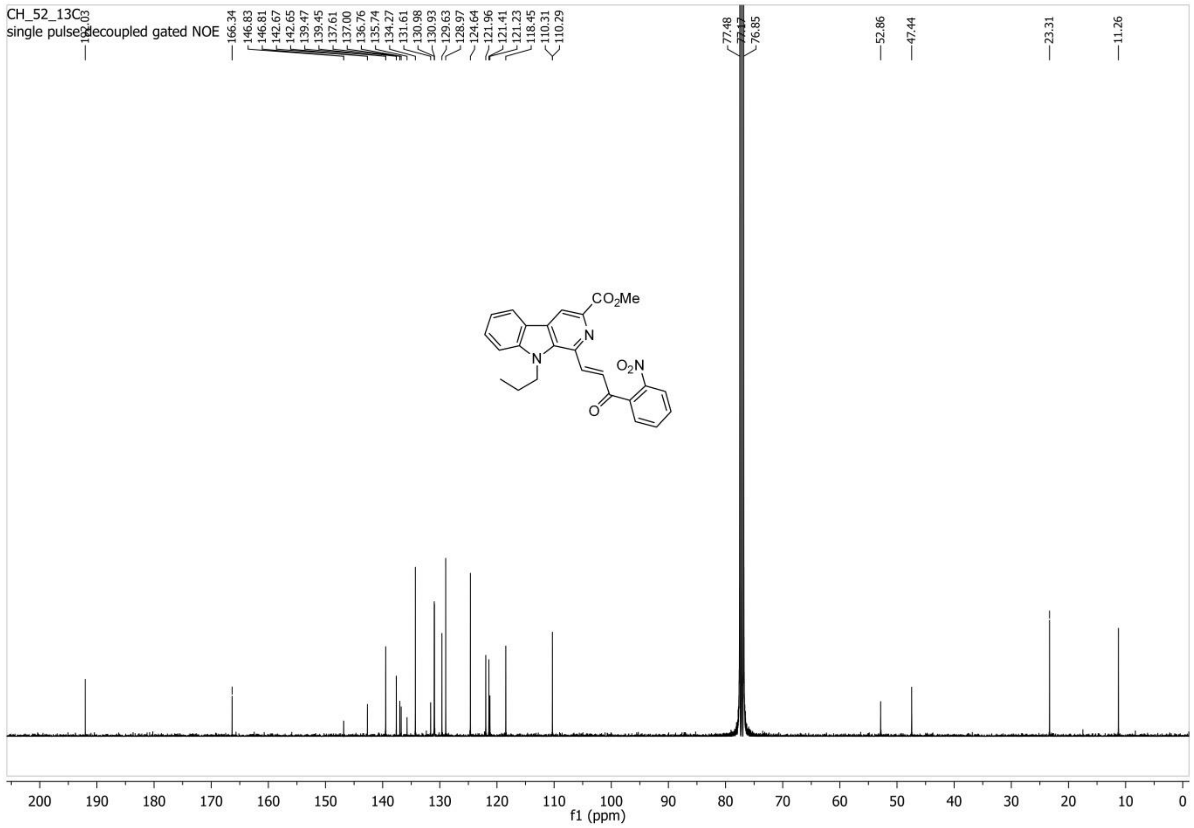

Figure S4. ^{13}C NMR spectrumof **1dA**.

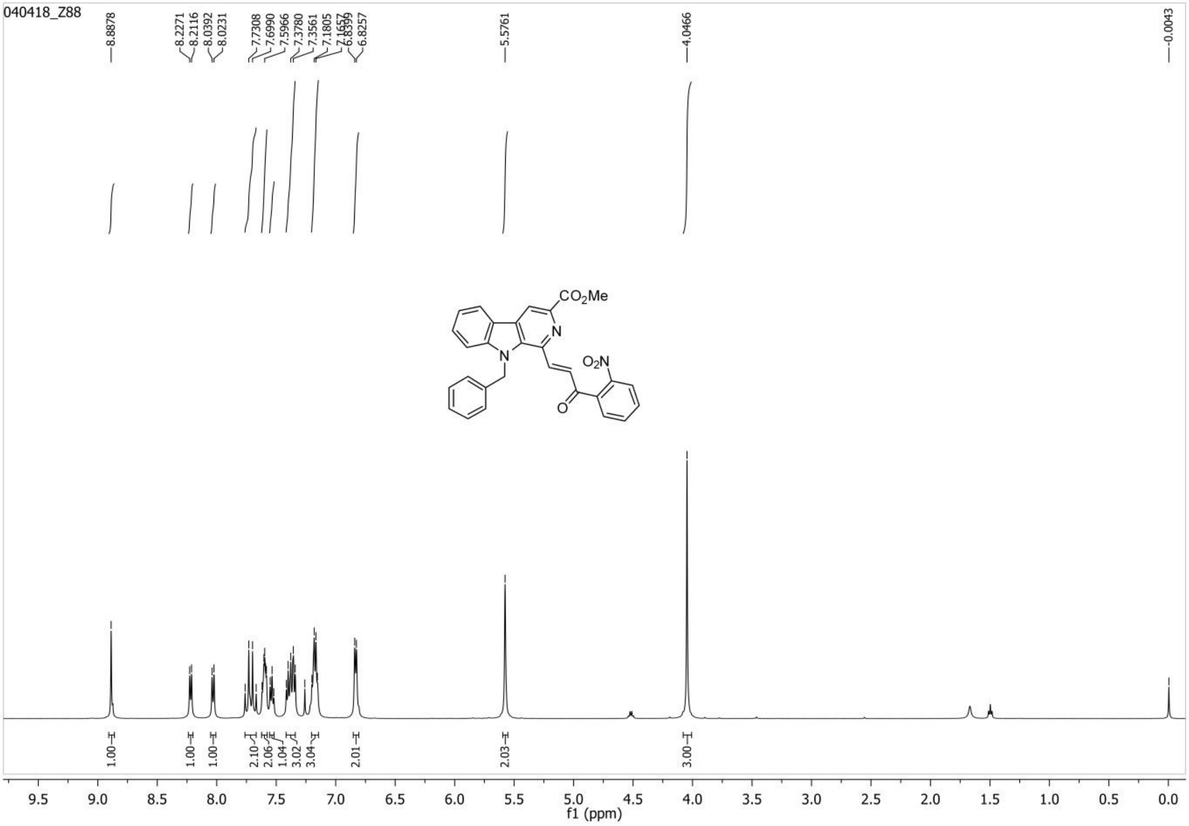

Figure S5. ^{1}H NMR spectrumof **1hA**.

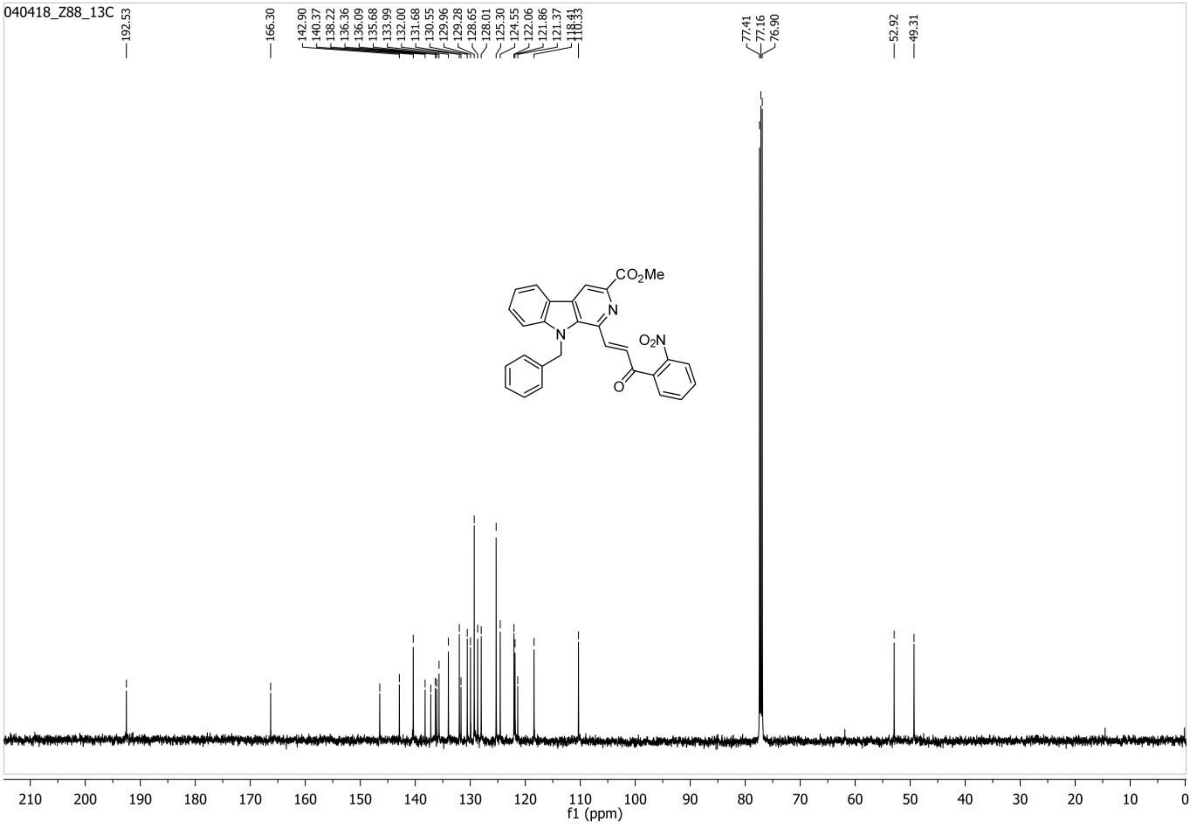

Figure S6. ^{13}C NMR spectrumof **1hA**.

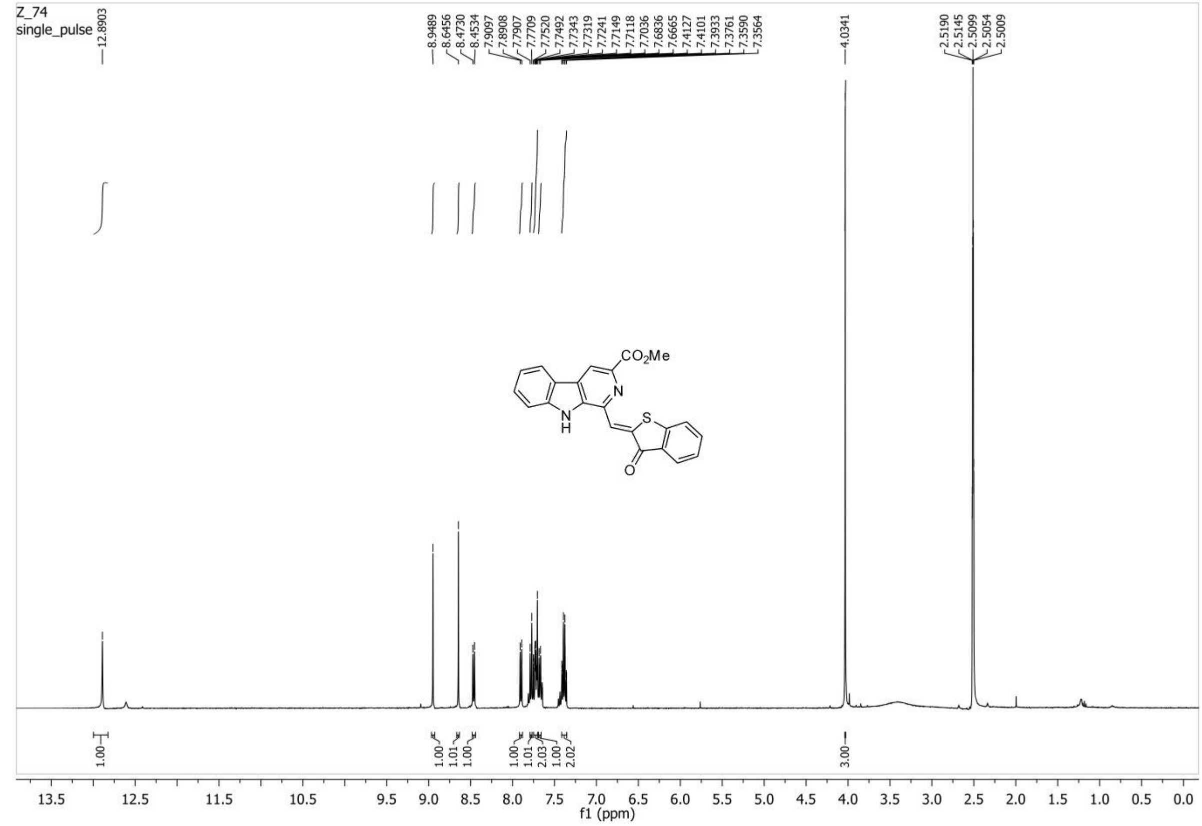

Figure S7. ^{1}H NMR spectrumof **2aA**.

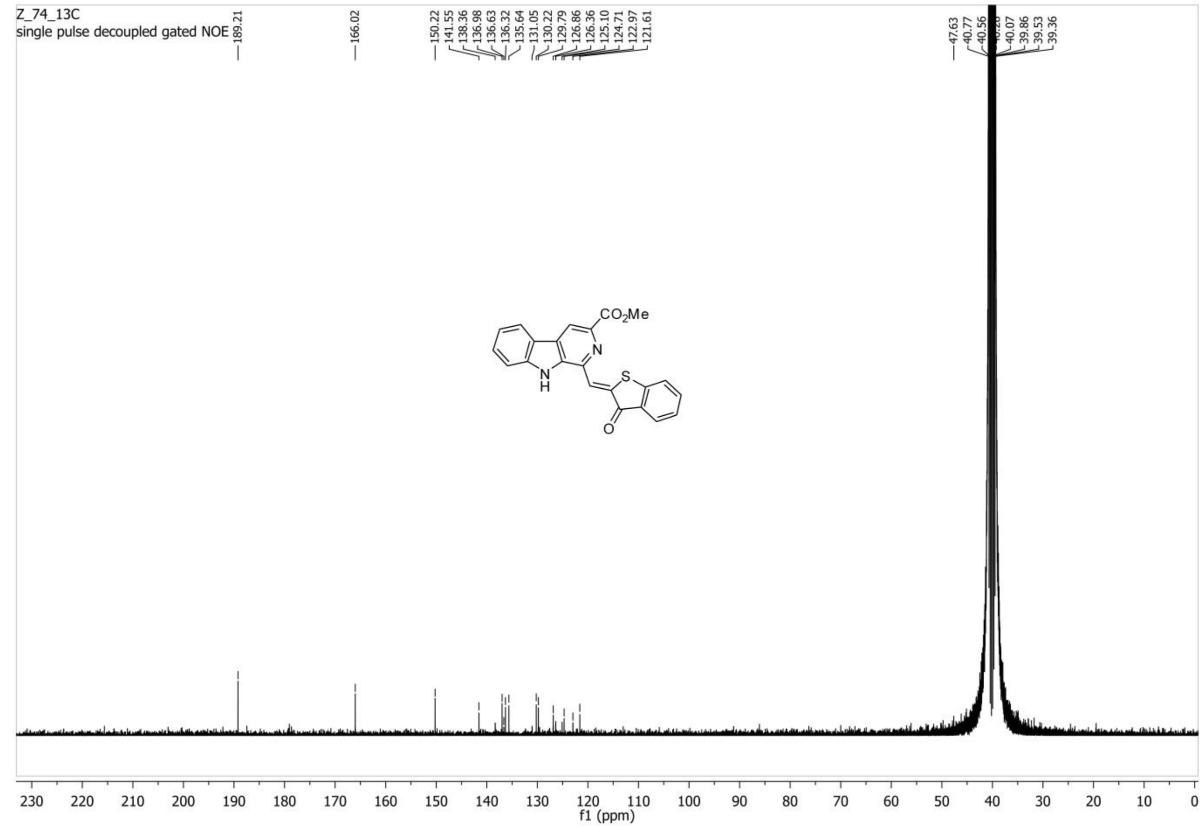

Figure S8. ^{13}C NMR spectrumof **2aA**.

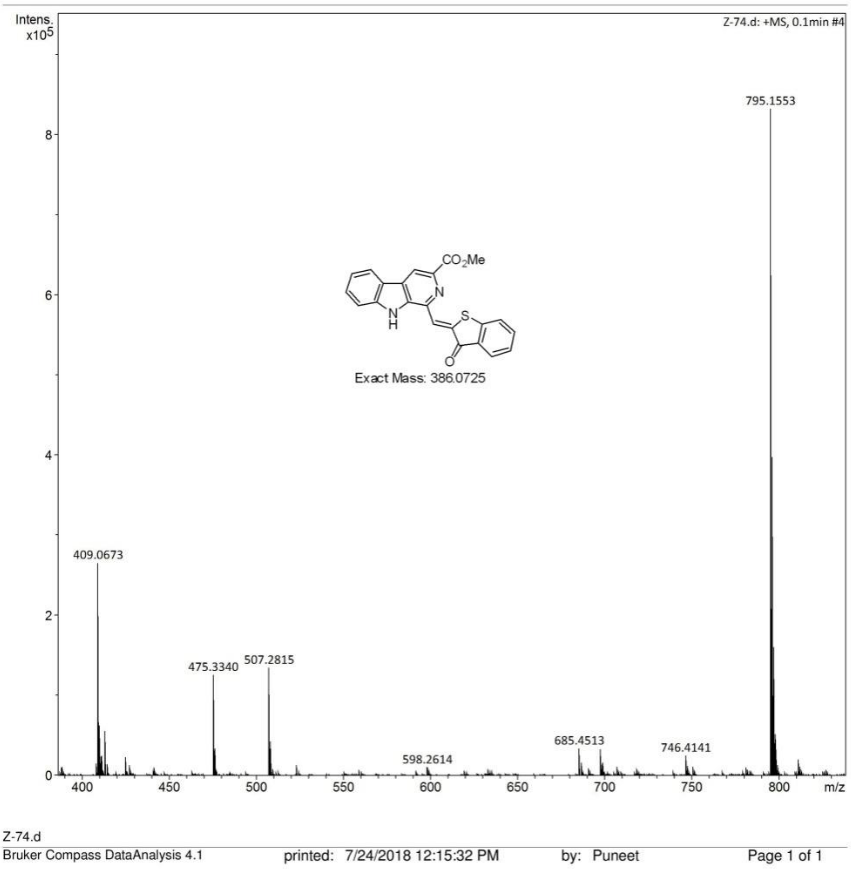

Figure S9. HRMS spectrum of **2aA**.

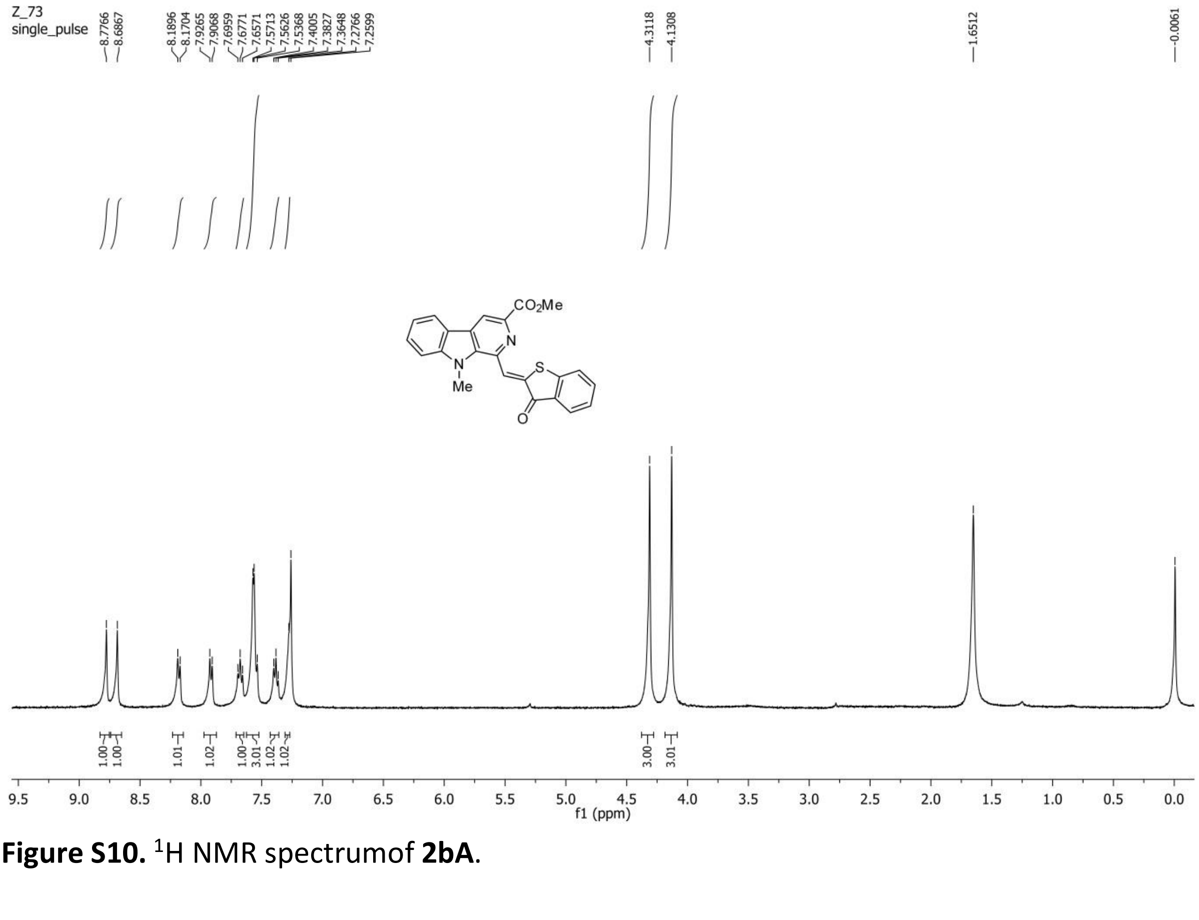

Figure S10. ^{1}H NMR spectrumof **2bA**.

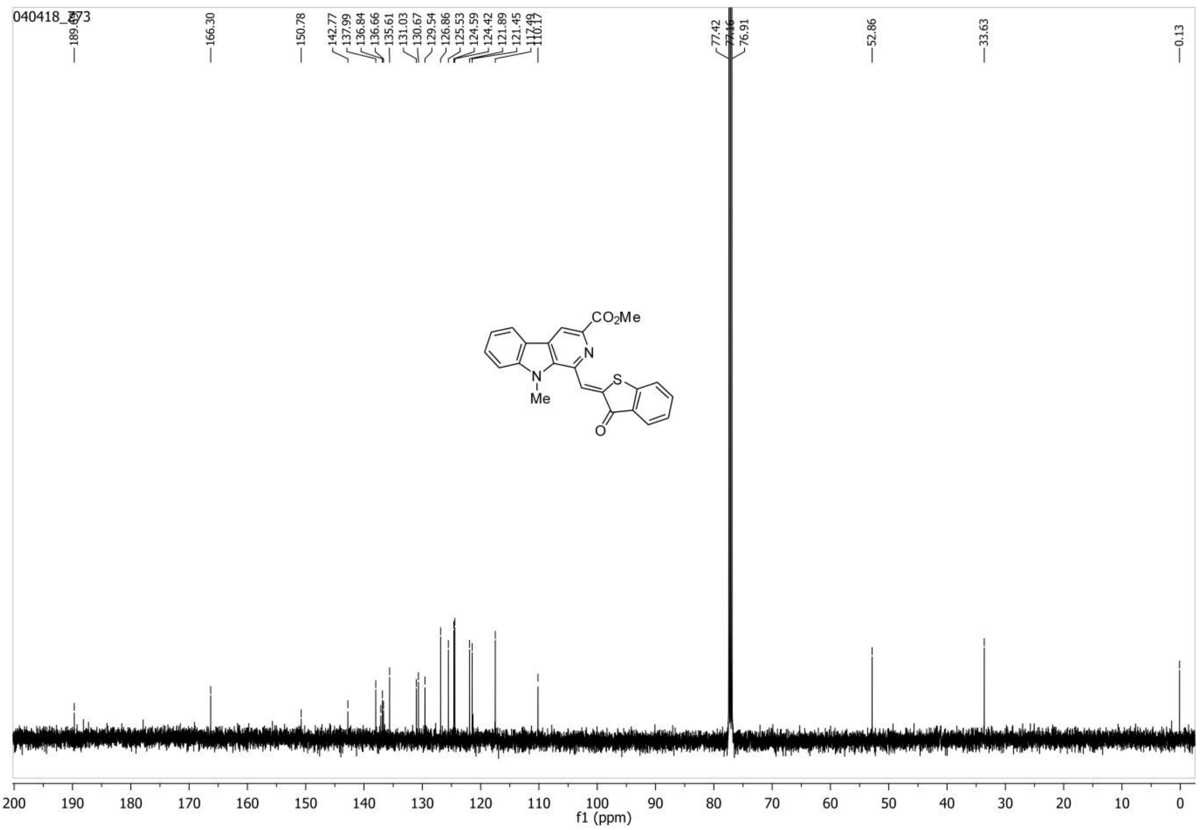

Figure S11. ^{13}C NMR spectrumof **2bA**.

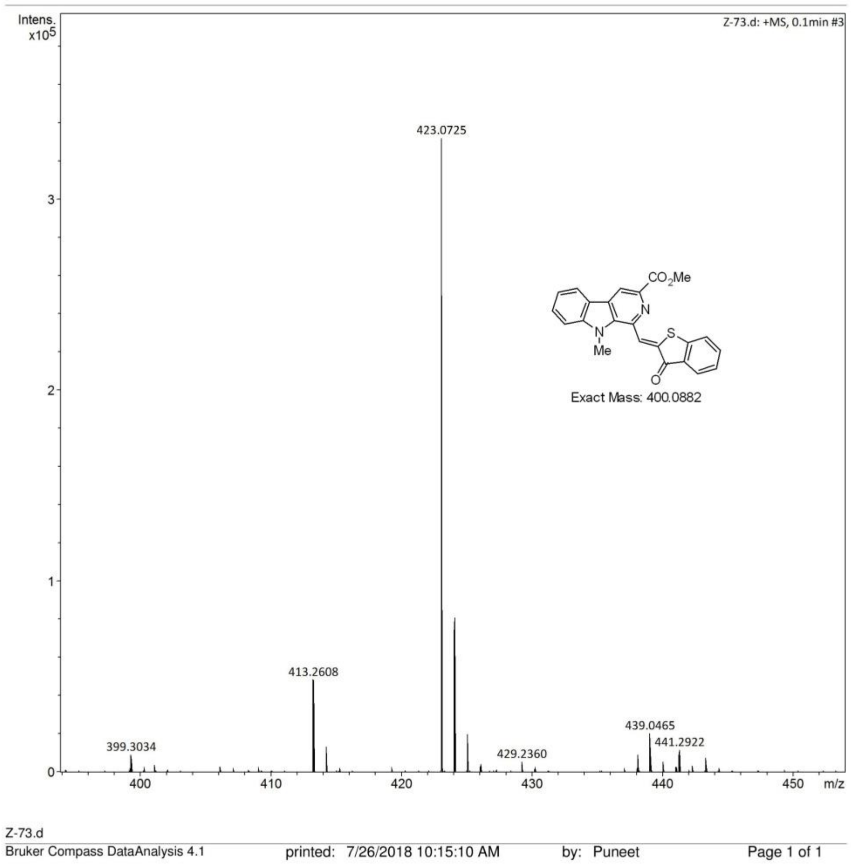

Figure S12. HRMS spectrum of **2bA**.

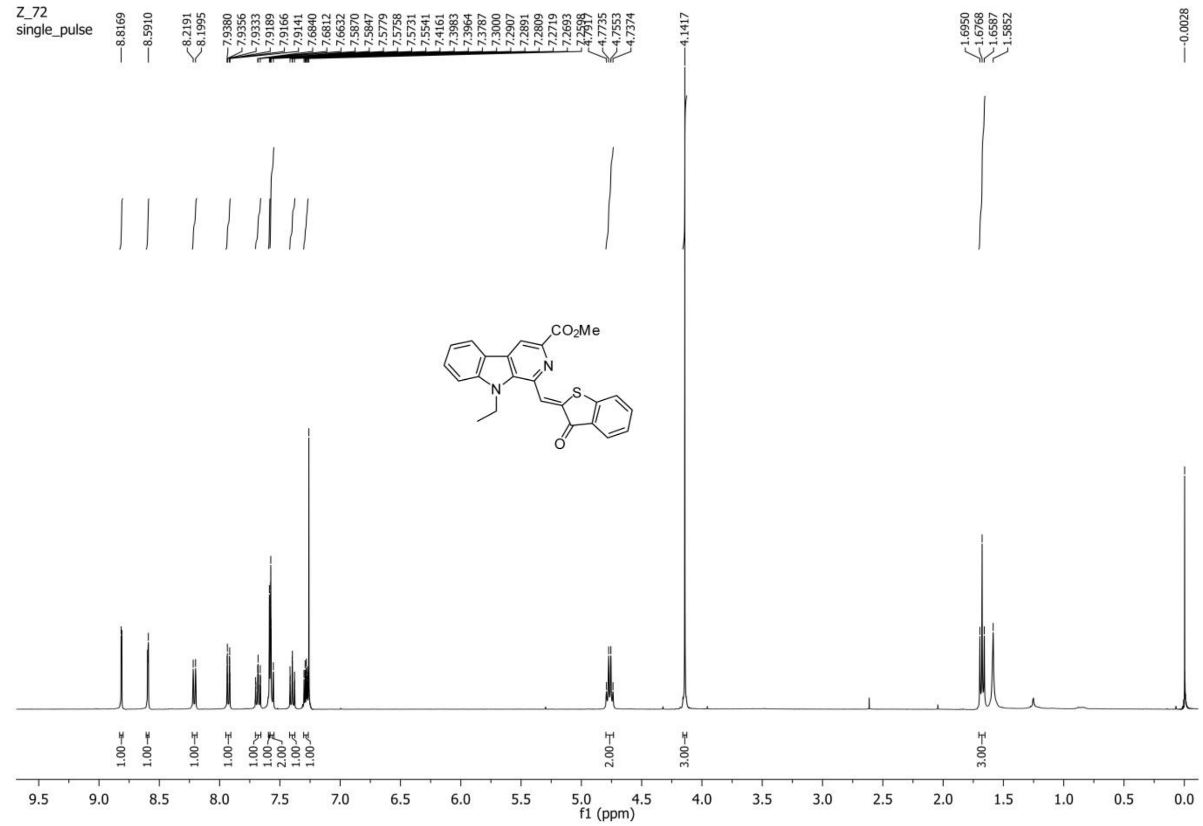

Figure S13. ^{1}H NMR spectrumof **2cA**.

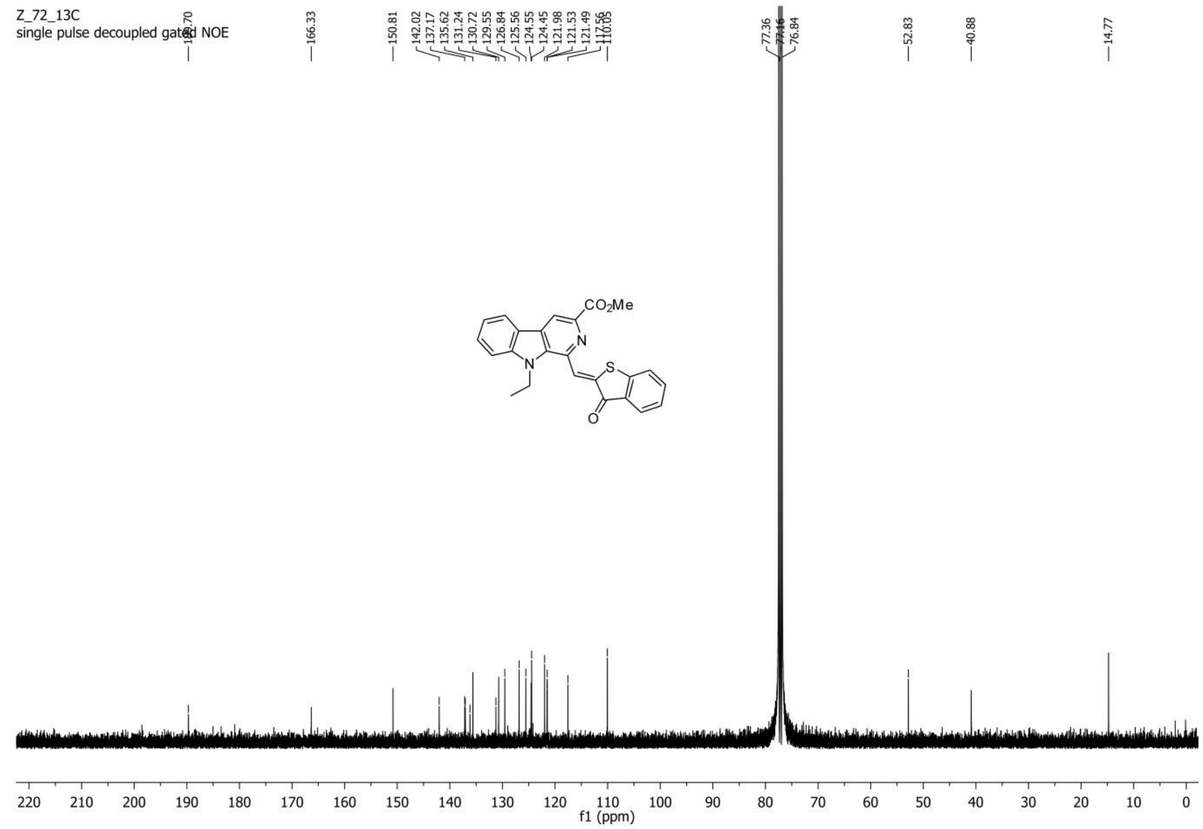

Figure S14. ^{13}C NMR spectrumof **2cA**.

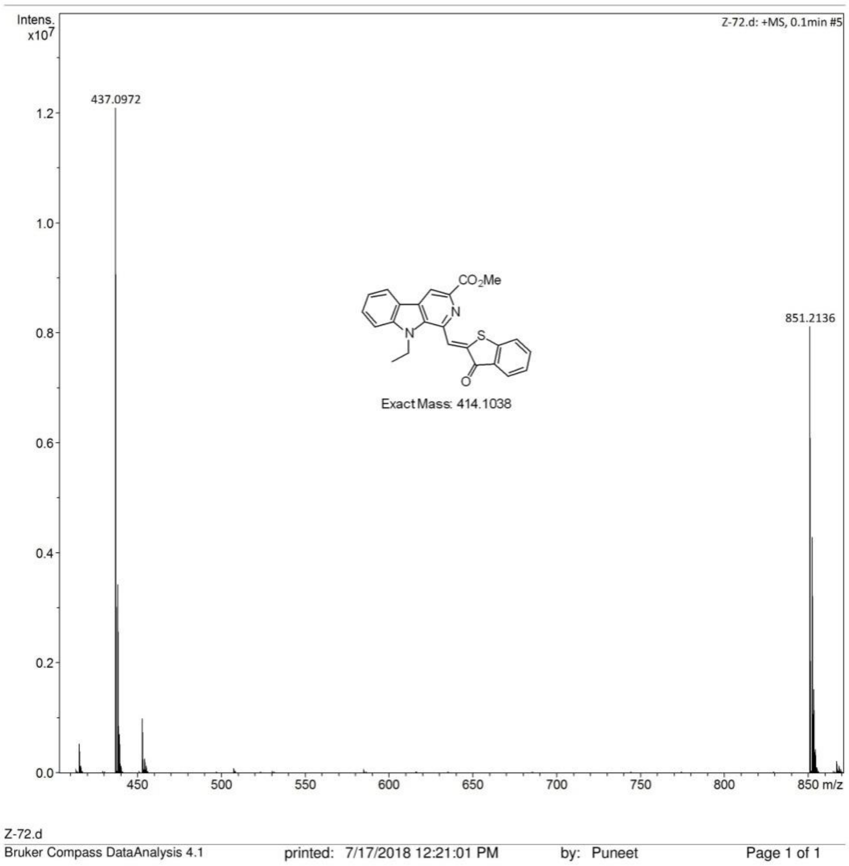

Figure S15. HRMS spectrum of **2cA**.

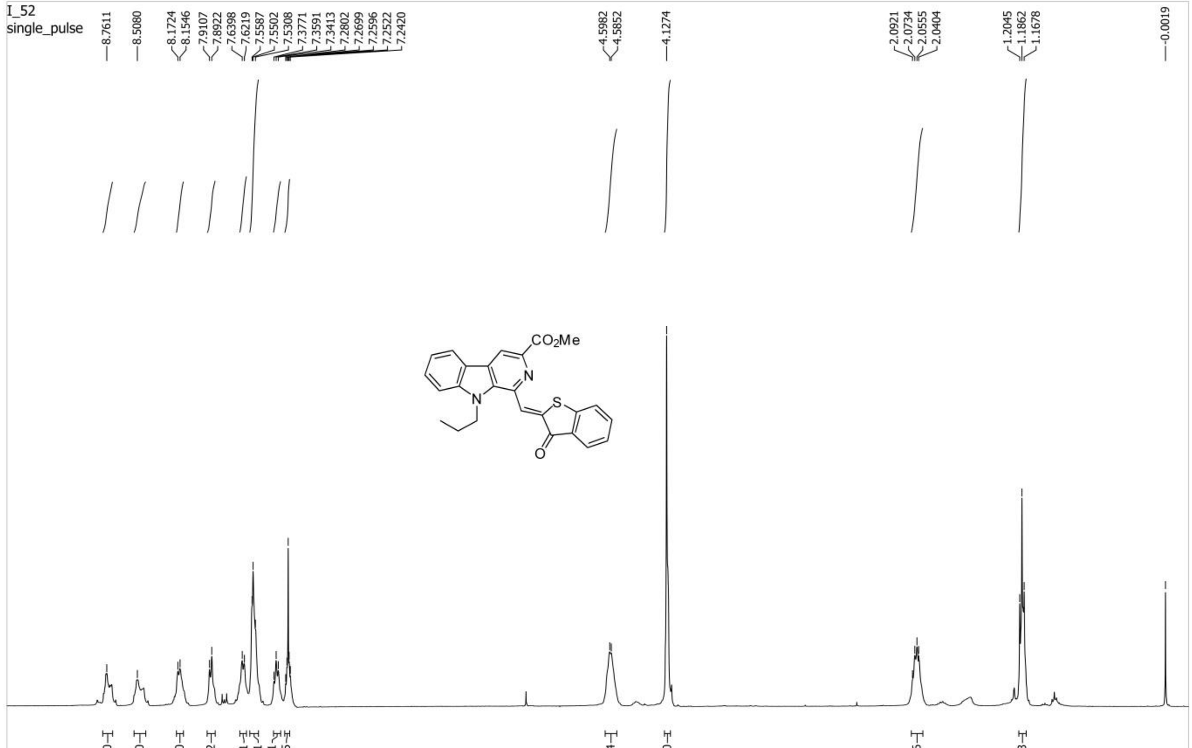

Figure S16. ^{1}H NMR spectrumof **2dA**.

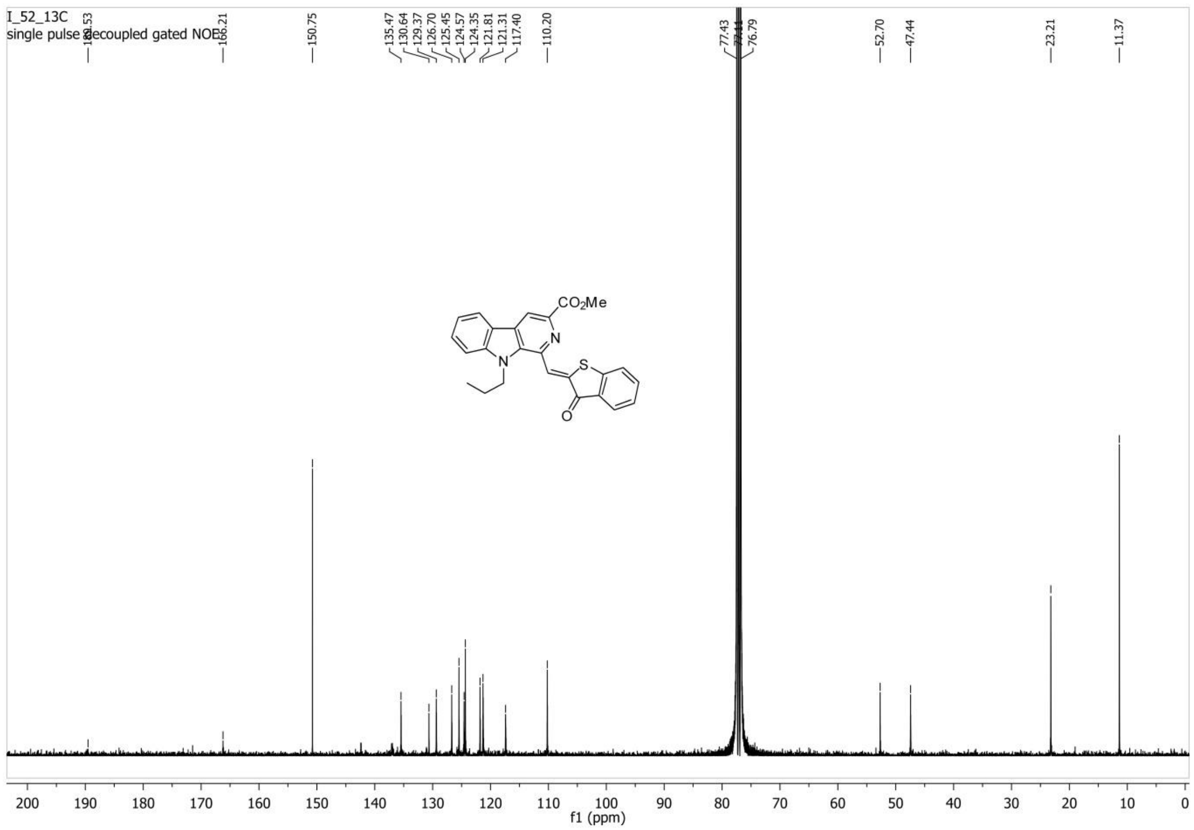

Figure S17. ^{13}C NMR spectrumof **2dA**.

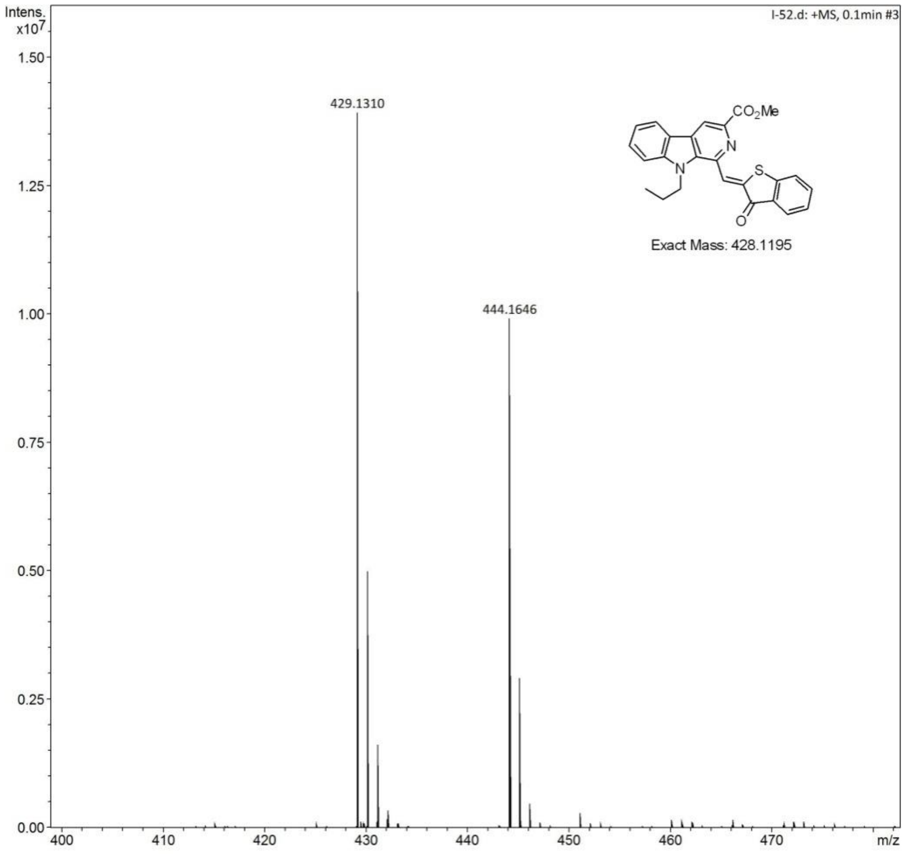

Figure S18. HRMS spectrum of **2dA**.

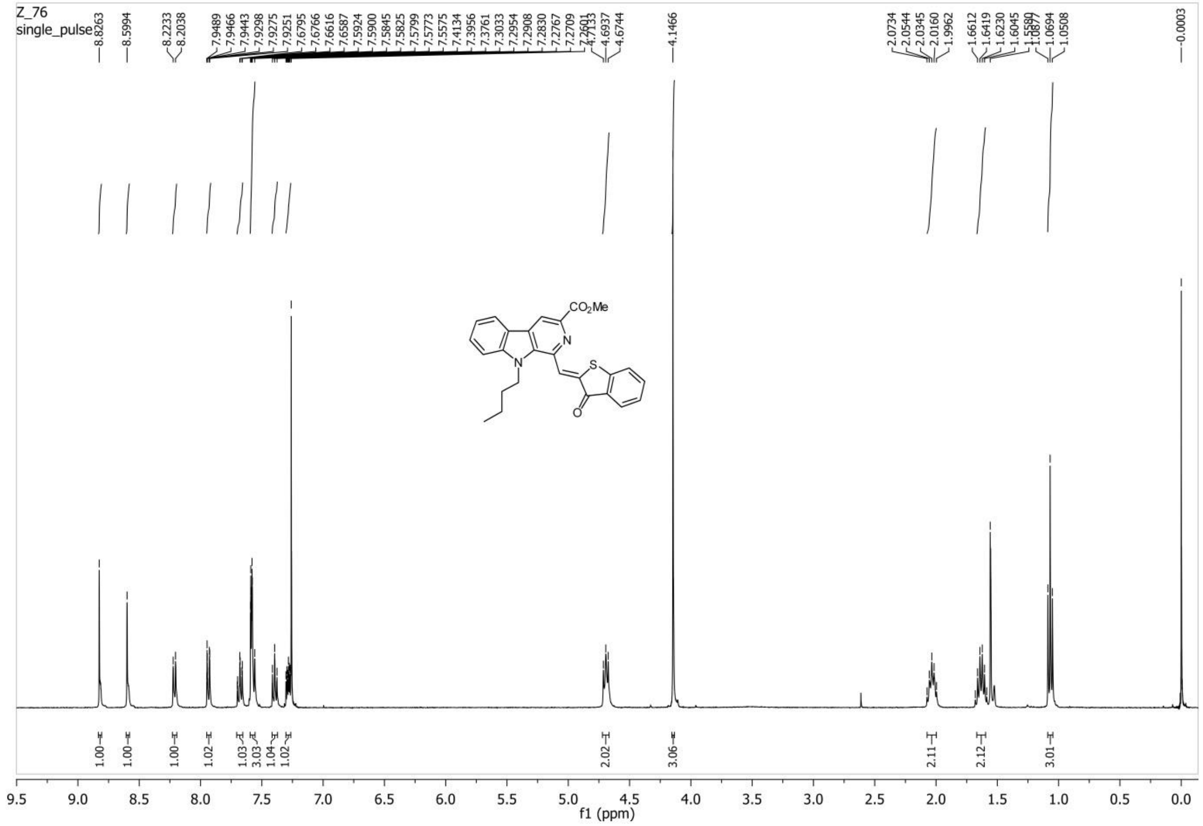

Figure S19. ^{1}H NMR spectrumof **2eA**.

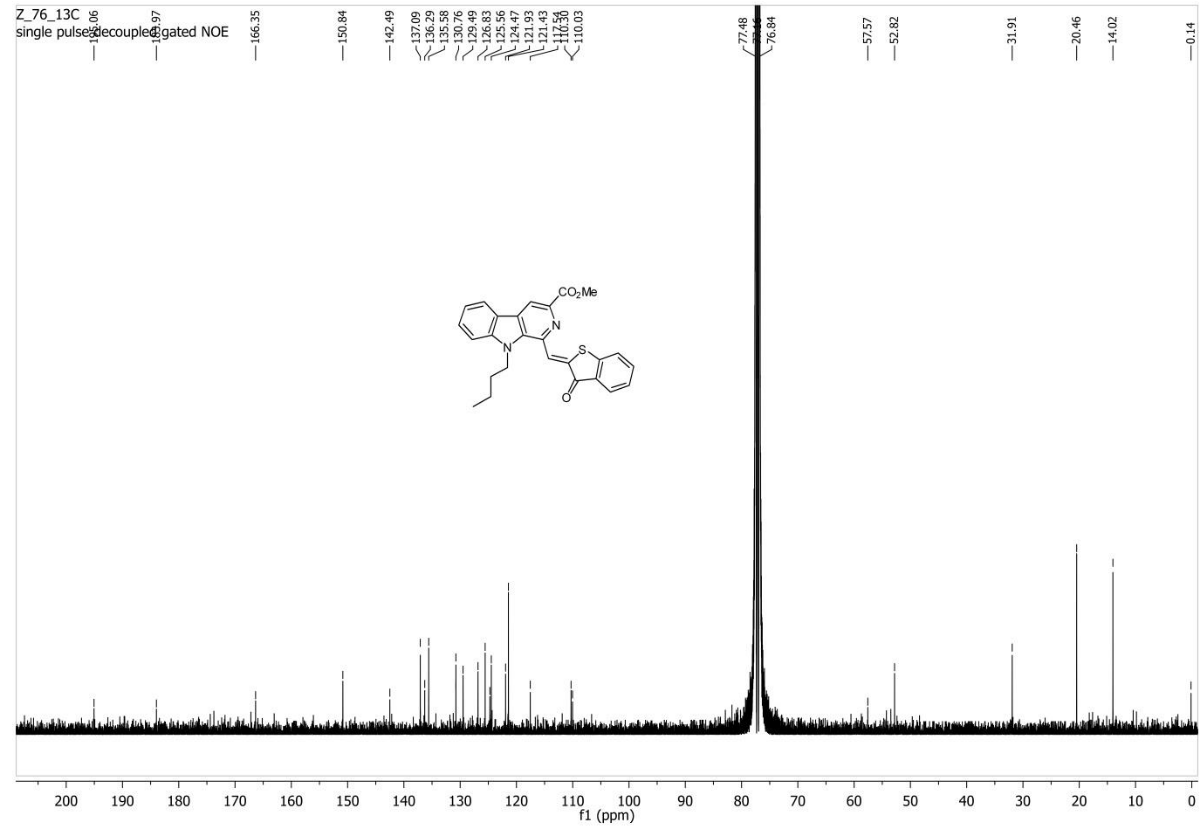

Figure S20. ^{13}C NMR spectrumof **2eA**.

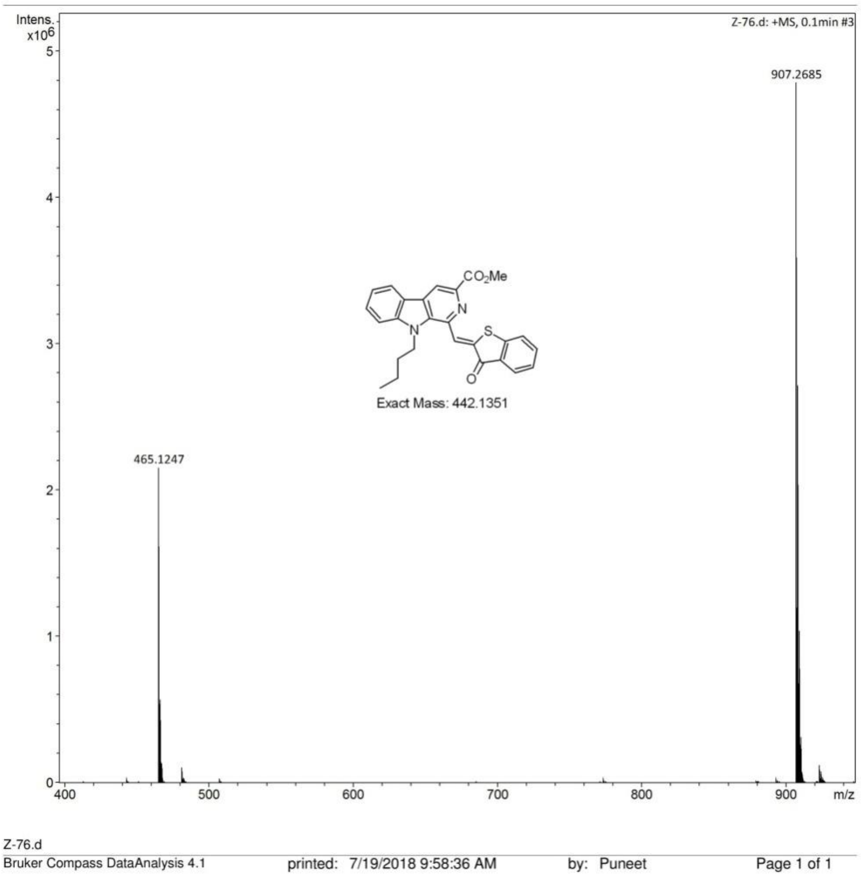

Figure S21. HRMS spectrum of **2eA**.

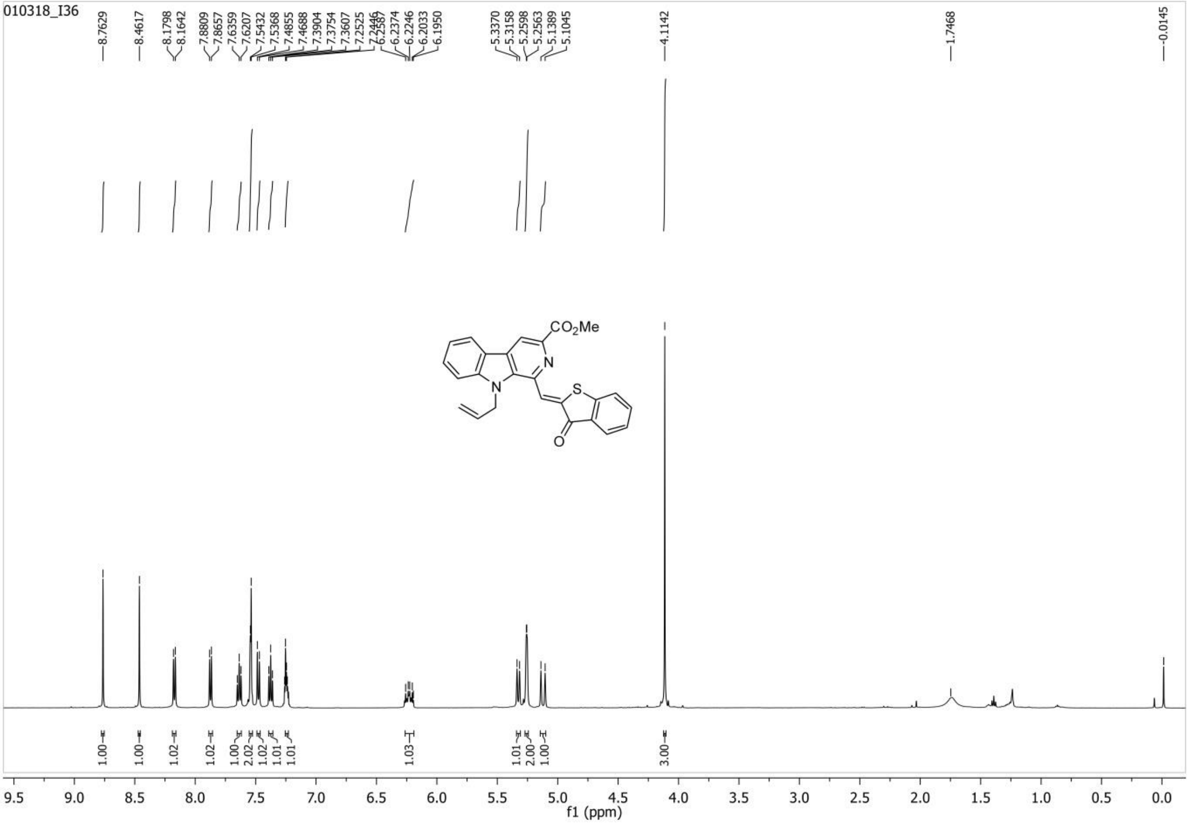

Figure S22. ^{1}H NMR spectrumof **2fA**.

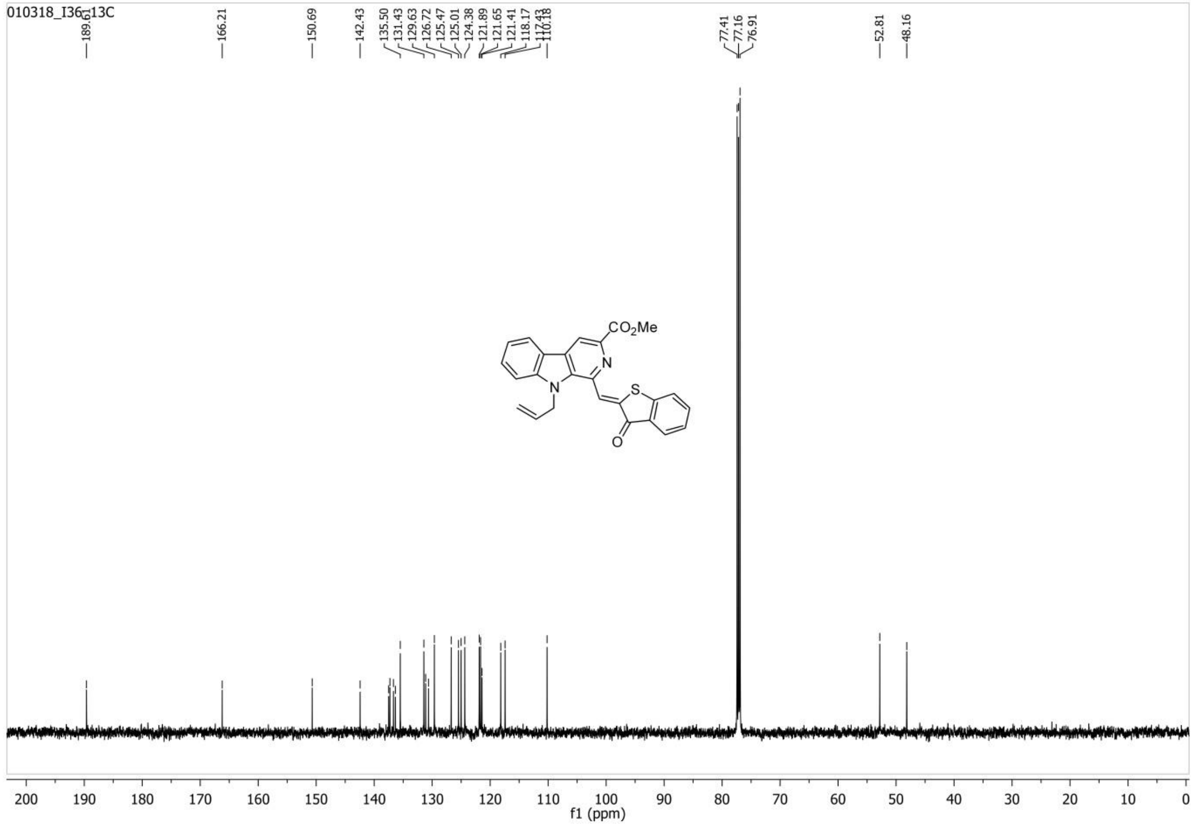

Figure S23. ^{13}C NMR spectrumof **2fA**.

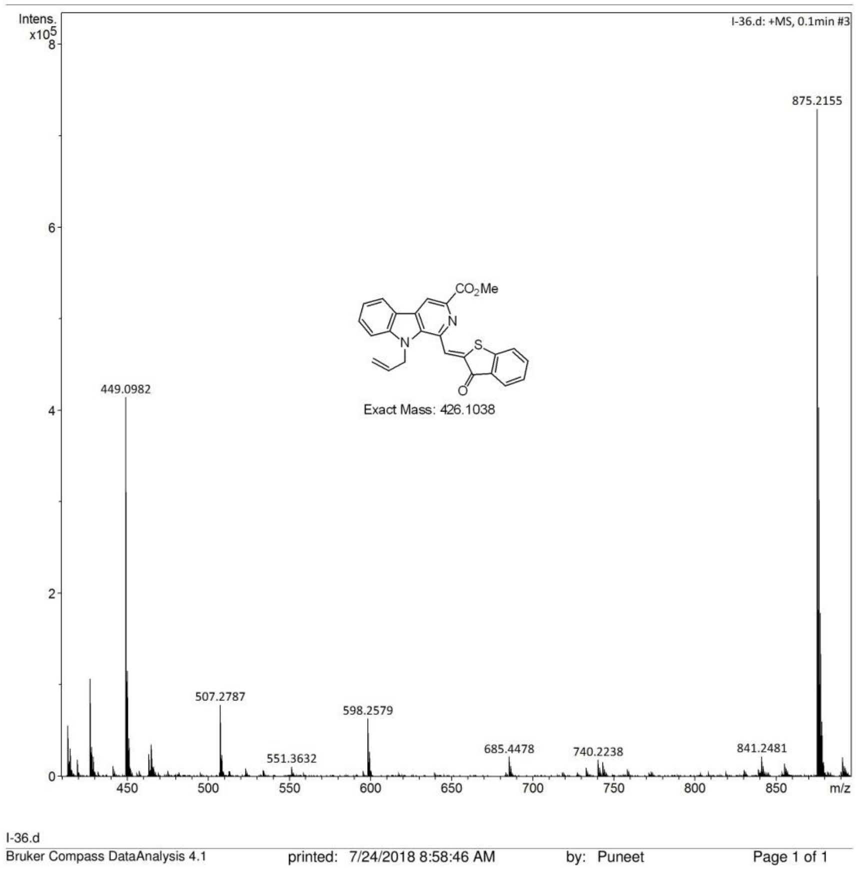

Figure S24. HRMS spectrum of **2fA**.

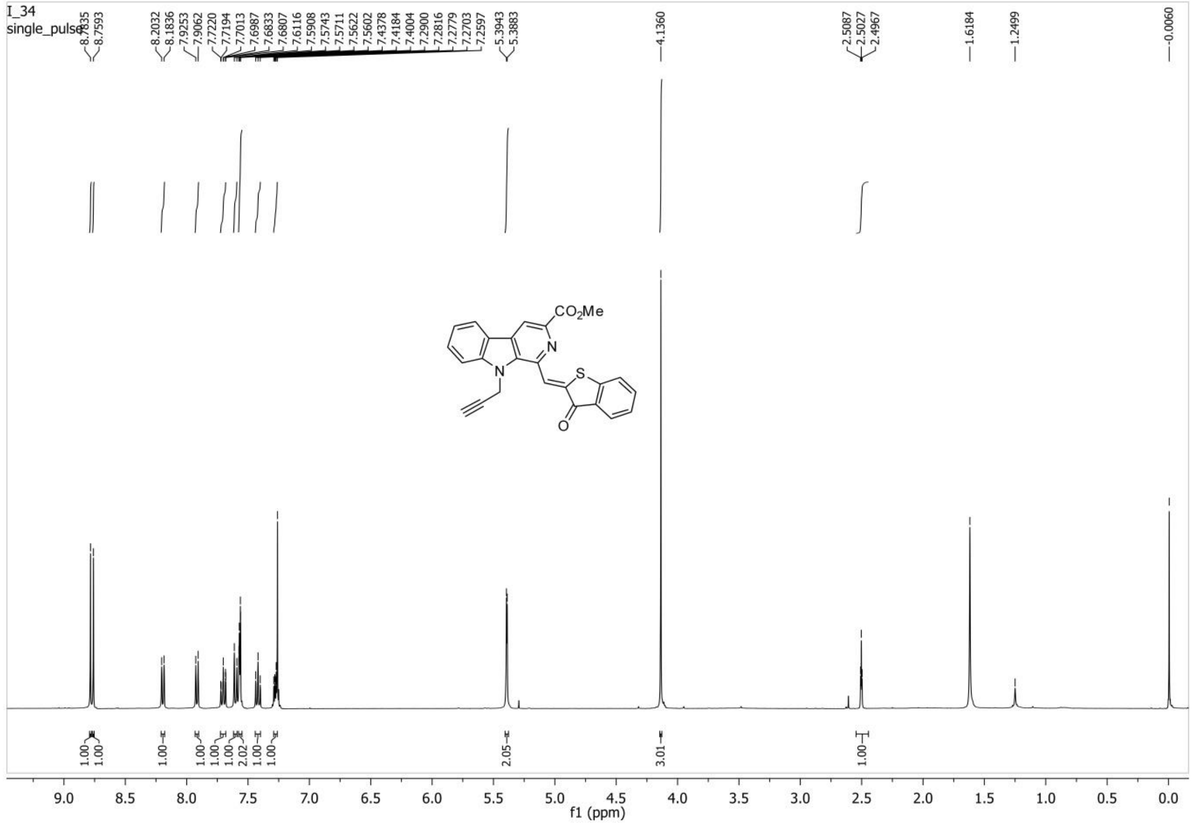

Figure S25. ^{1}H NMR spectrumof **2gA**.

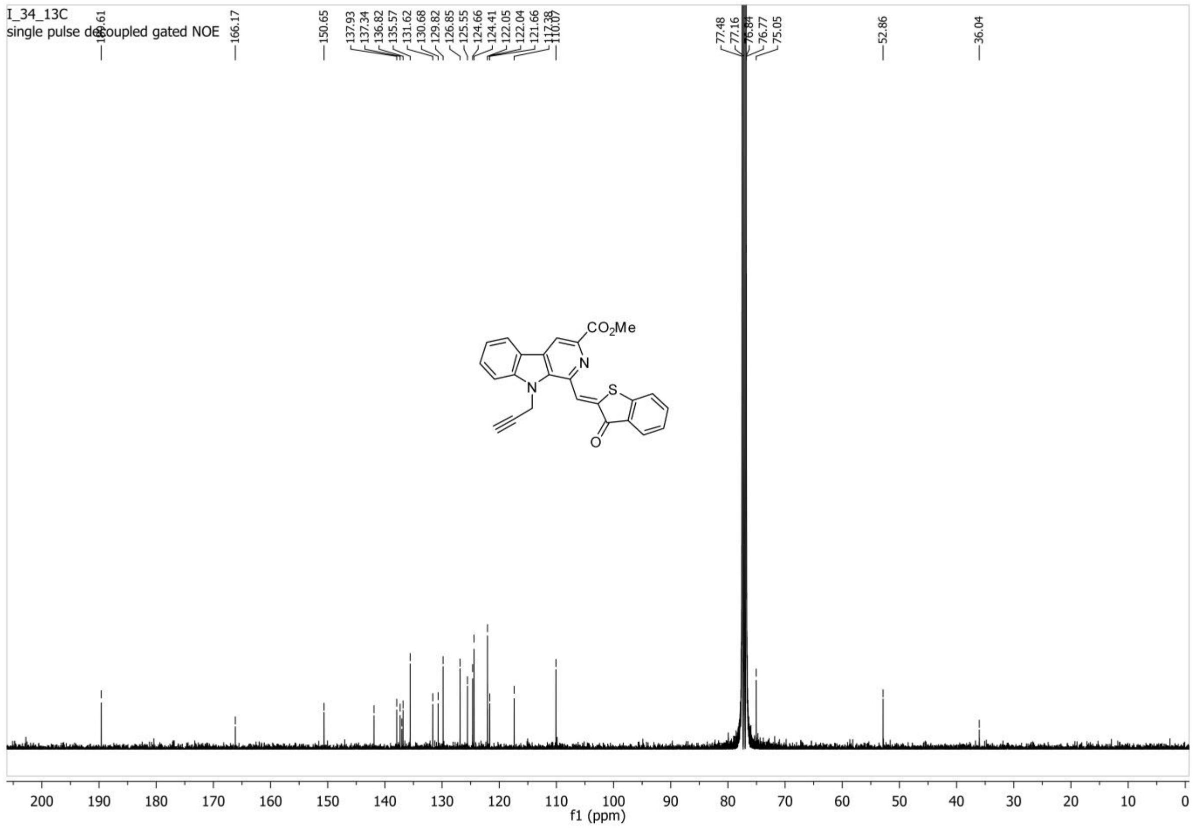

Figure S26. ^{13}C NMR spectrumof **2gA**.

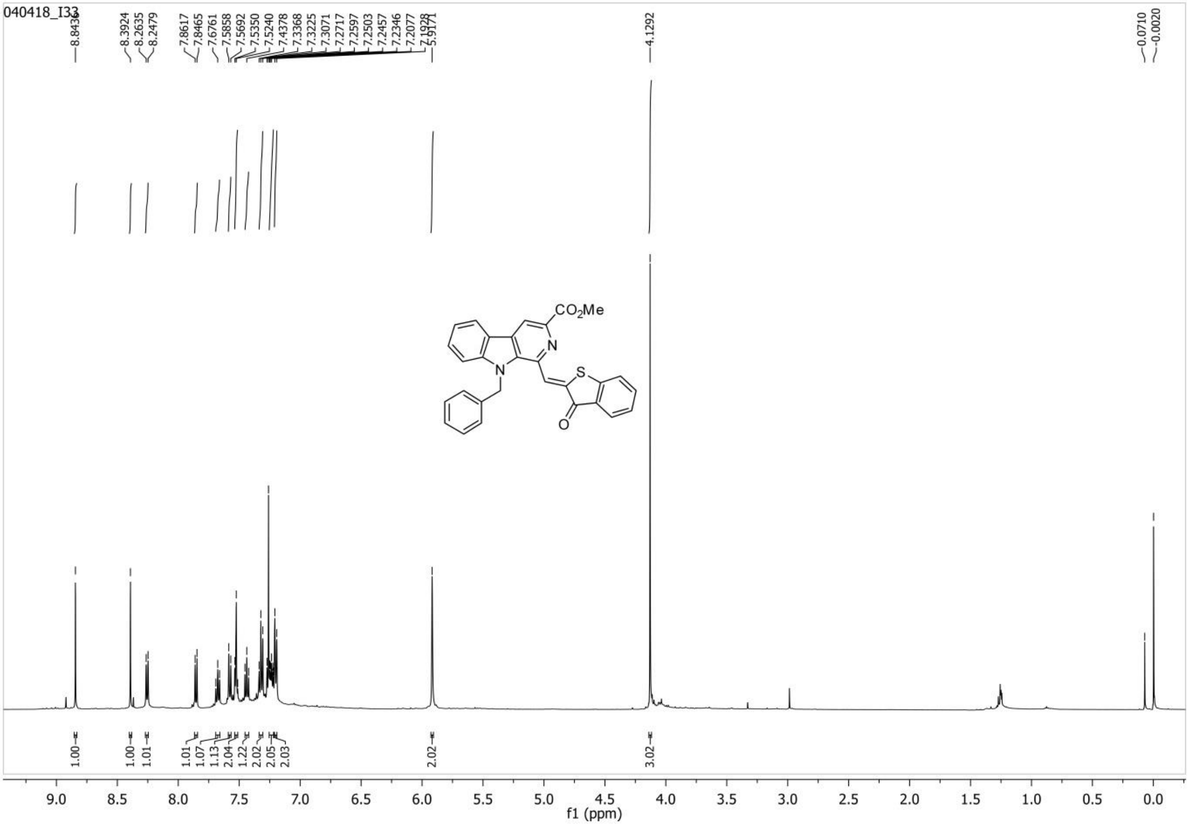

Figure S27. ^{1}H NMR spectrumof **2hA**.

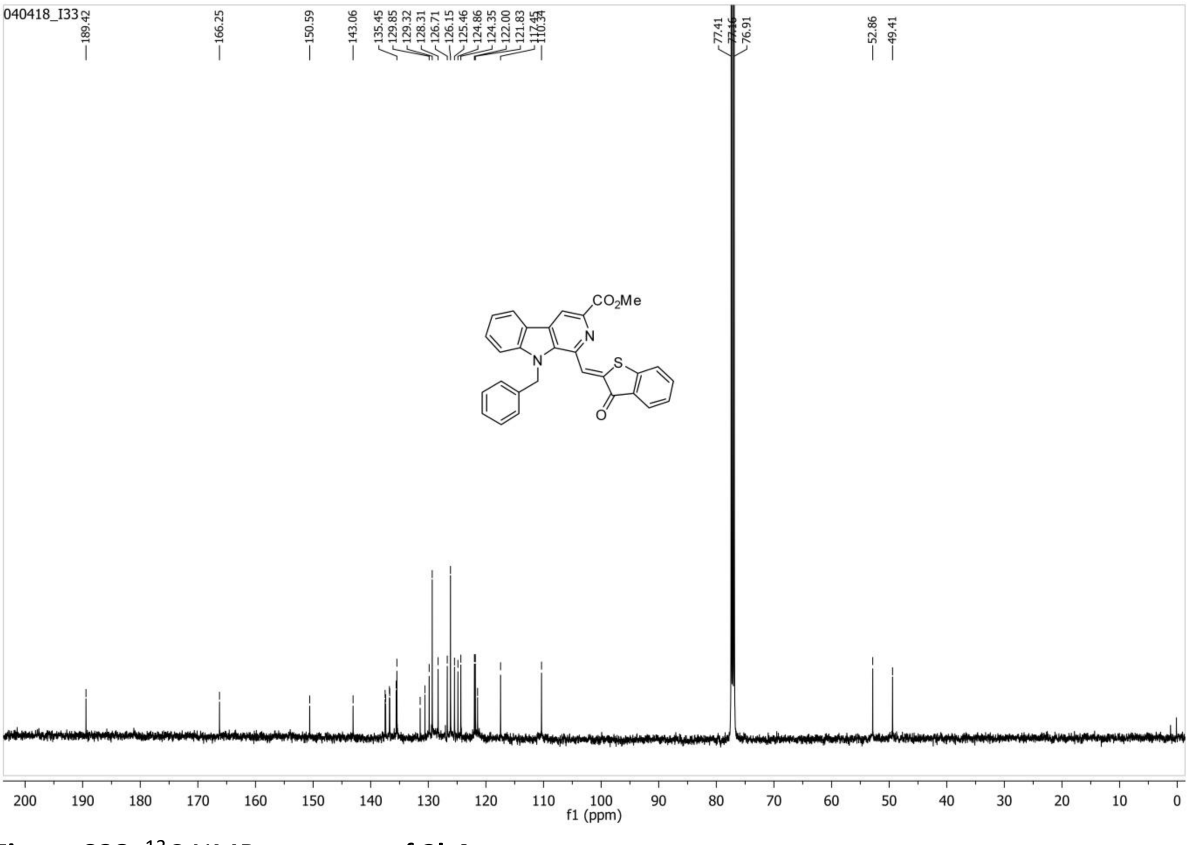

Figure S28. ^{13}C NMR spectrumof **2hA**.

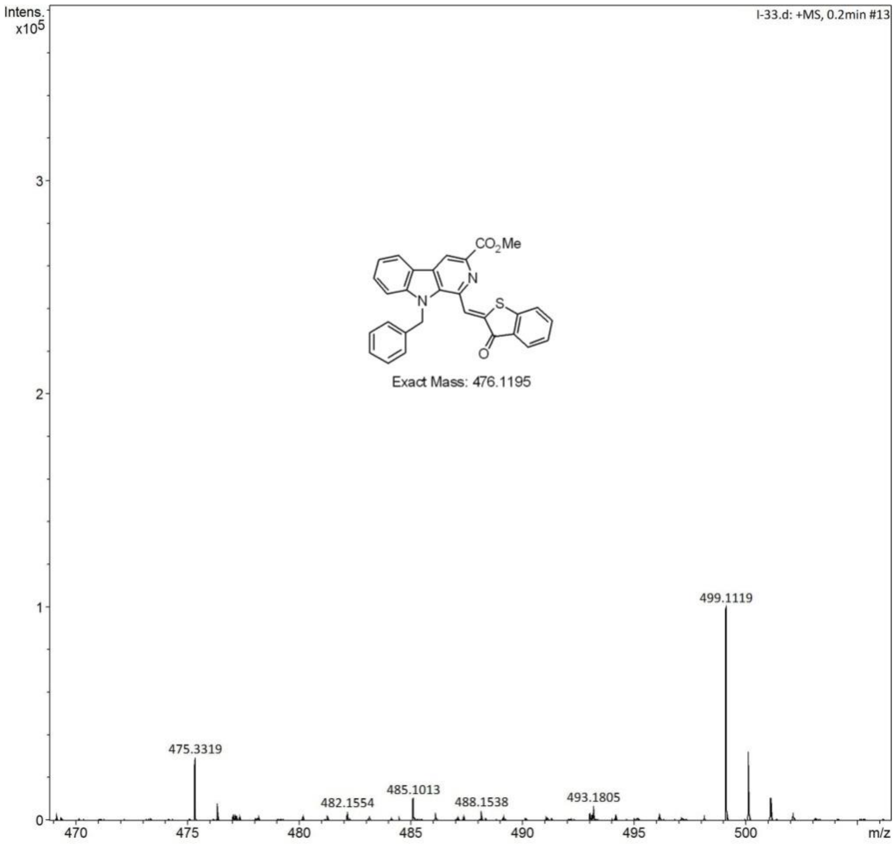

Figure S29. HRMS spectrum of **2hA**.

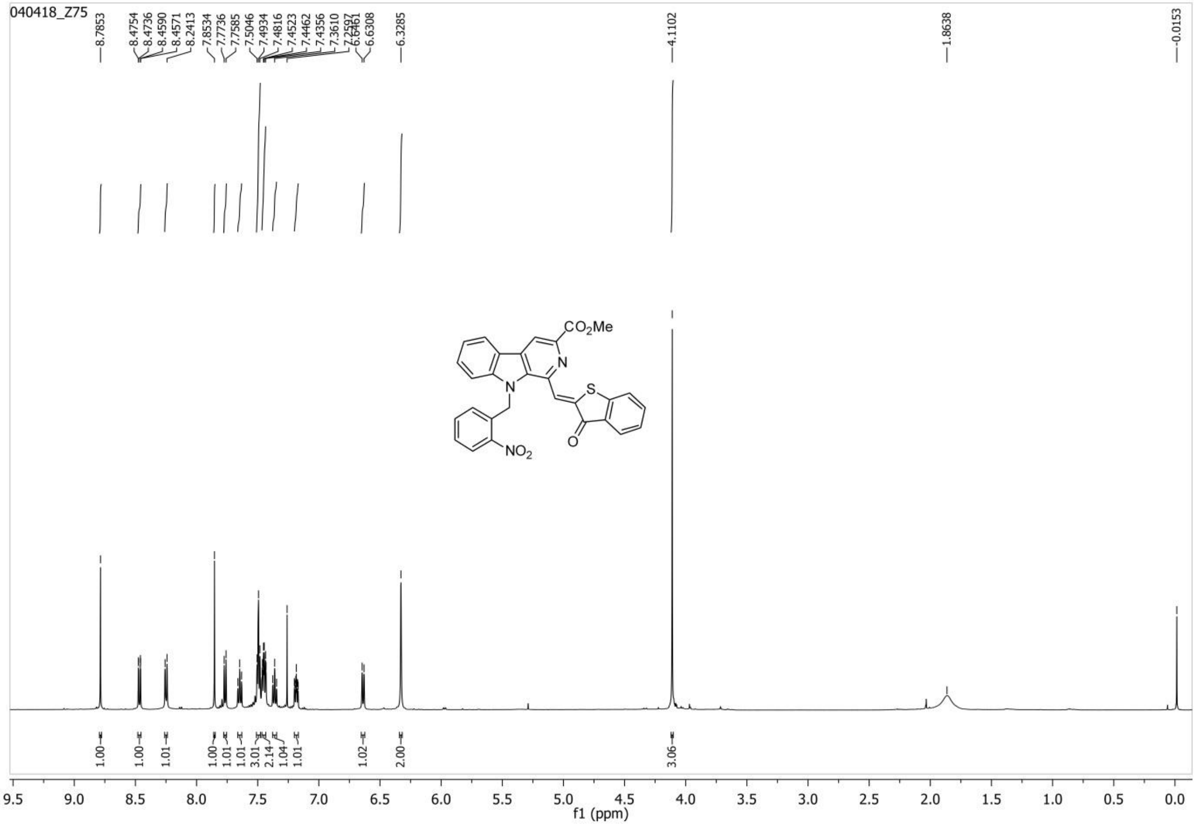

Figure S30. ^{1}H NMR spectrumof **2iA**.

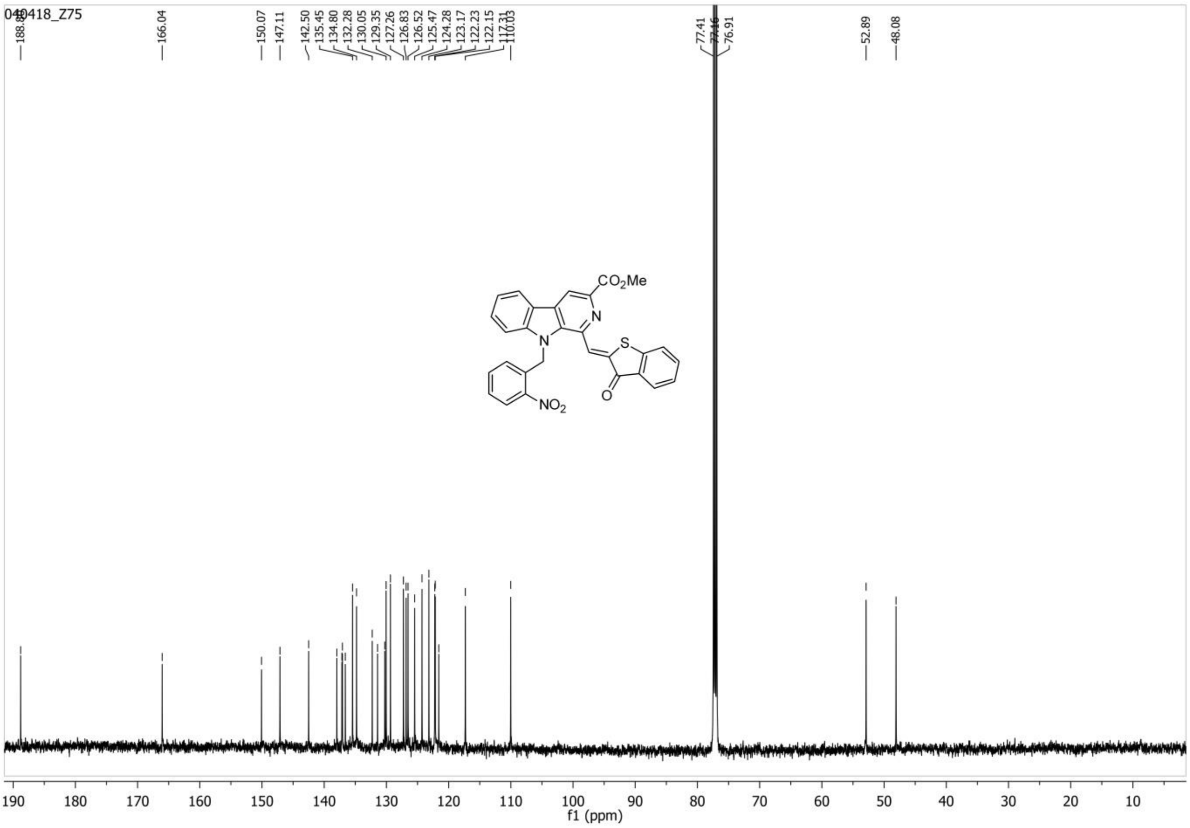

Figure S31. ^{13}C NMR spectrumof **2iA**.

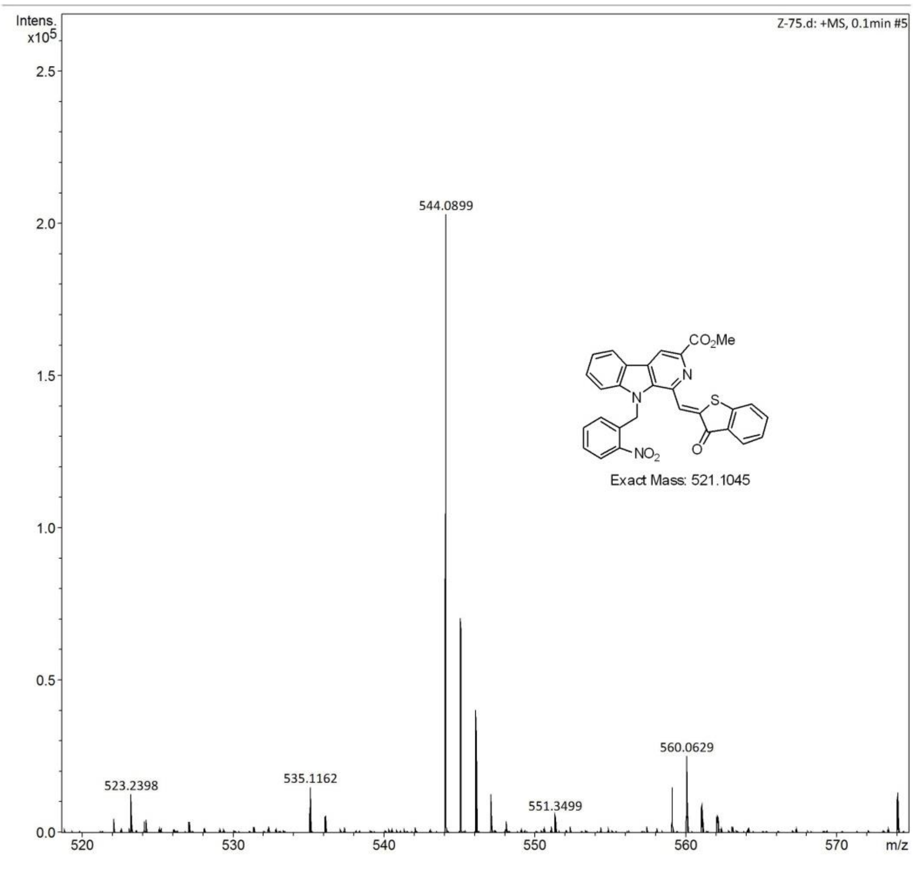

Figure S32. HRMS spectrum of **2iA**.

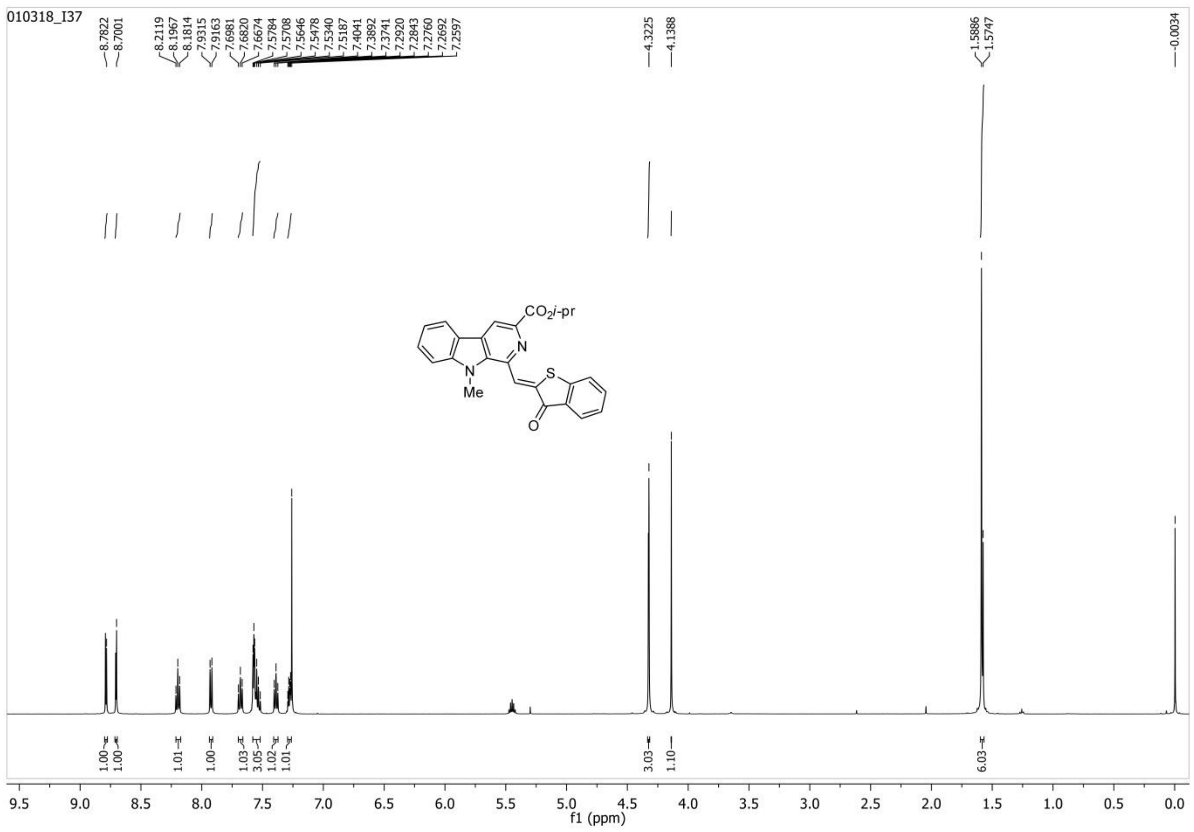

Figure S33. ^{1}H NMR spectrumof **2jA**.

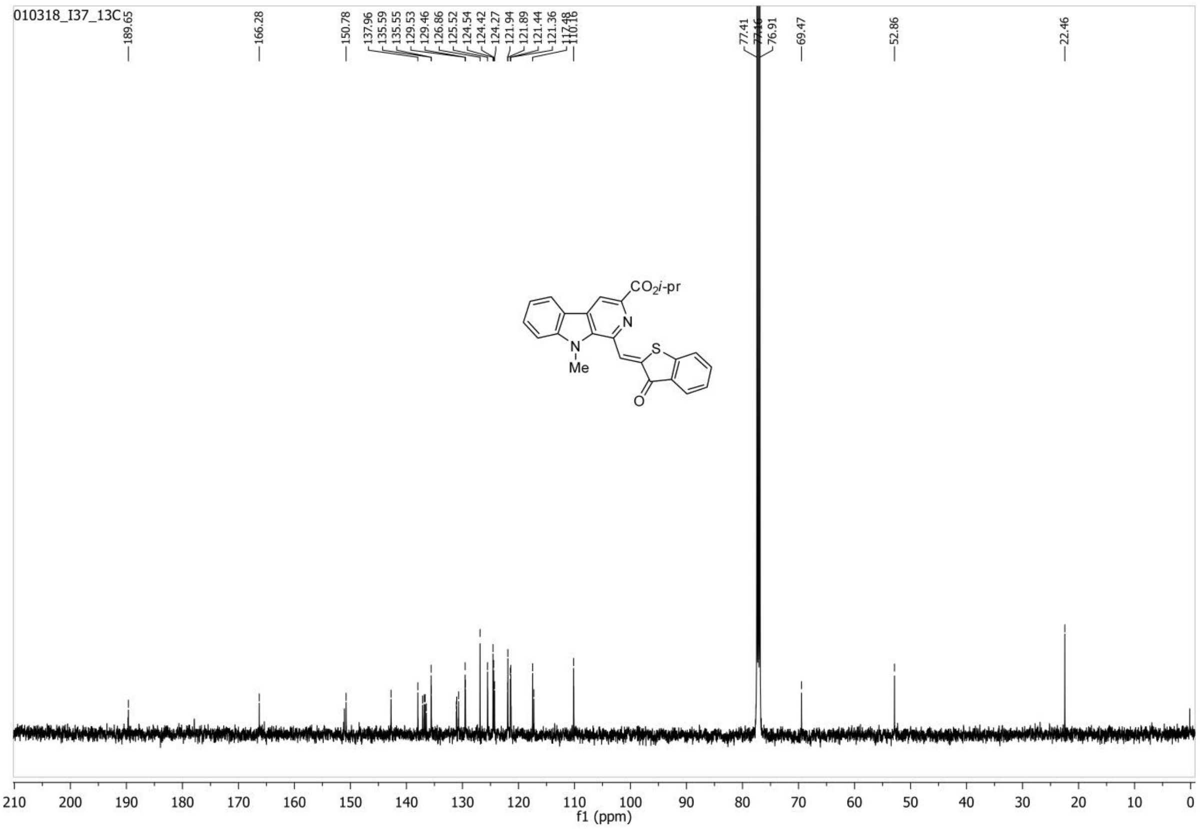

Figure S34. ^{13}C NMR spectrumof **2jA**.

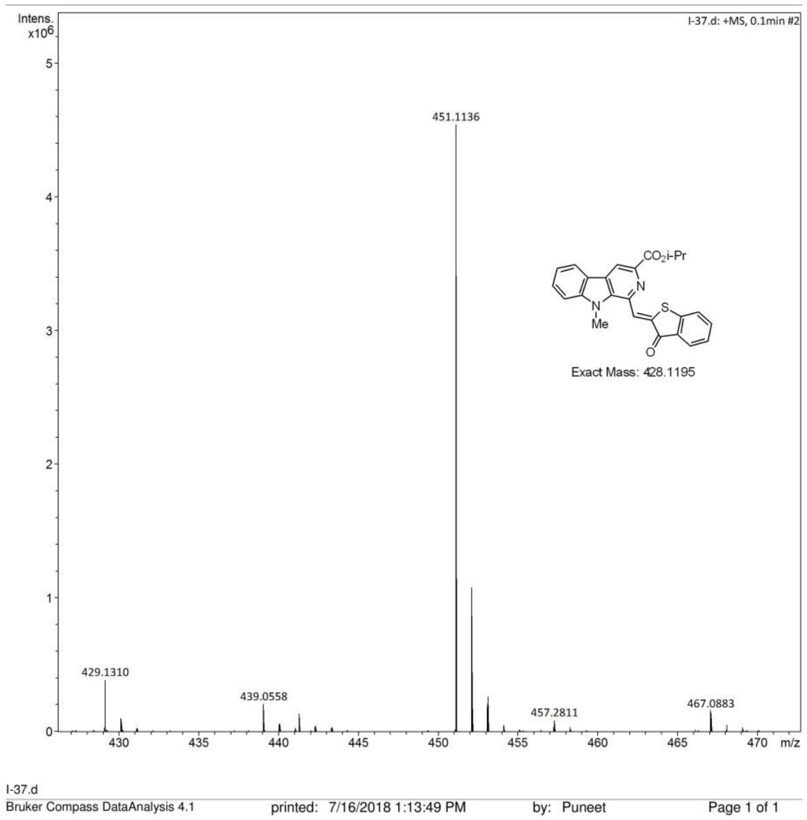

Figure S35. HRMS spectrum of **2jA**.

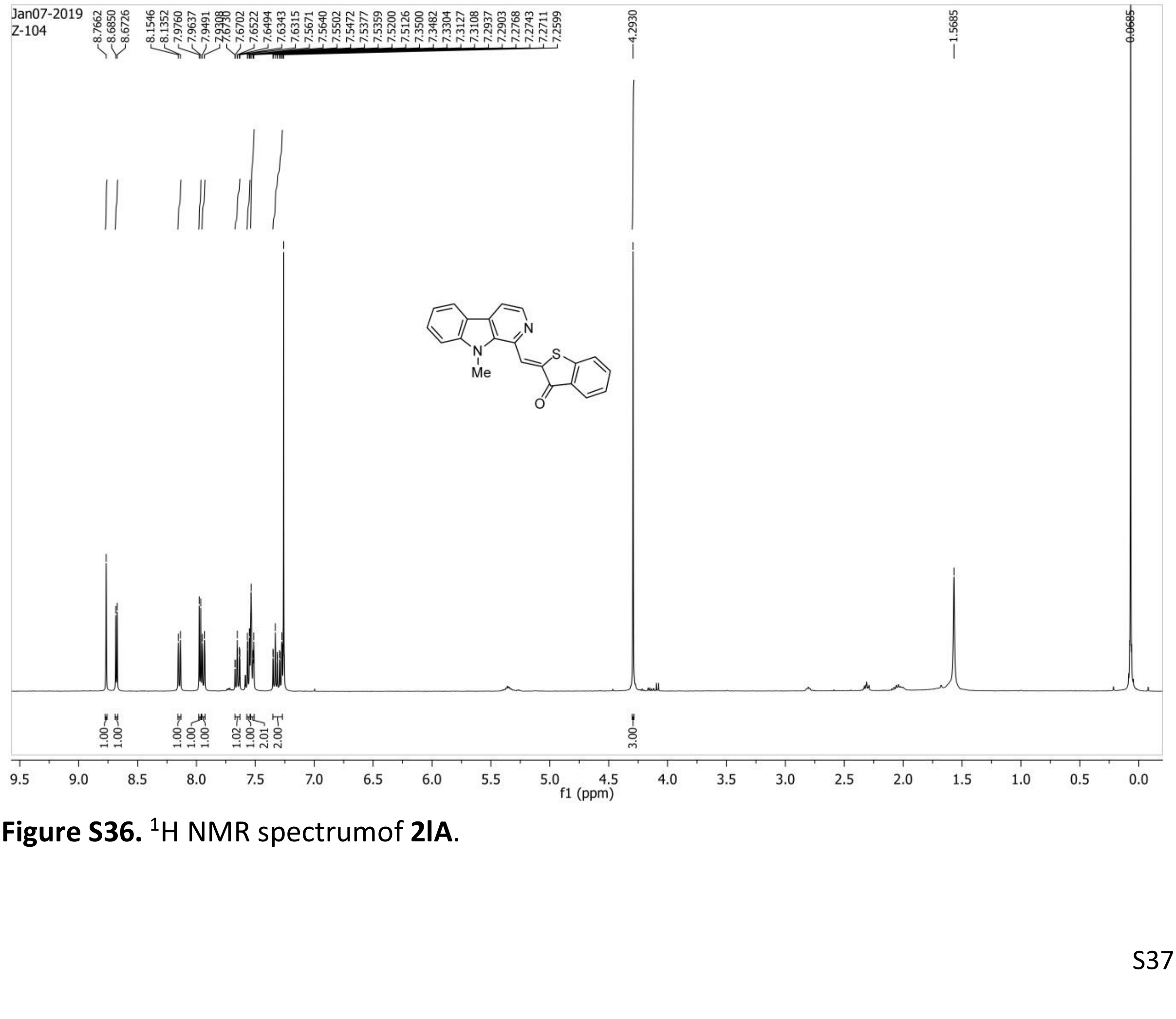

Figure S36. ^{1}H NMR spectrumof **2lA**.

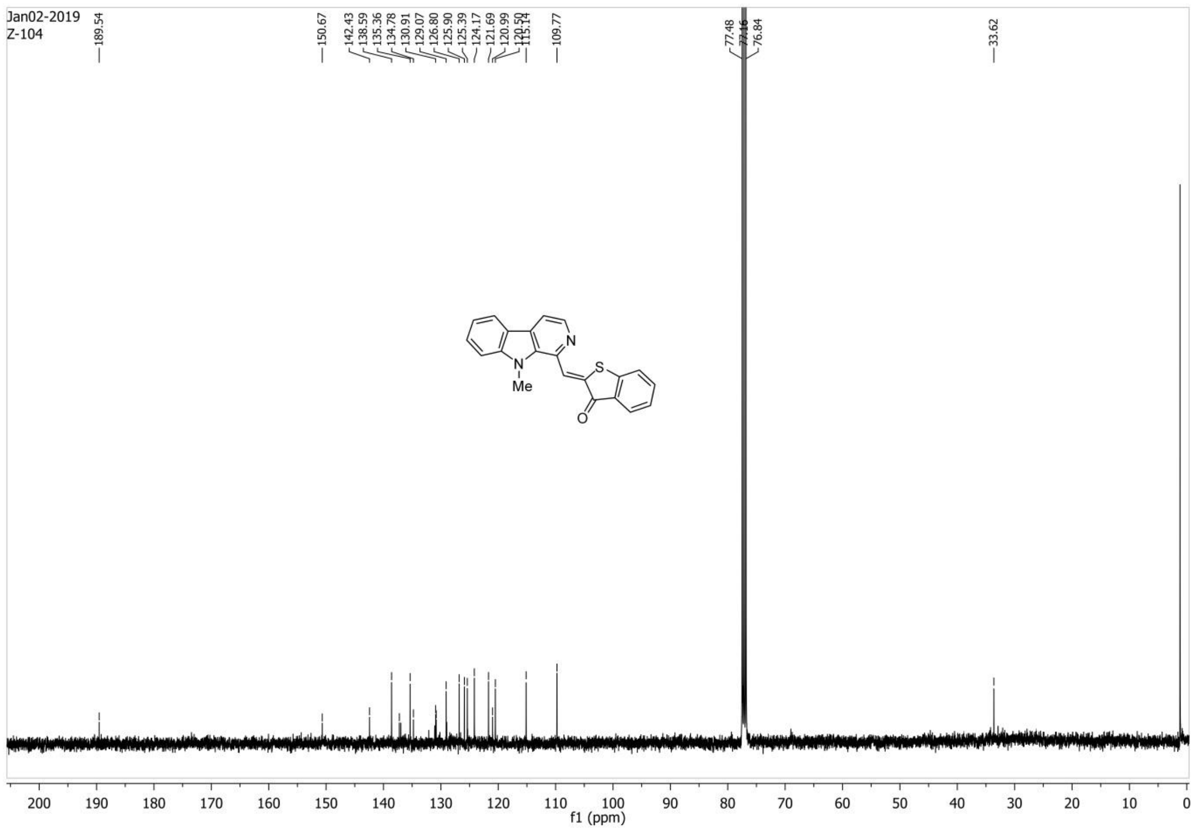

Figure S37. ^{13}C NMR spectrumof **2lA**.

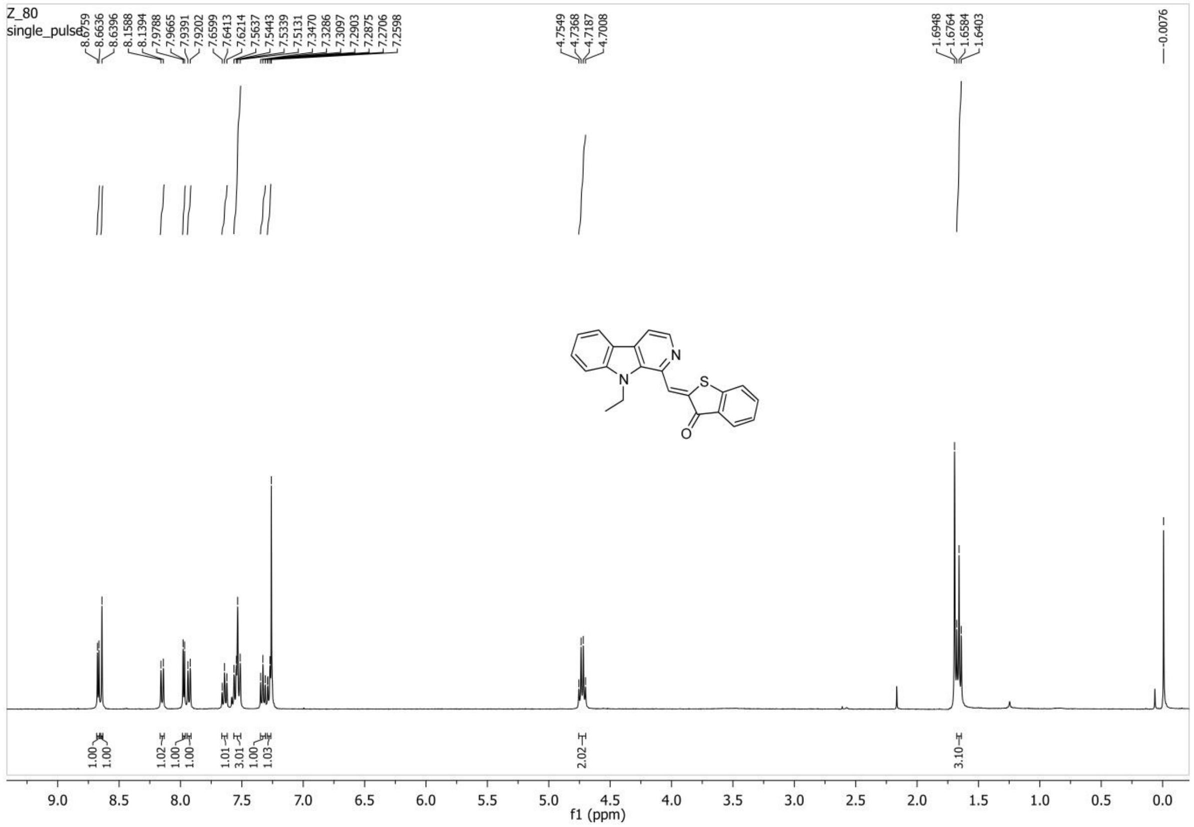

Figure S38. ^{1}H NMR spectrumof **2mA**.

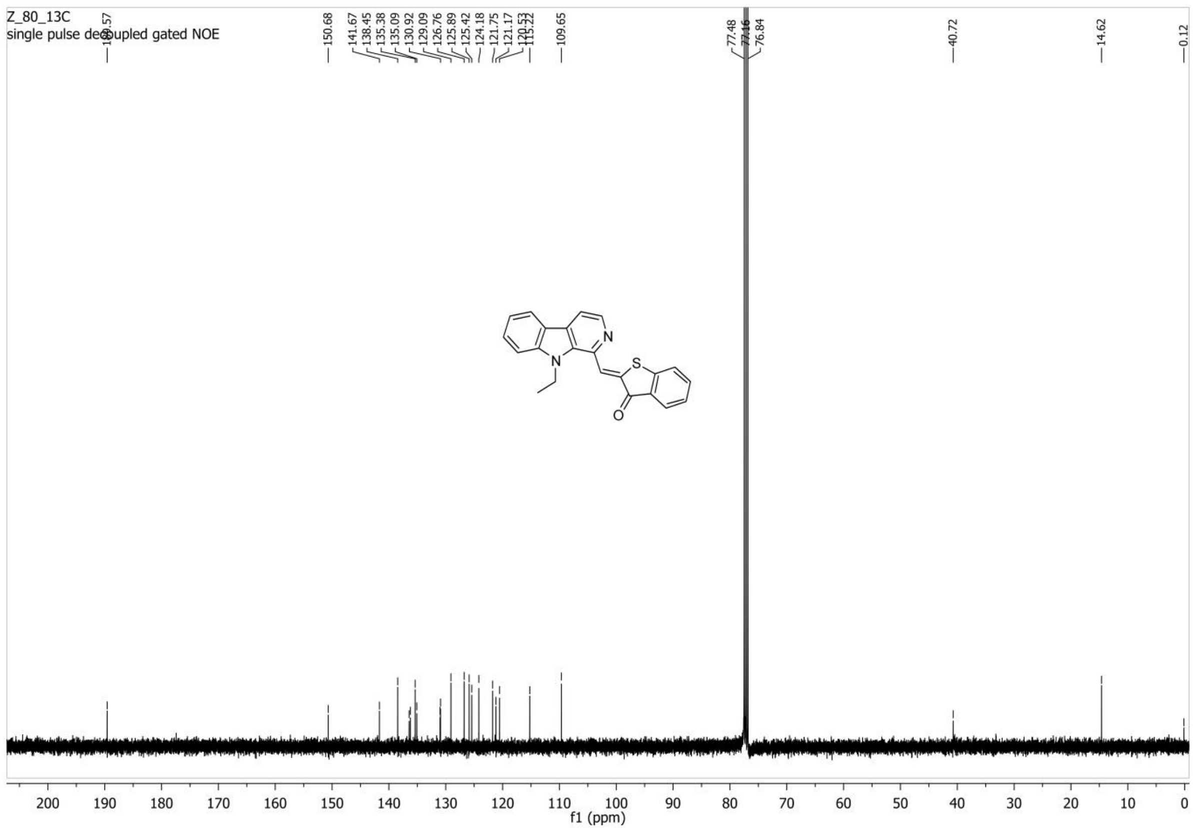

Figure S39. ^{13}C NMR spectrumof **2mA**.

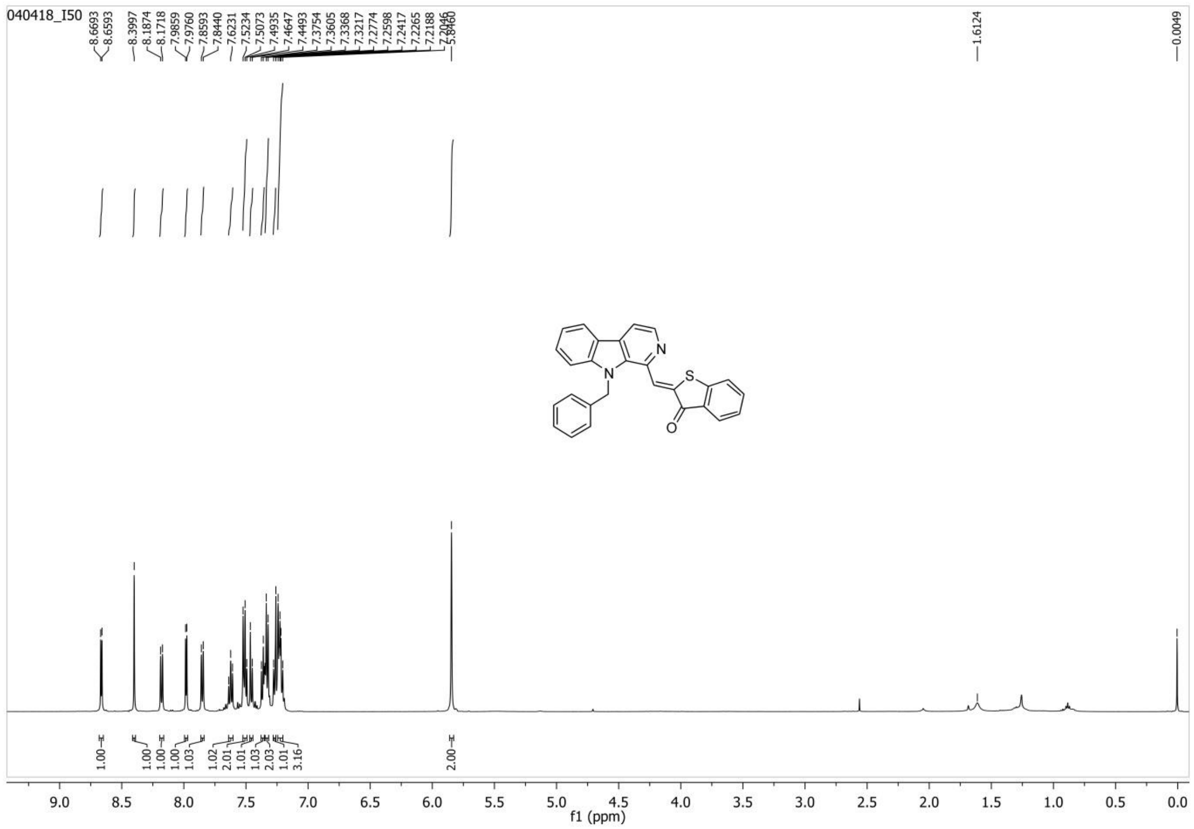

Figure S40. ^{1}H NMR spectrumof **2nA**.

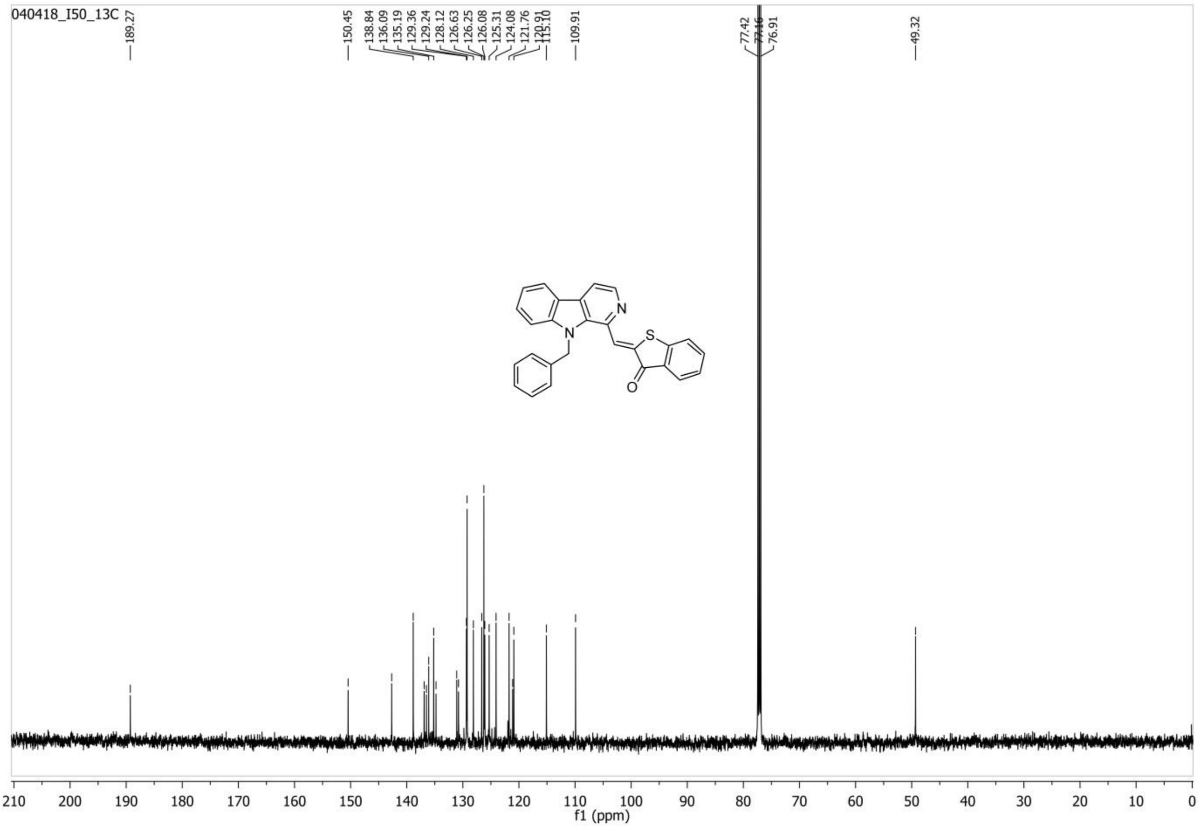

Figure S41. ^{13}C NMR spectrumof **2nA**.

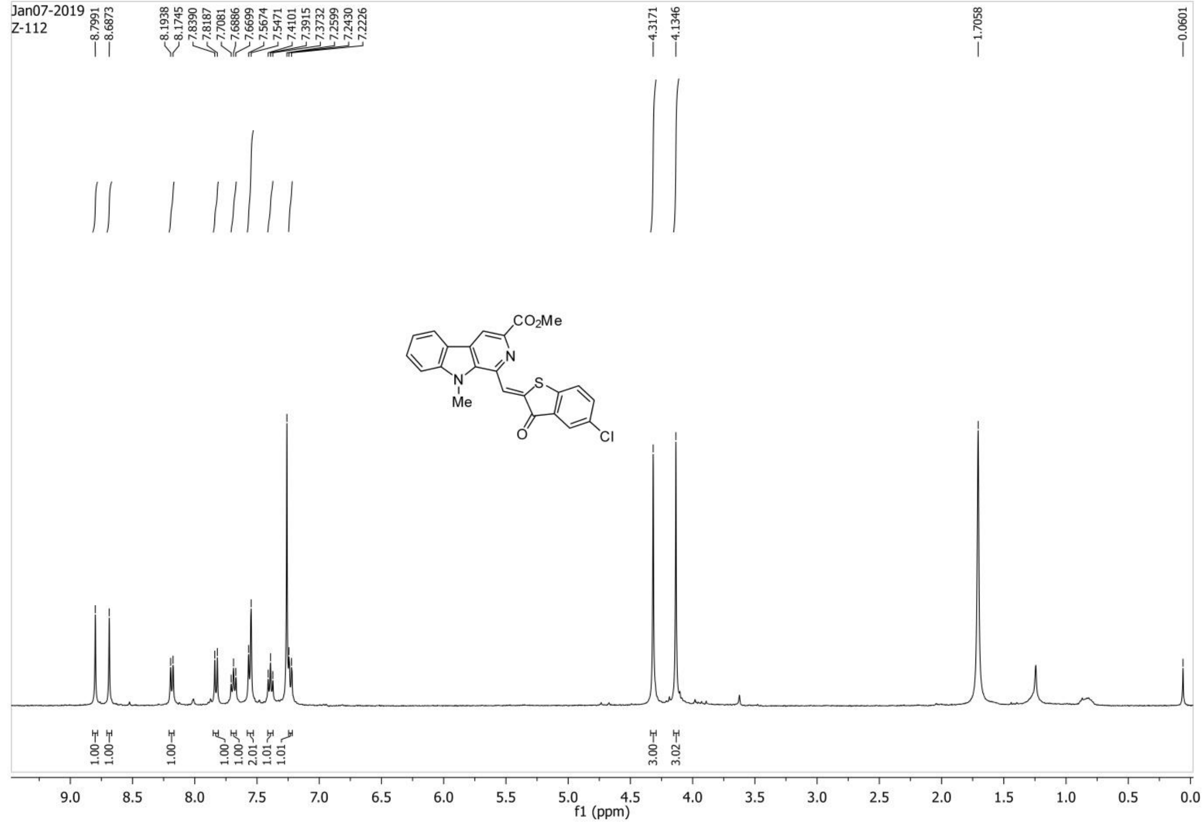

Figure S42. ^{1}H NMR spectrumof **2bB**.

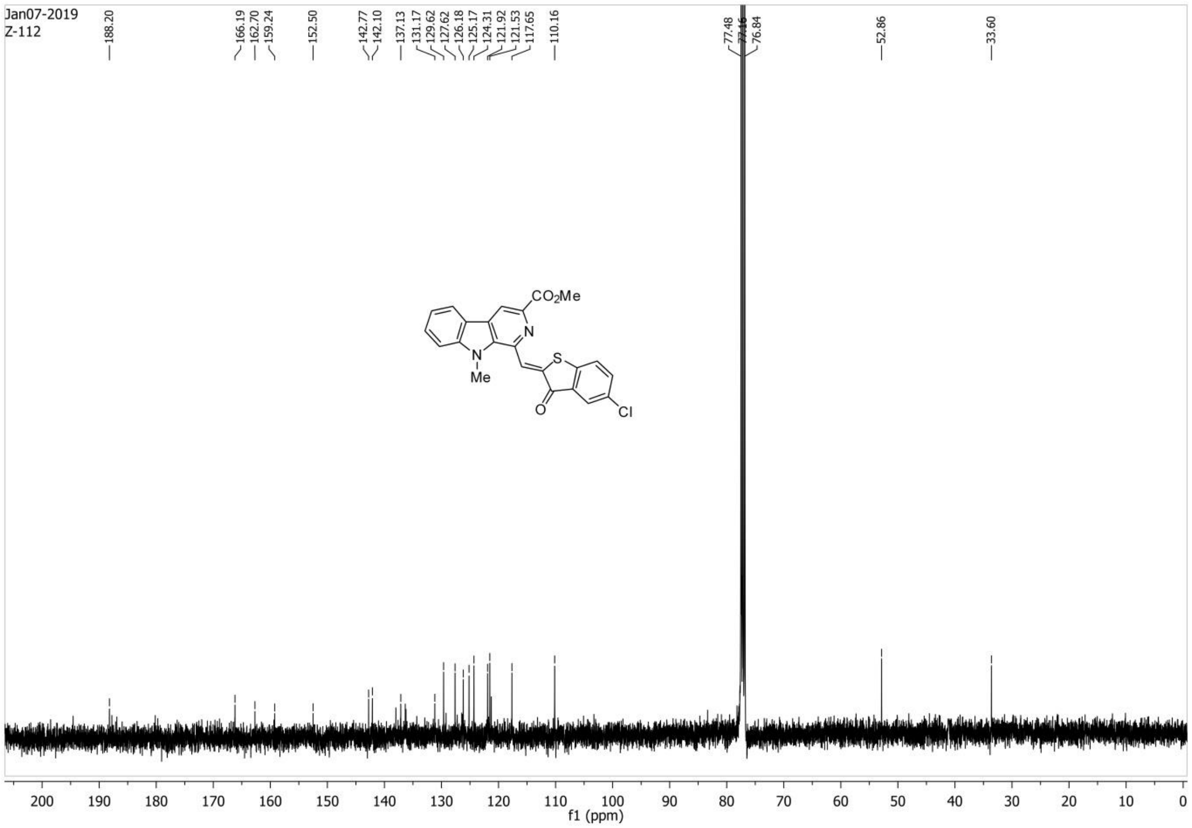

Figure S43. ^{13}C NMR spectrumof **2bB**.

Compound Table

Compound Label	RT	Mass	Formula	MFG Formula	DB Formula
Cpd 3: 0.084	0.084	434.0499	C20 H10 N4 O8	C20 H10 N4 O8	C20 H10 N4 O8

Compound Label	*m/z*	RT	Algorithm	Mass
Cpd 3: 0.084	435.057	0.084	Find by Molecular Feature	434.0499

MFE MS Spectrum

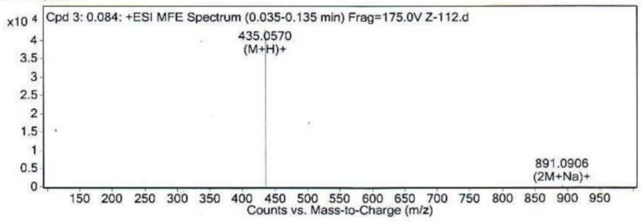

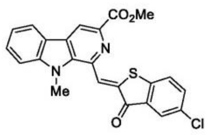

Exact Mass: 434.0492

MFE MS Zoomed Spectrum

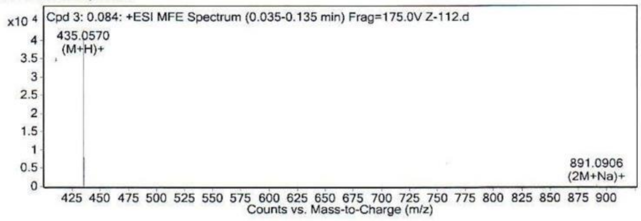

MS Spectrum Peak List

m/z	z	Abund	Ion
435.057	1	43030.43	(M+H)+
436.0608	1	8046.88	(M+H)+
891.0906	1	546.91	(2M+Na)+

--- End Of Report ---

Figure S44. HRMS spectrum of **2bB**.

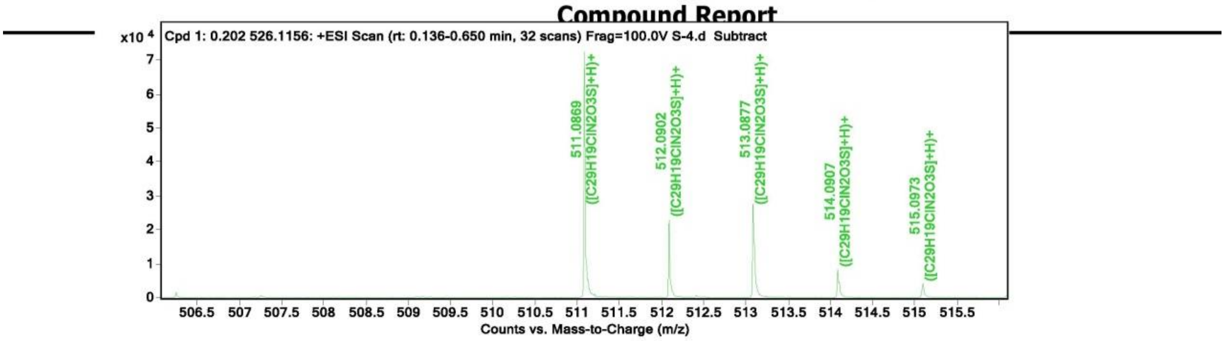

MS Spectrum Peak List

m/z	*Calc m/z*	Diff(ppm)	z	Abund	Formula	Ion
511.0869	511.0878	1.62	1	72567.85	C29H19ClN2O3S	(M+H)+
512.0902	512.0909	1.38	1	23229.96	C29H19ClN2O3S	(M+H)+
513.0877	513.086	-3.39	1	27617.25	C29H19ClN2O3S	(M+H)+
514.0907	514.0883	-4.64	1	8417.9	C29H19ClN2O3S	(M+H)+
515.0973	515.087	-20.03	1	4172.86	C29H19ClN2O3S	(M+H)+
516.101	516.0876	-26.08	1	1345.47	C29H19ClN2O3S	(M+H)+
517.1011	517.089	-23.48	1	334.21	C29H19ClN2O3S	(M+H)+

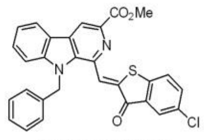

Exact Mass: 510.0805

Instrument Info : Agilent Technologies 6545 Q-TOF LC/MS

Note: Please acknowledge the work done by HRMS Facility at BITS Pilani, Pilani Campus funded by DST-FIST in your publication.

---End Of Report---

Figure S45. HRMS spectrum of **2hB**.

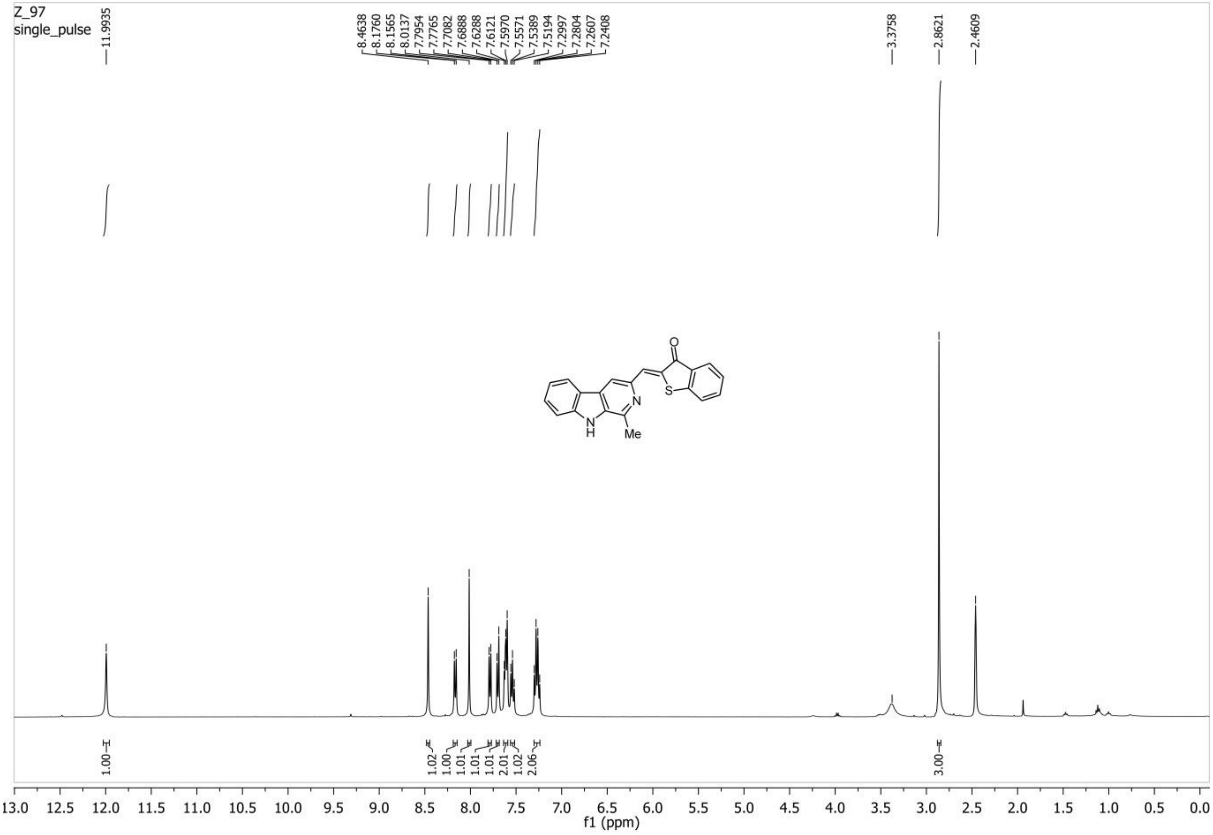

Figure S46. ^{1}H NMR spectrumof **4aA**.

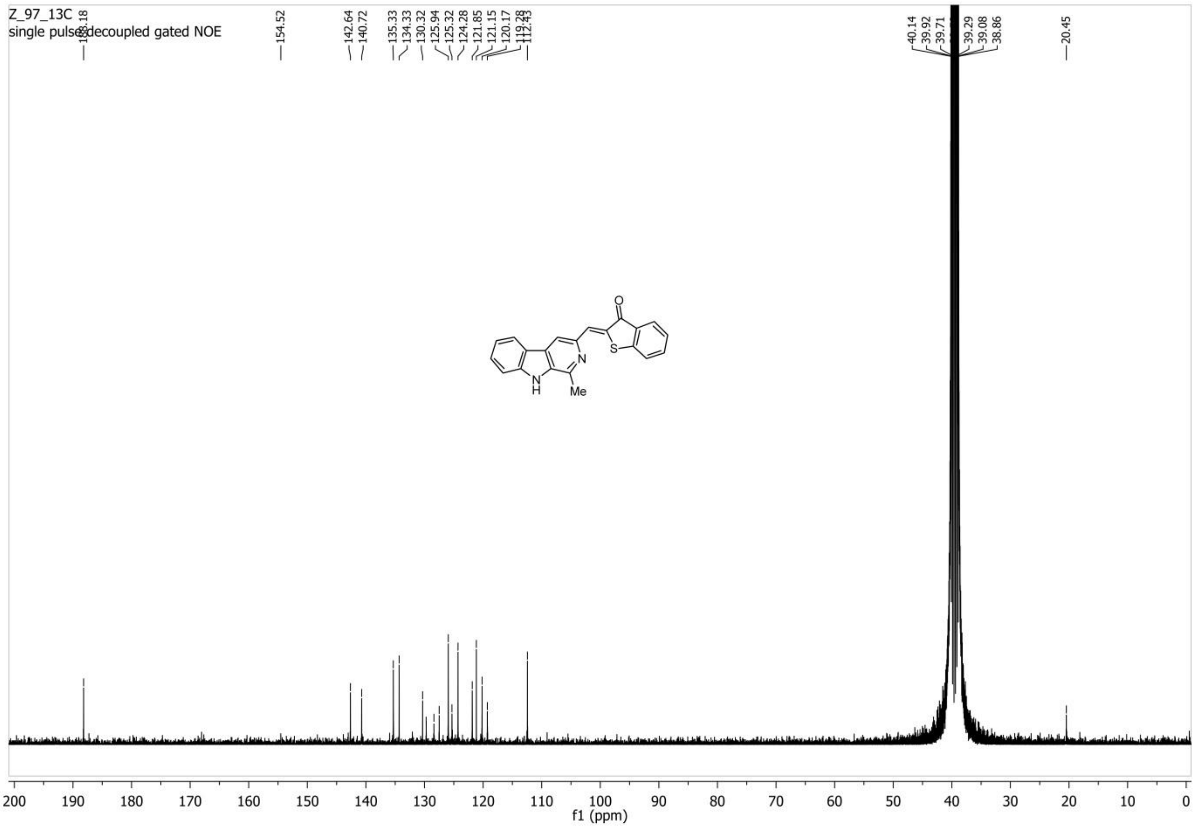

Figure S47. ^{13}C NMR spectrumof **4aA**.

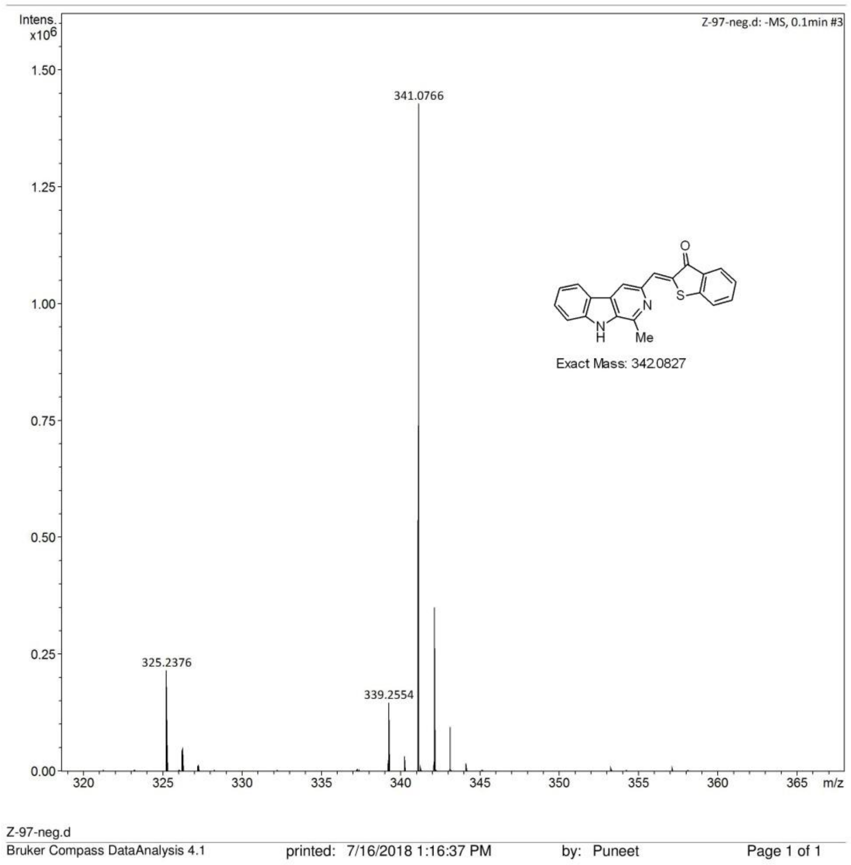

Figure S48. HRMS spectrum of **4aA**.

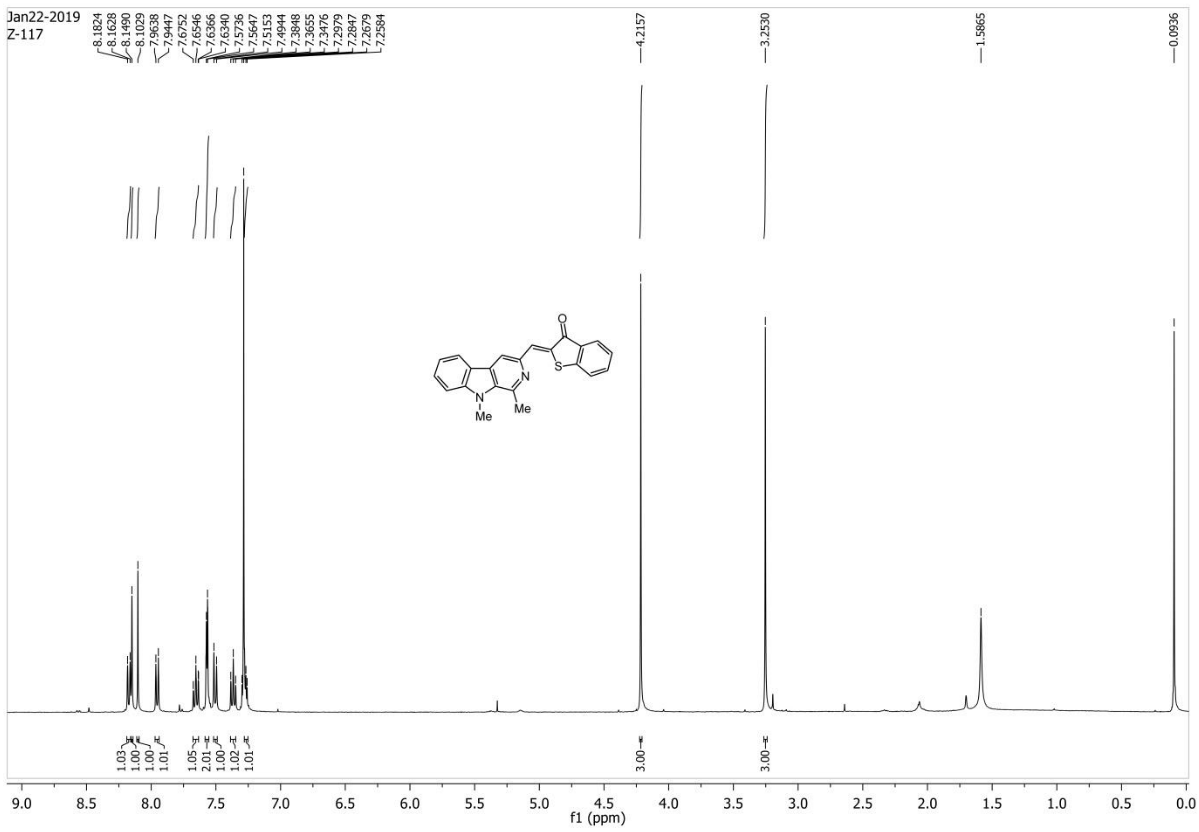

Figure S49. ^{1}H NMR spectrumof **4bA**.

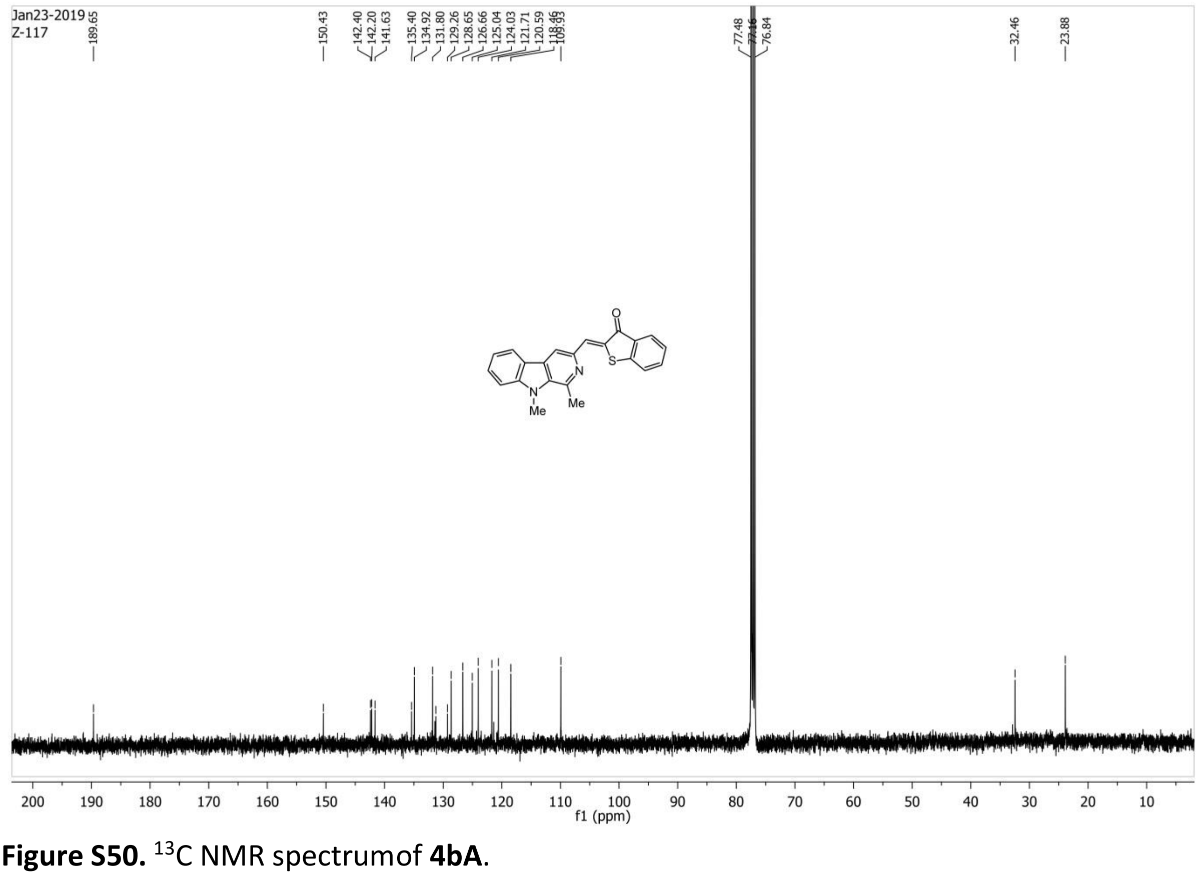

Figure S50. ^{13}C NMR spectrumof **4bA**.

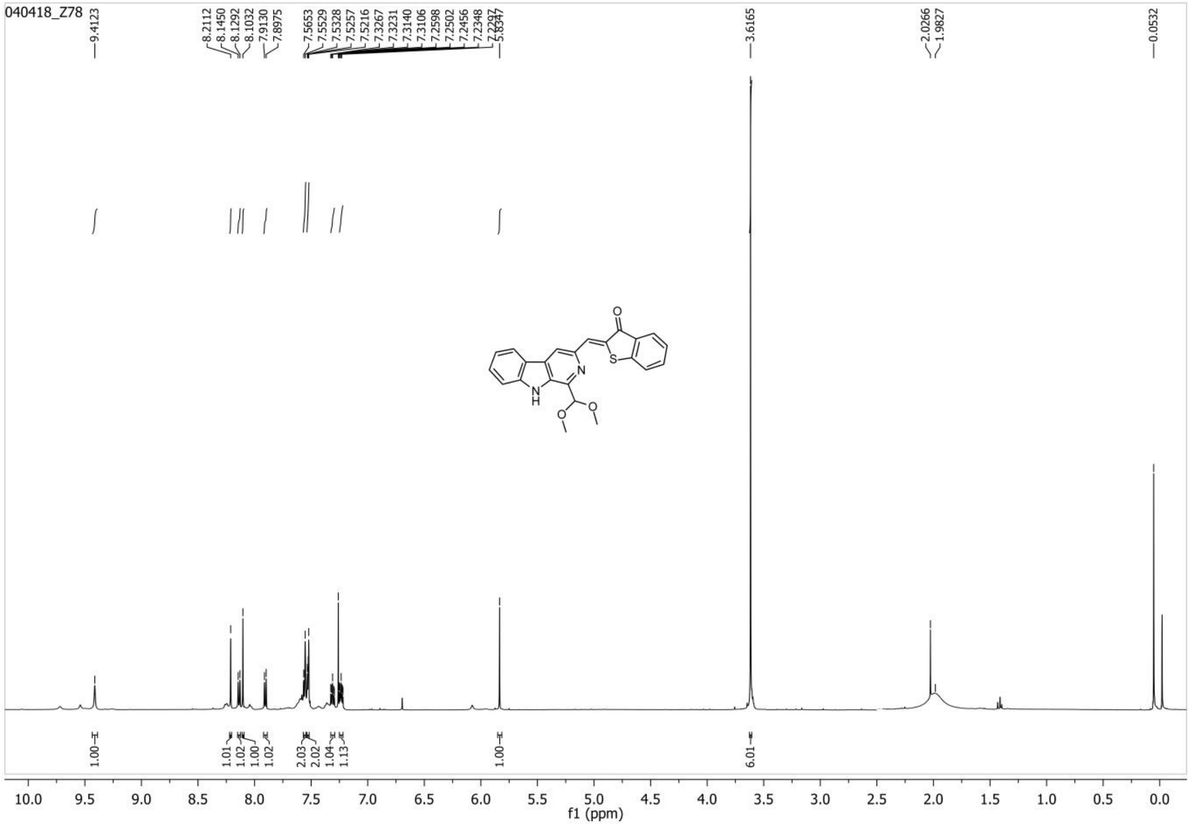

Figure S52. ^{1}H NMR spectrumof **4cA**.

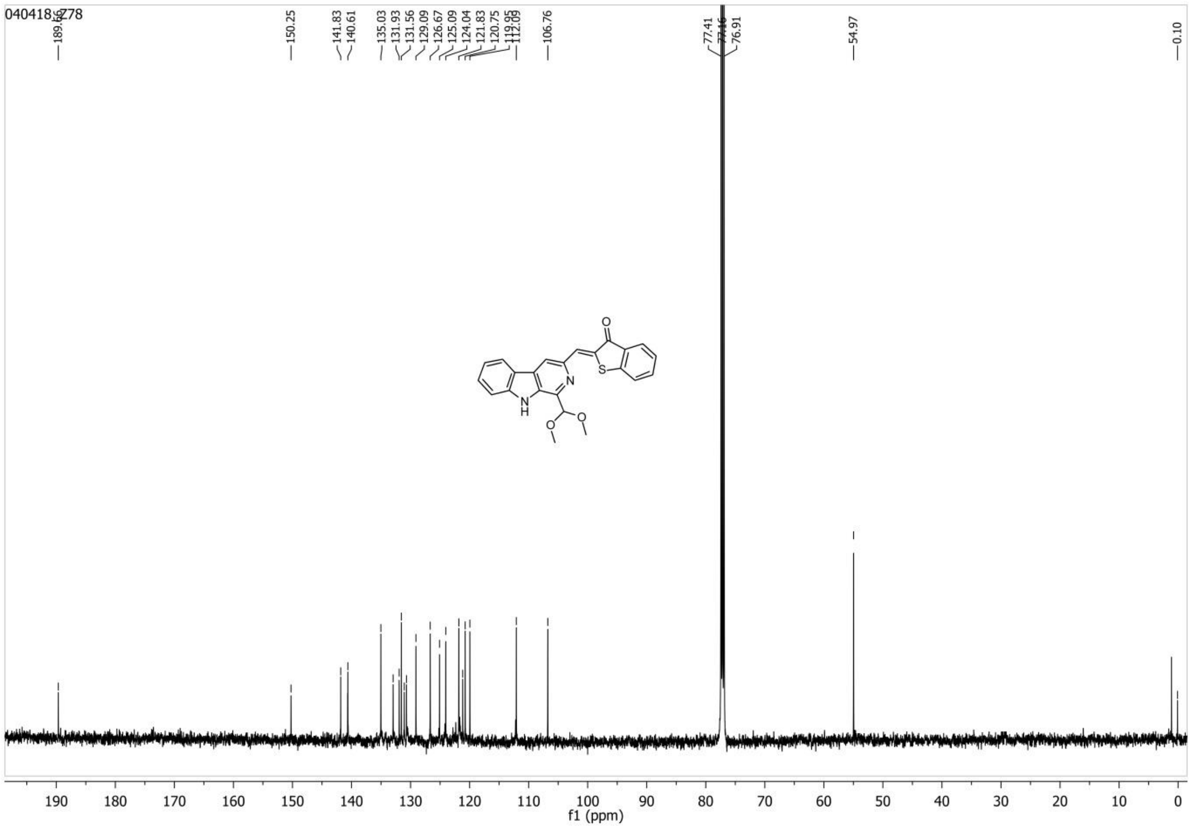

Figure S52. ^{13}C NMR spectrumof **4cA**.

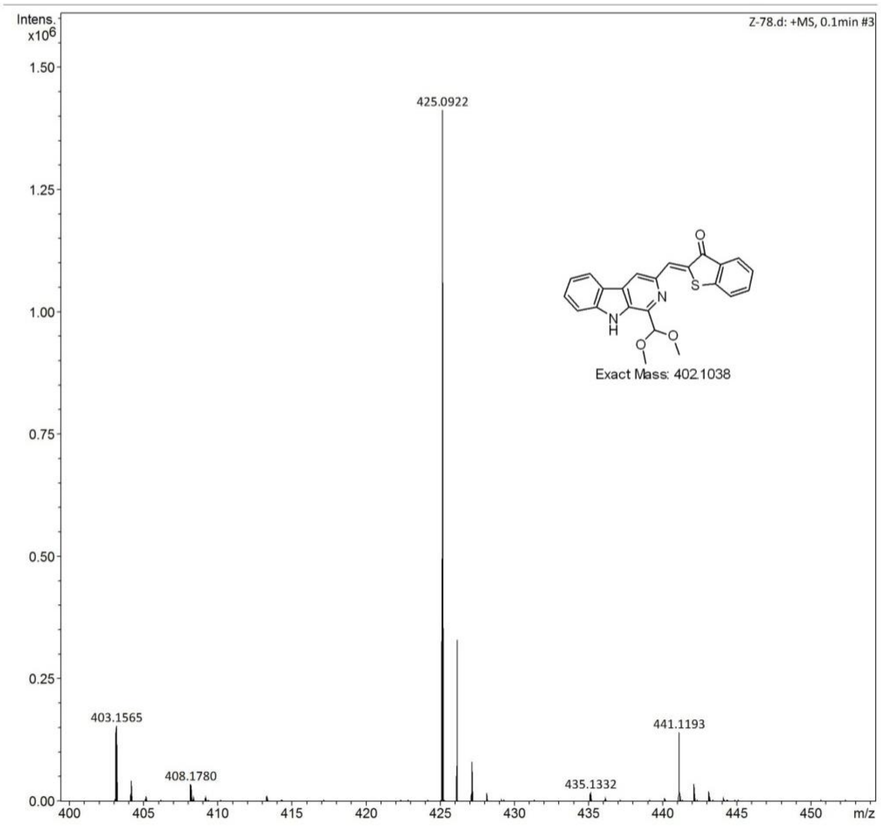

Figure S53. HRMS spectrum of **4cA**.

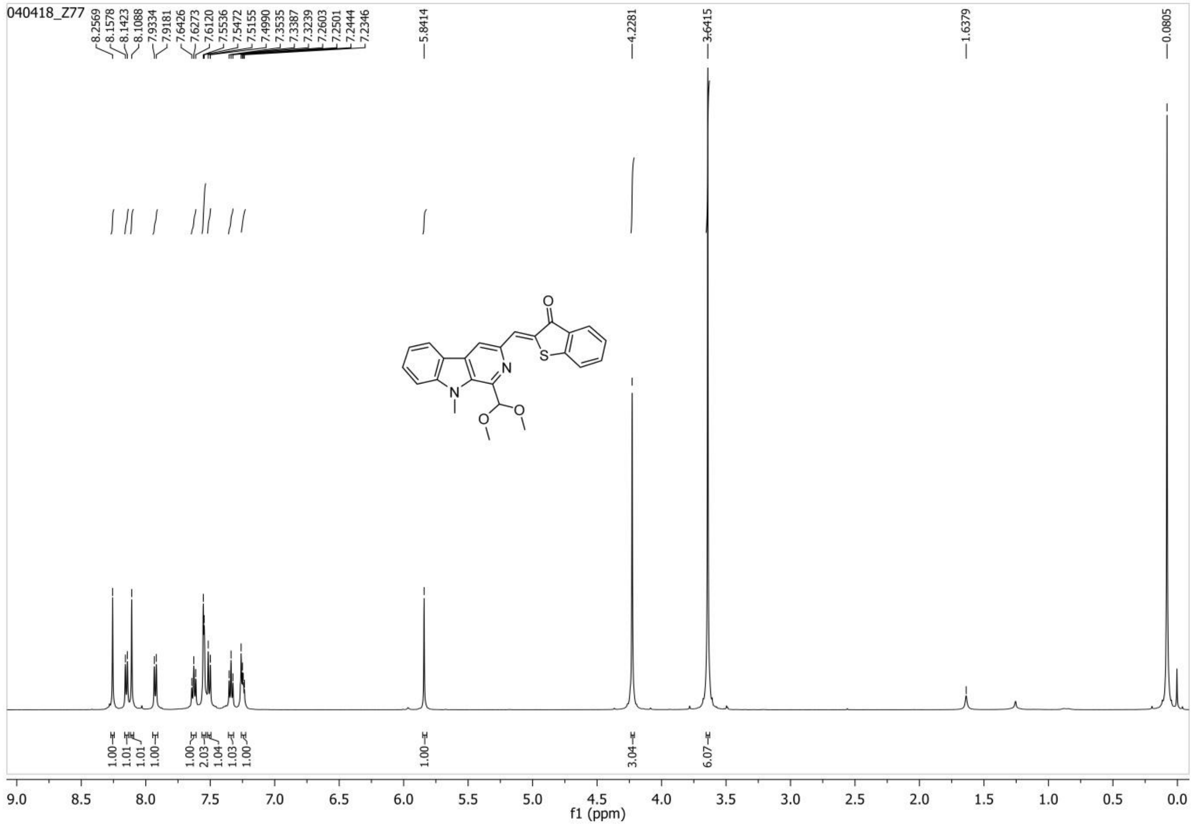

Figure S54. ^{1}H NMR spectrumof **4dA**.

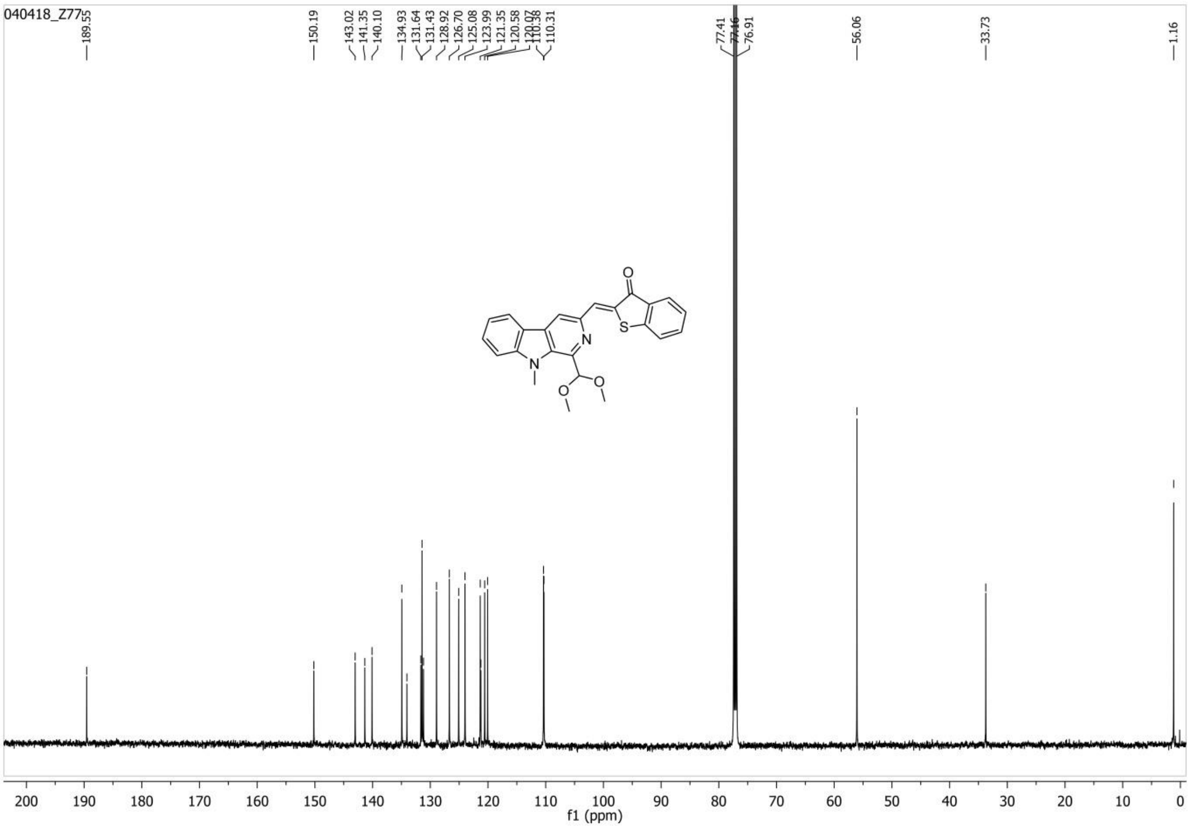

Figure S55. ^{13}C NMR spectrumof **4dA**.

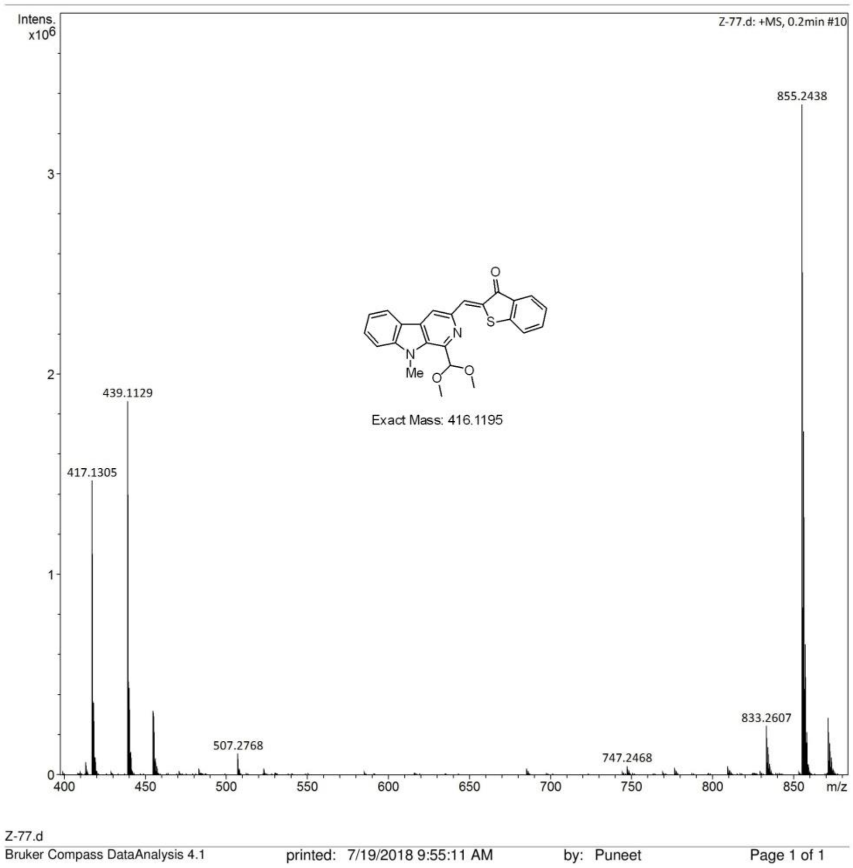

Figure S56. HRMS spectrum of **4dA**.

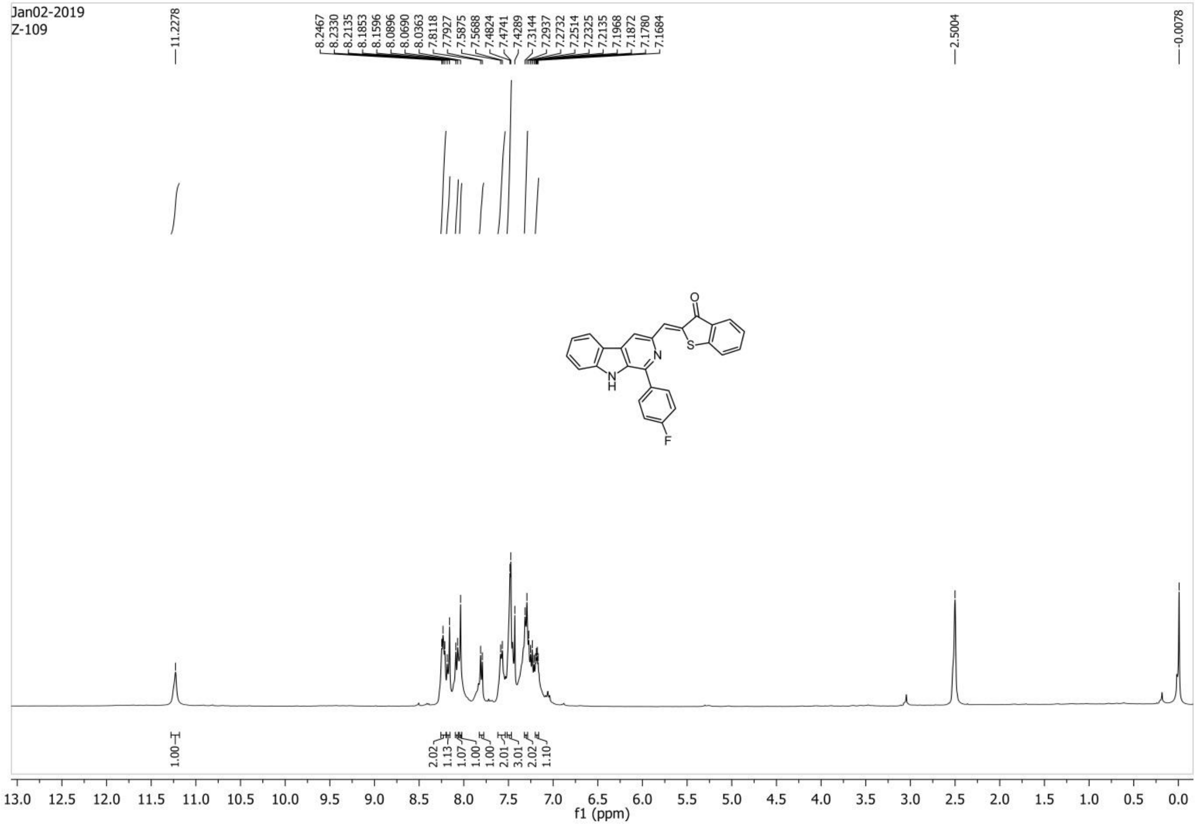

Figure S57. ^{1}H NMR spectrumof **4eA**.

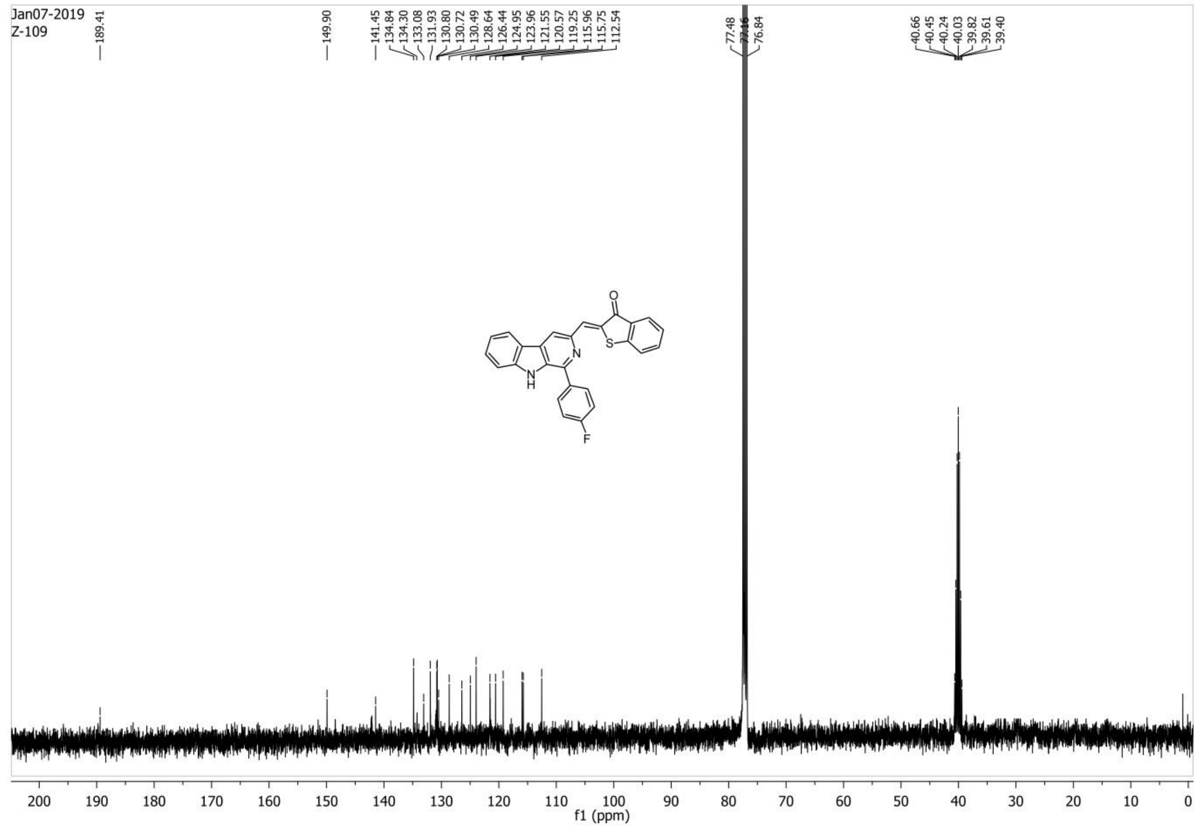

Figure S58. ^{13}C NMR spectrumof **4eA**.

Compound Report

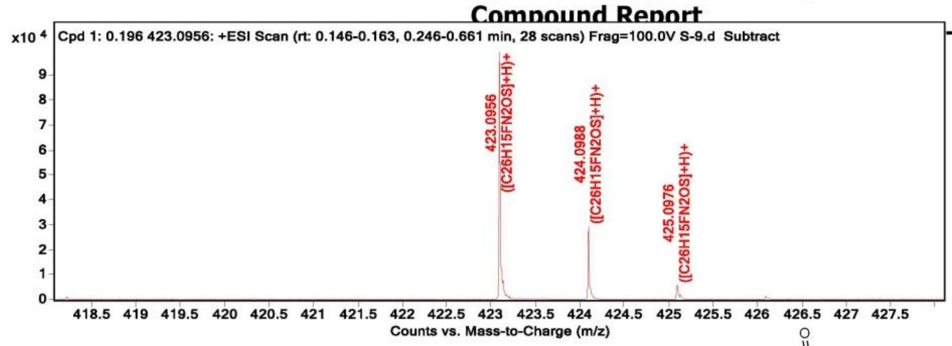

MS Spectrum Peak List

m/z	*Calc m/z*	Diff(ppm)	z	Abund	Formula	Ion
423.0956	423.0962	1.4	1	100747.47	C26H15FN2OS	(M+H)+
424.0988	424.0993	1.26	1	29414.15	C26H15FN2OS	(M+H)+
425.0976	425.0972	-1	1	5790.28	C26H15FN2OS	(M+H)+

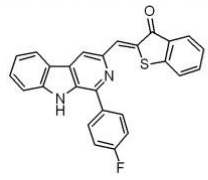

Exact Mass: 422.0889

Instrument Info : Agilent Technologies 6545 Q-TOF LC/MS

Note: Please acknowledge the work done by HRMS Facility at BITS Pilani, Pilani Campus funded by DST-FIST in your publication.

---End Of Report---

Figure S59. HRMS spectrum of **4eA**.

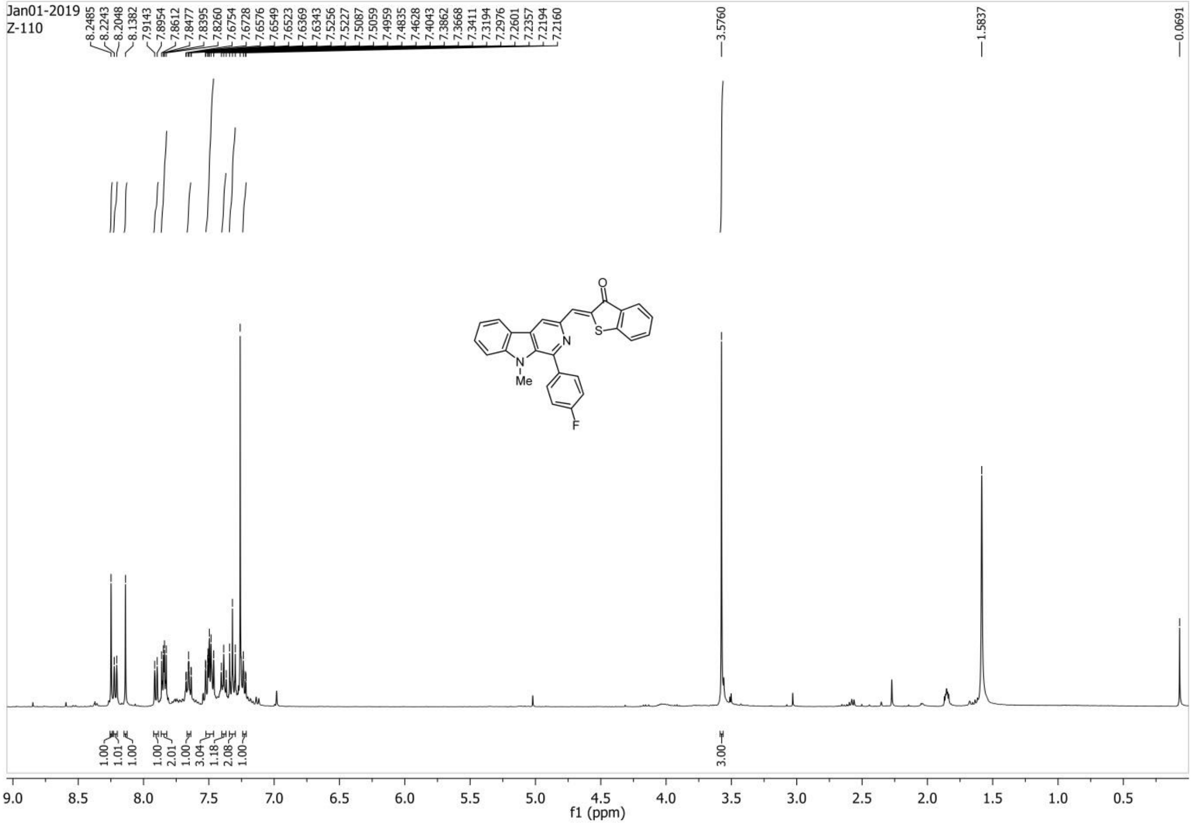

Figure S60. ^{1}H NMR spectrumof **4fA**.

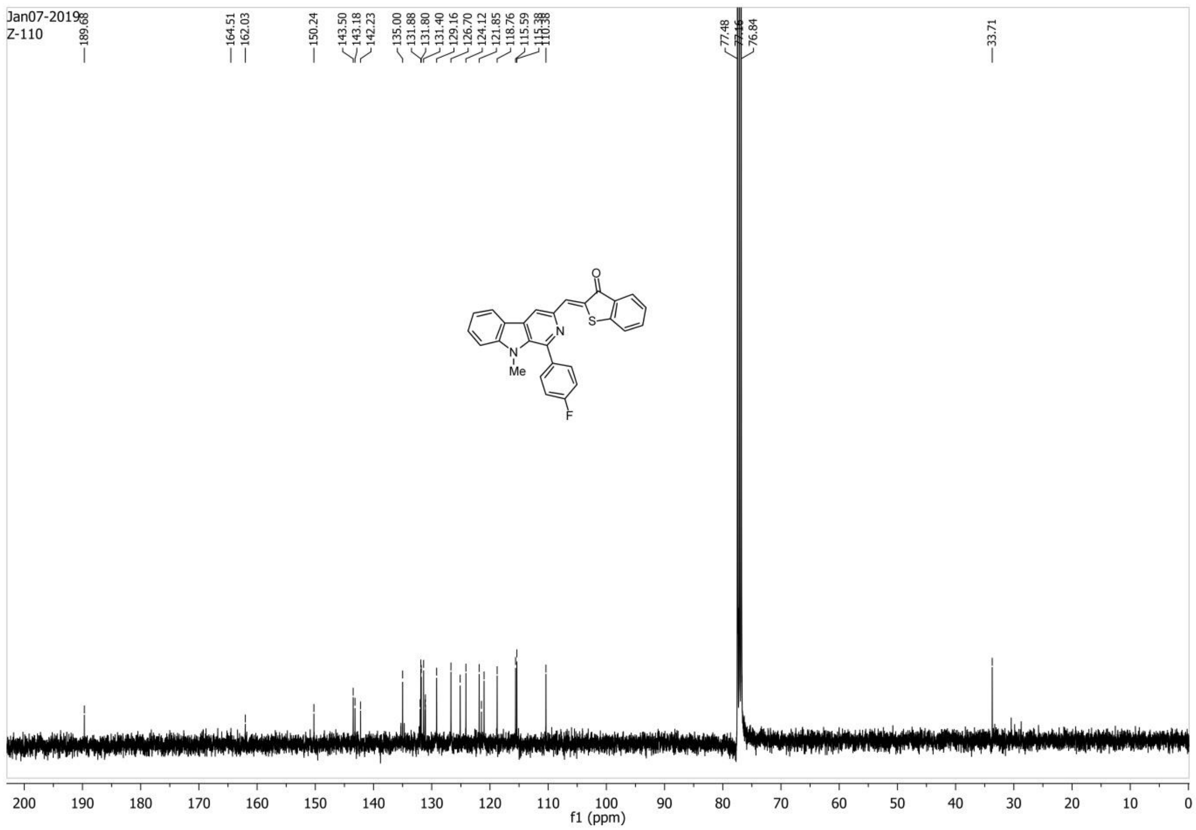

Figure S61. ^{13}C NMR spectrumof **4fA**.

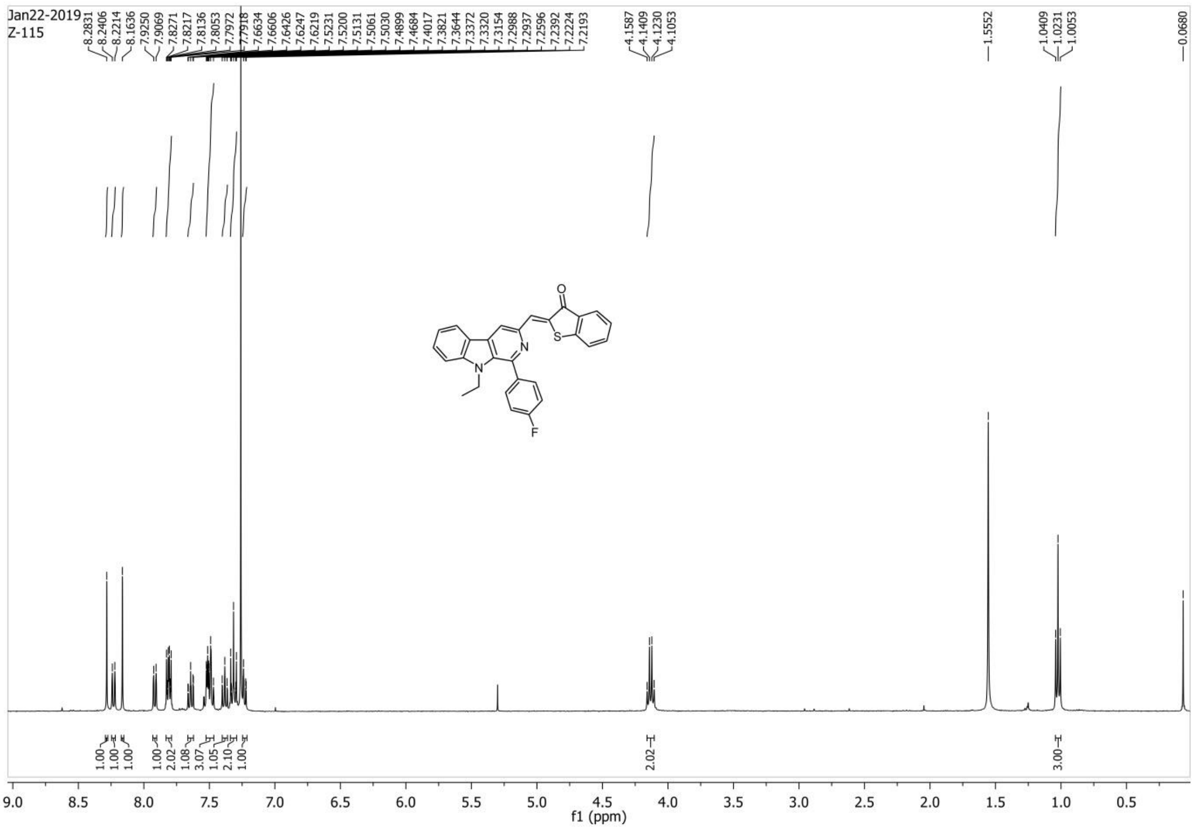

Figure S62. ^{1}H NMR spectrumof **4gA**.

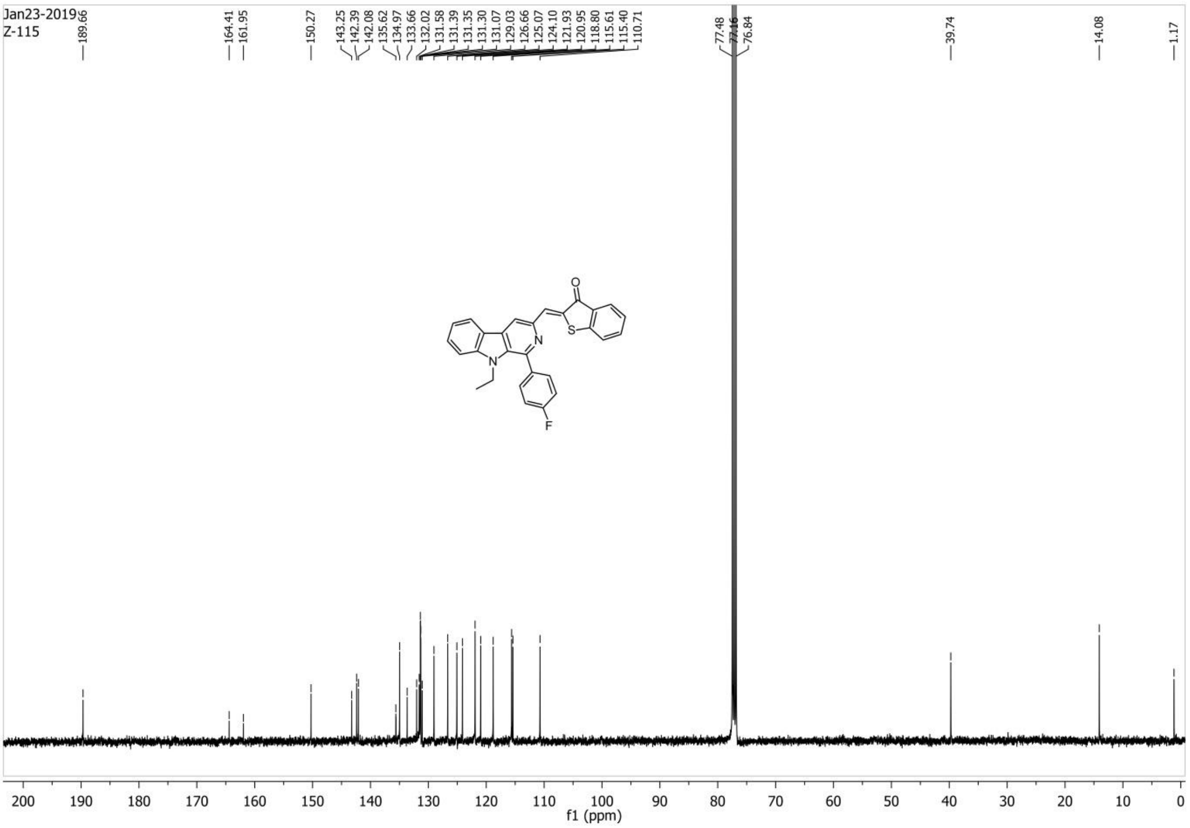

Figure S63. ^{13}C NMR spectrumof **4gA**.

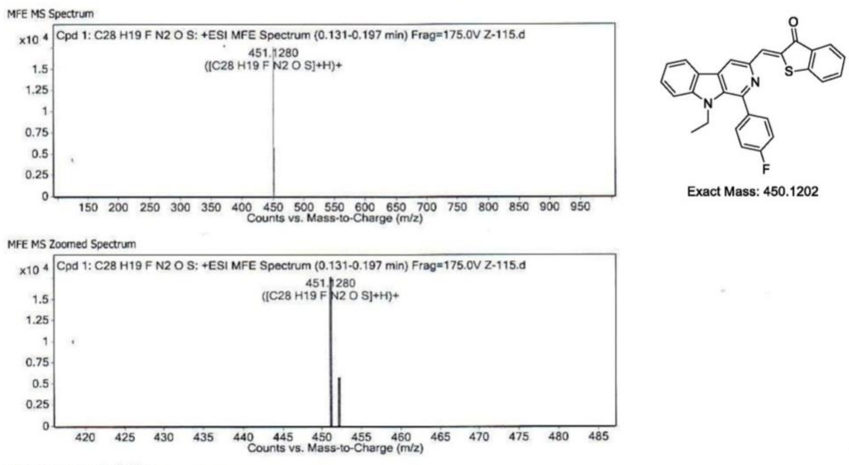

Compound Table

Compound Label	RT	Mass	Formula	MFG Formula	MFG Diff (ppm)	DB Formula
Cpd 1: C28 H19 F N2 O S	0.135	450.1212	C28 H19 F N2 O S	C28 H19 F N2 O S	-2.29	C28 H19 F N2 O S

Compound Label	m/z	RT	Algorithm	Mass
Cpd 1: C28 H19 F N2 O S	451.128	0.135	Find by Molecular Feature	450.1212

MS Spectrum Peak List

m/z	z	Abund	Formula	Ion
451.128	1	17600.21	C28 H19 F N2 O S	(M+H)+
452.1333	1	5679.85	C28 H19 F N2 O S	(M+H)+

--- End Of Report ---

Figure S64. HRMS spectrum of **4gA**.

Compound Report

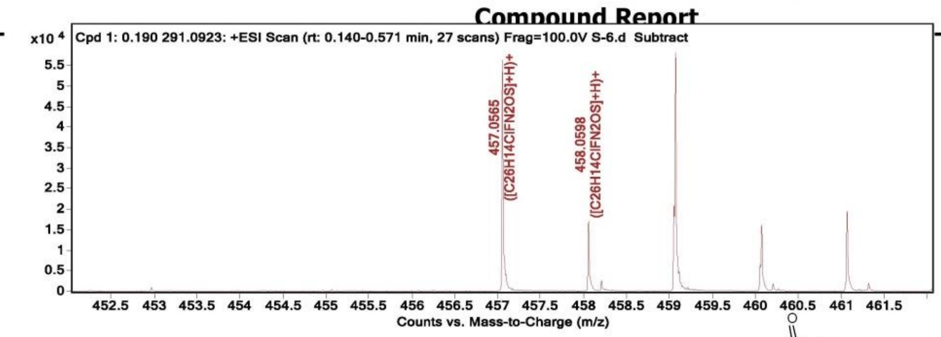

MS Spectrum Peak List

m/z	*Calc m/z*	Diff(ppm)	z	Abund	Formula	Ion
457.0565	457.0572	1.58	1	56911.99	C26H14ClFN2OS	(M+H)+
458.0598	458.0603	1.13	1	17287.38	C26H14ClFN2OS	(M+H)+

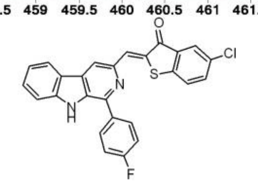

Exact Mass: 456.0499

Instrument Info : Agilent Technologies 6545 Q-TOF LC/MS

Note: Please acknowledge the work done by HRMS Facility at BITS Pilani, Pilani Campus funded by DST-FIST in your publication.

---End Of Report---

Figure S65. HRMS spectrum of **4eB**.

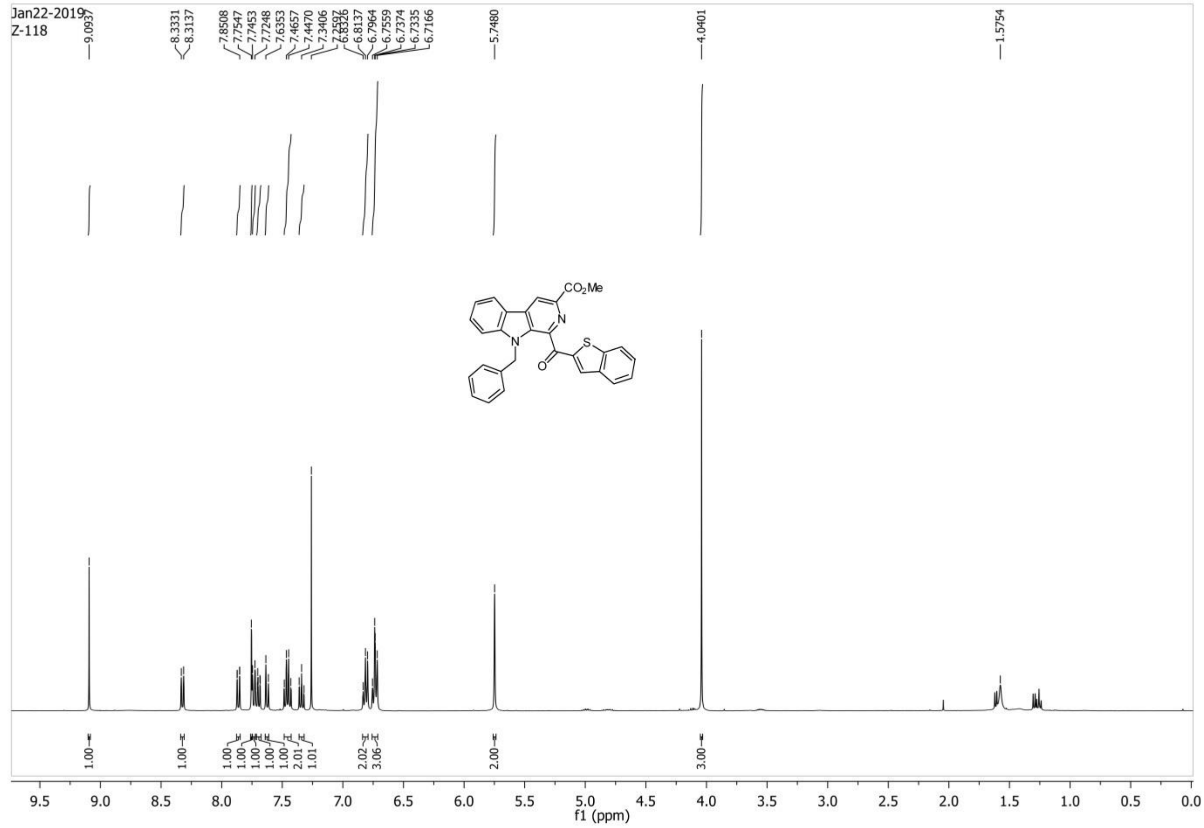

Figure S66. ^{1}H NMR spectrumof **6C**.

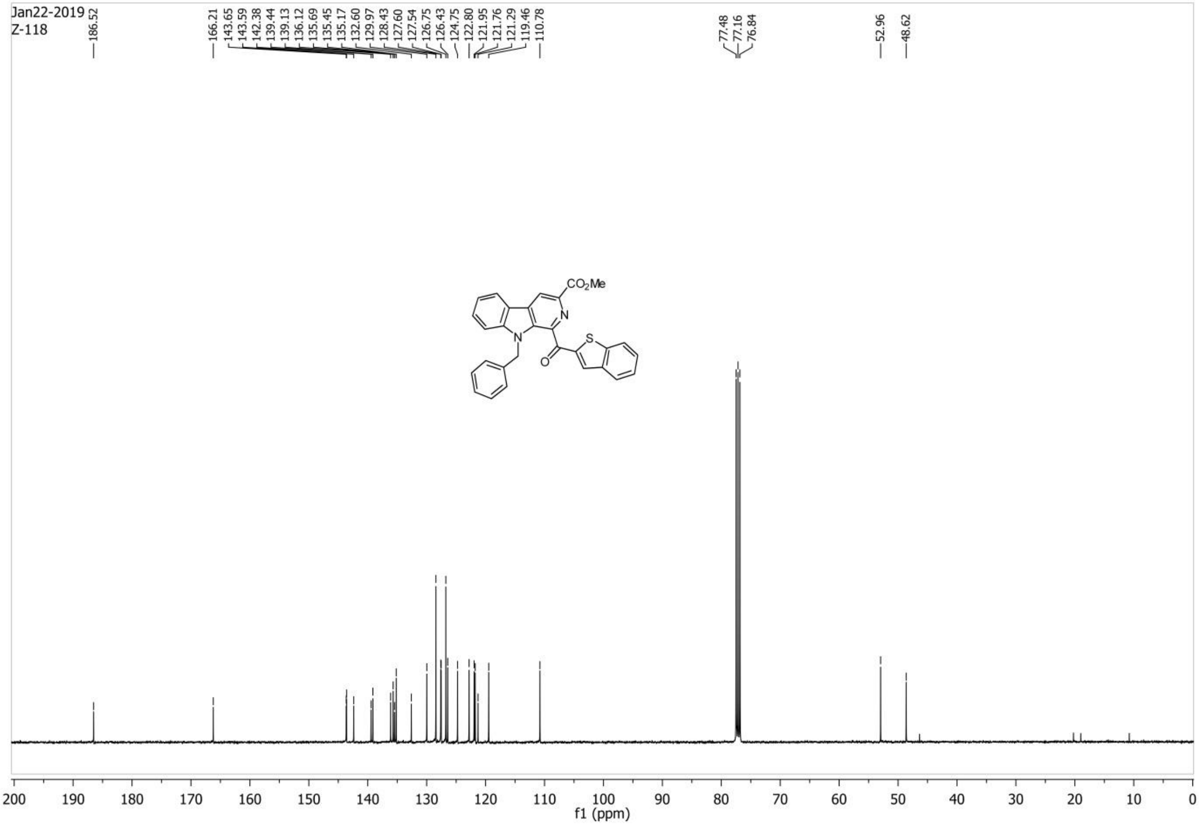

Figure S67. ^{13}C NMR spectrumof **6C**.

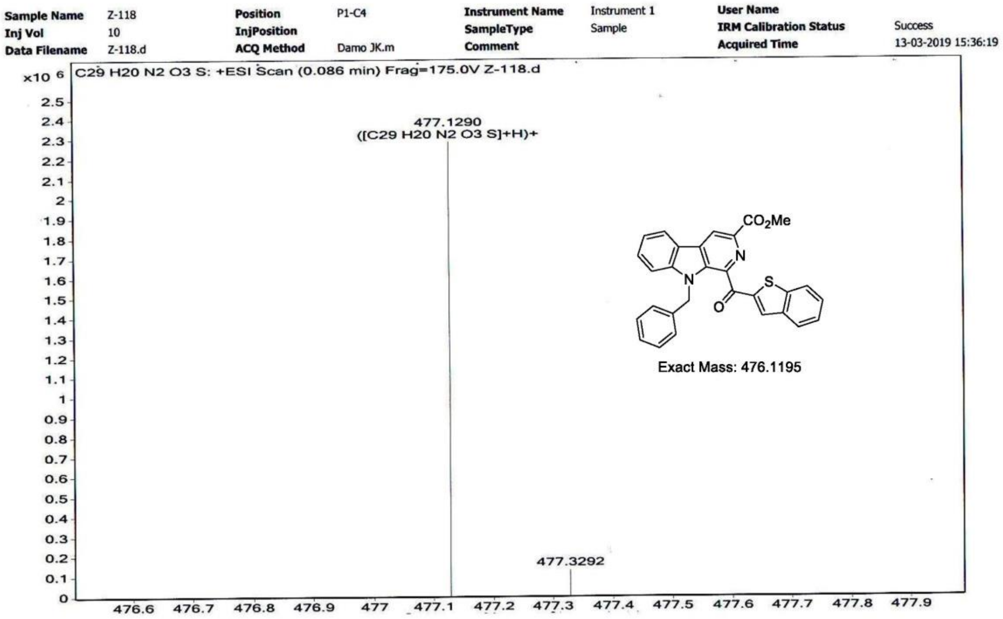

Figure S68. HRMS spectrum of **6C**.
